# Exploring the Role of Contactins across Psychological, Psychiatric and Cardiometabolic Traits within UK Biobank

**DOI:** 10.3390/genes11111326

**Published:** 2020-11-10

**Authors:** Julia Morris, Soddy Sau Yu Leung, Mark E.S. Bailey, Breda Cullen, Amy Ferguson, Nicholas Graham, Keira J. A. Johnston, Donald M. Lyall, Laura M. Lyall, Joey Ward, Daniel J. Smith, Rona J. Strawbridge

**Affiliations:** 1 Institute of Health and Wellbeing, University of Glasgow, Glasgow G12 8RZ, UK; 2113744M@student.gla.ac.uk (J.M.); soddy@connect.hku.hk (S.S.Y.L.); breda.cullen@glasgow.ac.uk (B.C.); afergus8@exseed.ed.ac.uk (A.F.); Nicholas.Graham@glasgow.ac.uk (N.G.); 2340814J@student.gla.ac.uk (K.J.A.J.); donald.lyall@glasgow.ac.uk (D.M.L.); Laura.Lyall@glasgow.ac.uk (L.M.L.); Joey.Ward@glasgow.ac.uk (J.W.); Daniel.Smith@glasgow.ac.uk (D.J.S.); 2 School of Life Sciences, College of Medical, Veterinary & Life Sciences, University of Glasgow, Glasgow G12 8QQ, UK; mark.bailey@glasgow.ac.uk; 3 Deanery of Molecular, Genetic and Population Health Sciences, College of Medicine and Veterinary Medicine, University of Edinburgh, Edinburgh EH8 9AG, UK; 4 Health Data Research UK, Glasgow G12 8RZ, UK; 5 Cardiovascular Medicine Unit, Department of Medicine Solna, Karolinska Institutet, 171 77 Stockholm, Sweden

**Keywords:** contactins, psychiatric disorders, cardiometabolic diseases, hypertension, Type 2 diabetes, genetic variation, single nucleotide polymorphisms, UK Biobank, gene expression

## Abstract

Individuals with severe mental illness have an increased risk of cardiometabolic diseases compared to the general population. Shared risk factors and medication effects explain part of this excess risk; however, there is growing evidence to suggest that shared biology (including genetic variation) is likely to contribute to comorbidity between mental and physical illness. Contactins are a family of genes involved in development of the nervous system and implicated, though genome-wide association studies, in a wide range of psychological, psychiatric and cardiometabolic conditions. Contactins are plausible candidates for shared pathology between mental and physical health. We used data from UK Biobank to systematically assess how genetic variation in contactin genes was associated with a wide range of psychological, psychiatric and cardiometabolic conditions. We also investigated whether associations for cardiometabolic and psychological traits represented the same or distinct signals and how the genetic variation might influence the measured traits. We identified: A novel genetic association between variation in *CNTN1* and current smoking; two independent signals in *CNTN4* for BMI; and demonstrated that associations between *CNTN5* and neuroticism were distinct from those between *CNTN5* and blood pressure/HbA1c. There was no evidence that the contactin genes contributed to shared aetiology between physical and mental illness

## 1. Introduction

Individuals with severe mental illness (SMI, such as schizophrenia, bipolar disorder and major depressive disorder) have an increased risk of cardiovascular and metabolic diseases (CMD), compared to the general population [1,2,3]. Indeed CMD (including obesity, type 2 diabetes, coronary artery disease and stroke) is a key factor in the reduced life expectancy observed for those with severe mental illness (typically 15–20 years for schizophrenia and 10–15 years for bipolar disorder [4]). Some risk factors for CMD are more common in severe mental illness, (such as physical inactivity, poor diet, high alcohol consumption and cigarette smoking), or worsen with duration of SMI (such as low socioeconomic status) and the side effects of psychotropic drugs (particularly atypical antipsychotics) further increase CMD risk. There is also growing evidence to support the existence of shared biological mechanisms [1,4,5,6,7], with observational studies proposing mechanisms such as disrupted circadian rhythms, hypothalamic–pituitary axis dysfunction and abnormal inflammation [8]. If specific components of shared mechanisms for CMD and SMI can be identified, there is potential for better prevention and treatment.

The family of contactin genes (*CNTNs*) represents a possible common mechanism between mental and physical illness because genome-wide association studies (GWAS) have implicated contactins in a wide variety of cardiometabolic and mental health conditions, summarized in Table 1. It has yet to be determined whether the same or distinct genetic signals influence the cardiometabolic and psychiatric/behavioural traits. Apart from these recent GWAS findings, the study of contactins has so far been predominantly from a neurobiology perspective. Expression of contactins has been observed in neurons, oligodendrocytes and oligodendrocyte precursors [9], and demonstrates involvement in cell contact formation, axonal growth control and pathfinding, axonal domain organisation, myelination, neuronal development and synaptogenesis [9]. Further, loss of contactin genes leads to malformed axons and impaired nerve conduction [9]. Contactin expression is not restricted to the brain, although understanding of functional effects of contactins outside of the brain is currently lacking.

In this study we used data from ~400,000 individuals from UK Biobank study to determine whether contactin genes contribute to a shared biology between CMD and SMI. Specifically, we (a) defined the impact of genetic variation in the contactin loci on psychiatric, psychological and cardiometabolic traits; (b) assessed in whether the genetic effects for CMD were distinct from, or shared with, those for SMI; and (c) we investigated how the genetic variants impacted on the measured phenotypes.

## 2. Materials and Methods

### 2.1. Genes

Genes encoding members of the contactin family were identified using NCBI Gene and chromosomal locations were defined by the UCSC genome browser (build 37). Regions of 400 kb up and downstream of each gene were analysed (Table 2).

### 2.2. Cohort

The UK Biobank has been previously described in detail [10]. In short, 502,000 individuals were recruited between 2006 and 2010 from 22 centres across the UK. All participants donated a blood sample for DNA analysis as well as completing a physical assessment and extensive online questionnaires detailing medical history (personal and family), lifestyle, education, and economic status. An online “Thoughts and feelings” questionnaire was sent to a subset of individuals (those who had consented to email contact) between 2016–2017. Only white British ancestry individuals were included in this study. This project was completed under UK Biobank applications 6533 (PI. DJS) and 1755 (PI. JPP).

### 2.3. Genotyping

UK Biobank participants were genotyped using either the Affymetrix UK Biobank Axiom or the Affymetrix BiLEVE Axiom array. Complete information regarding the genotyping, quality control, and imputation, which were conducted centrally by UK Biobank, has been published [10,11]. This study used the fully imputed genetic data, which was released in March 2018. SNPs were excluded for minor allele frequency (MAF) < 1%, imputation quality score < 0.4, deviation from the Hardy–Weinberg equilibrium (*p* < 5 × 10^−6^) or low call rate (<95%). Individuals were excluded for sex mismatch (reported vs. genetic), relatedness (one each pair of individuals with a KING-estimate kinship coefficient > 0.0442 was randomly removed), low call rate (<95%), non-white British ancestry (self-reported and based on principal component analysis) and quality control failure.

### 2.4. Phenotypes

The baseline mental health questionnaire in UK Biobank included questions to assess mood instability (“Does your mood often go up and down?” variable #1920) and risk-taking behaviour (“Would you describe yourself as someone who takes risks” variable #2040). Single item questions are an imperfect measure of complex psychological traits; however, their validity has been demonstrated relative to the expected associations with psychiatric disorders [12,13]. Additionally, the validity of the risk-taking question has been demonstrated relative to more detailed phenotyping [14]. Neuroticism was assessed using the Eysenck Personality Questionnaire (Revised Short Form) involving 12 yes/no questions (one of which was variable #1920 regarding mood instability). A yes response was coded 1, and these were added to give a score of between one and 12 for each individual. Of note, the mood instability question is a component of the neuroticism score.

Psychiatric disorder phenotyping was based on the online mental health (“Thoughts and Feelings”) questionnaire, completed between 2016 and 2017 (so between 6–9 years after baseline assessment) [15]. This questionnaire gathered information on history of major depressive disorder (MDD), bipolar disorder (BD), generalised anxiety disorder (GAD) and addiction. This enabled classification of likely lifetime experience of these disorders [15].

Cardiometabolic phenotypes (body mass index (BMI), waist and hip circumferences, systolic and diastolic blood pressure (SBP and DBP respectively)) were assessed in a standard manner. Waist-to-hip circumference adjusted for BMI (WHRadjBMI) was calculated as per Shungin et al. [16]. Average SBP and DBP were adjusted to reflect treatment-naïve levels, with addition of 15 mmHg and 10 mmHg respectively, for those on anti-hypertensive medication prior to analysis [17]. Type 2 diabetes (T2D) was defined as per Eastwood et al. [18]. Current smoking was assessed by questionnaire (variable #20116) and analysed as a binary trait (Current smokers vs. never and former smokers). Cardiovascular disease (CVD) was defined as of heart attack/myocardial infarction or angina (variable # 6150, assessed with the question “has your doctor told you that you have/have had a heart attack/myocardial infarction or angina”).

### 2.5. Genetic Analyses

All continuous phenotypes (except WHRadjBMI) were normally distributed and none required transformation prior to analysis. The calculation of WHRadjBMI is normalised, sex-specific, and includes adjustment for population stratification and age.

Genetic variants (specifically single nucleotide polymorphisms, SNPs) in the contactin loci were selected after genetic quality control. In the six contactin loci, 23,225 SNPs were available for analysis. Pairwise analysis and pruning (PLINK 1.07 [19], -indep-pairwise command with default settings (50 bp, 5 bp shift, LD r2 0.5)) resulted in 8008 independent SNPs. Genotype–phenotype analyses were conducted in Plink v1.90 [20] using linear or logistic regression as appropriate (for continuous and binary traits respectively), assuming additive allelic effects. With the exception of WHRadjBMI, all analyses were adjusted for age, sex, population structure (8 principal components), and genotyping chip. BMI and T2D were also adjusted for CVD case-control status. WHRadjBMI was only adjusted for CVD case-control status. We defined Bonferroni-corrected significant associations as those with *p* < 6.24 × 10^−6^ (8008 independent SNPs) and suggestive evidence of association as *p* < 1 × 10^−5^. Conditional analyses, where the lead SNP was included as a covariate, was conducted to determine whether there were any additional independent signals in the locus.

### 2.6. Data Mining

Linkage disequilibrium (LD) calculations were conducted using a randomly selected sample of 5000 unrelated white British ancestry individuals from UK Biobank, using Haploview [21]. Due to substantial computing requirements, 5000 individuals was considered sufficient. The Genotype Tissue Expression project (GTEx portal) was used to explore tissue expression patterns and genotype-specific effects on tissue expression of contactin genes [22]. The GWAS catalogue was used to identify previously reported associations with members of the CNTN family (20201008). All SNPs within the *CNTN* family with suggestive (*p* < 1 × 10^−5^) or genome-wide evidence (*p* < 5 × 10^−8^) of association with at least one phenotype were assessed for predicted functional effect using Variant Effect Predictor software (VEP) [23].

## 3. Results

The cohort characteristics are presented in Table 3.

The significant associations between SNPs in the contactin loci and CMD- or SMI-related phenotypes are summarised in Table 4.

### 3.1. Variation in CNTN Genes

**Figure 1:**
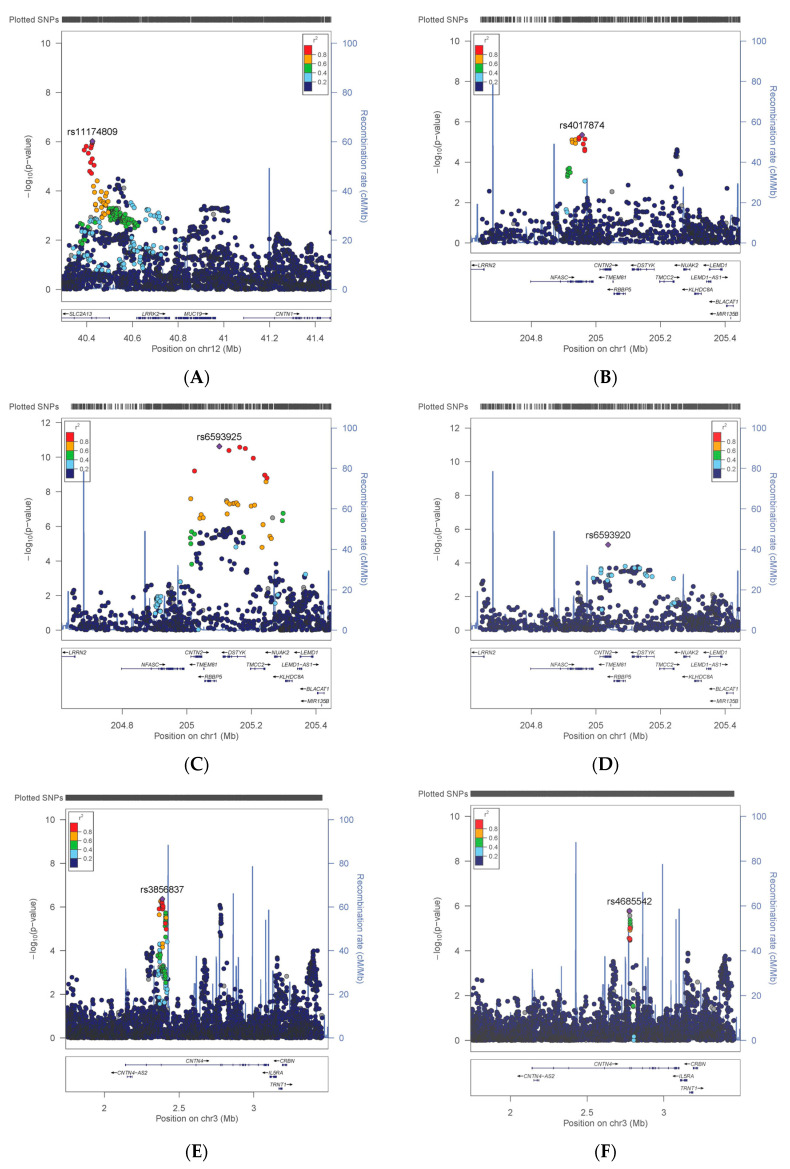

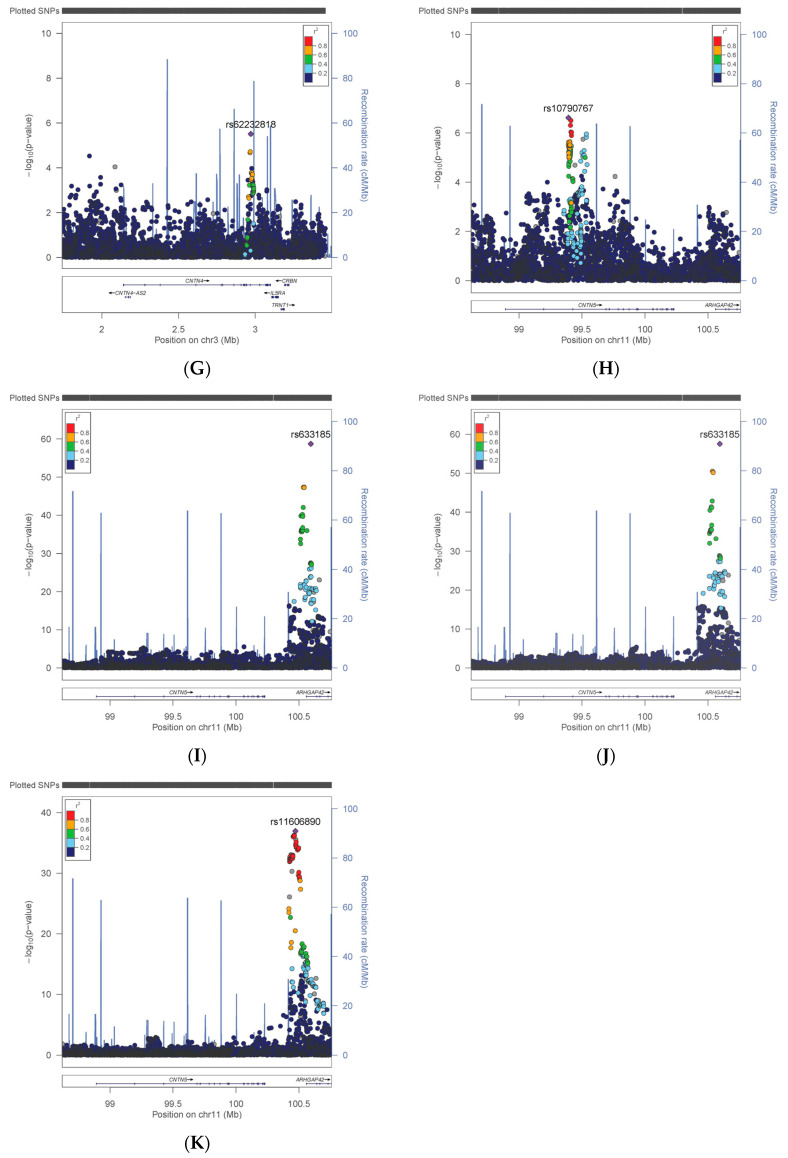
Regional plots demonstrating genetic associations between CNTN1 and (**A**) current smoking, CNTN2 and (**B**) risk-taking behaviour, (**C**) WHRadjBMI and (**D**) WHRadjBMI conditioned on rs6593925, CNTN4 and (**E**) BMI, (**F**) BMI conditioned on rs3856837, (**G**) risk-taking behaviour and CNTN5 and (**H**) neuroticism score, (**I**) DBP, (**J**) SBP and (**K**) HbA1c.

At the *CNTN1* locus, 9 SNPs demonstrated significant associations with current smoking (lead SNP rs11174809, *p* = 9.52 × 10^−7^, Figure 1A, and Table S1). Analysis of linkage disequilibrium between these SNPs demonstrate that they represent the same signal (Figure S1A).

At the *CNTN2* locus, significant evidence of association was demonstrated for seven SNPs with risk-taking behaviour (lead rs35068223, *p* = 1.77 × 10^−7^, Figure 1B and Table S2) and 133 SNPs with WHRadjBMI (rs6593925, *p* = 2.38 × 10^−11^, Figure 1C). Further analysis of WHRadjBMI, conditioning on rs6593925, demonstrated a second signal with 70 significant associations (rs11240349, *p* = 3.98 × 10^−8^, Figure 1D). This SNP was only nominally significant (*p* = 0.0014) in the initial analysis; however, there are five SNPs which were significant in both the unconditional and conditional analyses (rs12048743, *p* = 2.86 × 10^−10^ and 2.90 × 10^−6^, respectively). Analysis of LD demonstrates that the risk-taking and WHRadjBMI signals are independent, and that the region has many small blocks of LD (Figure S1B).

For variants in the *CNTN4* locus, 58 significant associations were evident for BMI (lead rs3856837, *p* = 4.28 × 10^−7^, Figure 1E and Table S3). Further analysis of BMI, specifically conditioning on rs3856837, demonstrated a second significant signal ~360Kb downstream (rs4685542, *p* = 1.66 × 10^−6^, Figure 1F). One SNP was significantly associated with risk-taking behaviour (lead rs62232818, *p* = 3.09 × 10^−6^, Figure 1G). Analysis of LD structure of this region indicates that the two BMI signals and the risk-taking signal are distinct from each other (Figure S1C).

The *CNTN5* locus (Table S4) demonstrated significant associations for 64 variants with the neuroticism score (rs10790767, *p* = 2.43 × 10^−7^, Figure 1H), 409 variants with SBP (rs633185, *p* = 2.25 × 10^−58^, Figure 1I), 395 variants with DBP (rs633185, *p* = 1.94 × 10^−59^, Figure 1J) and 301 with HbA1C (rs11606890, *p* = 1.14 × 10^−37^, Figure 1K). As shown in Figure 1, the SNPs associated with neuroticism score did not overlap with those for SBP/DBP or HbA1c. Indeed, these signals are ~1Mbp apart and analysis of LD across this locus suggests that they are independent signals (Figure S1D). In contrast, there was a great deal of overlap between the SNPs associated with SBP and DBP. Whilst the signals associated with HbA1C overlap with those for SBP and DBP, the SNPs are significantly associated either with blood pressure, or with HbA1c, but not with both (Table S4). Analysis of LD in this region suggests that the signals for HbA1C and blood pressure are independent (LD R2 = 0.16). No significant associations were observed for SNPs in the *CNTN3* or *CNTN6* loci.

### 3.2. Comparison with Previous Findings

Previous findings are summarised in Table S5. Where it is possible to compare with previous reports, the effect directions observed in this study are, for the most part, consistent. Where inconsistency is observed, it is highly likely that differences in phenotyping and/or sample size are the reason. We identified consistent effect direction for SNPs in *CNTN5* and neuroticism [24], smoking [25], WHRadjBMI [16], and HbA1C [26], *CNTN4* and smoking [25], and *CNTN2* and T2D [27]. In the CNTN2 locus, rs3903399-T allele has previously been associated with increased WHRadjBMI [28], whereas the WHRadjBMI-increasing allele in this study was rs3903399-C (Table S5). The rs3903399-T has also been associated with increased high density lipoprotein (HDL) cholesterol levels [29], which would be consistent with our findings for WHRadjBMI.

### 3.3. Effect Prediction in VEP

In order to identify any SNPs with predicted functional effects (that is those expected to change the protein sequence or quantity), all SNPs within the *CNTN* loci with significant evidence of association with at least one phenotype, were assessed for predicted functional effect using the Variant Effect Predictor [23] (Table S6). Only one missense variant was identified (rs3851294), but this is predicted to be benign/tolerated. The remaining variants had no clear functional effect.

### 3.4. Gene Expression Patterns

**Figure 2:**
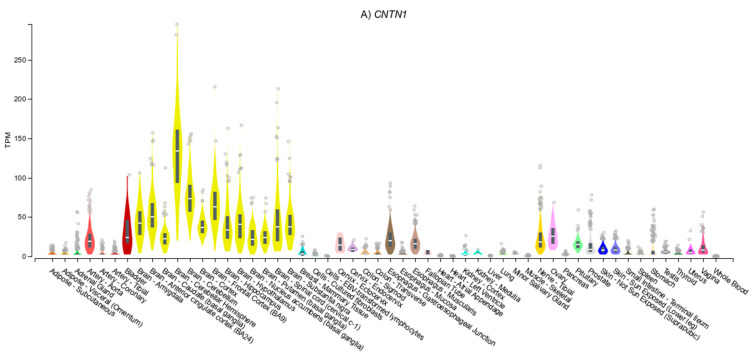

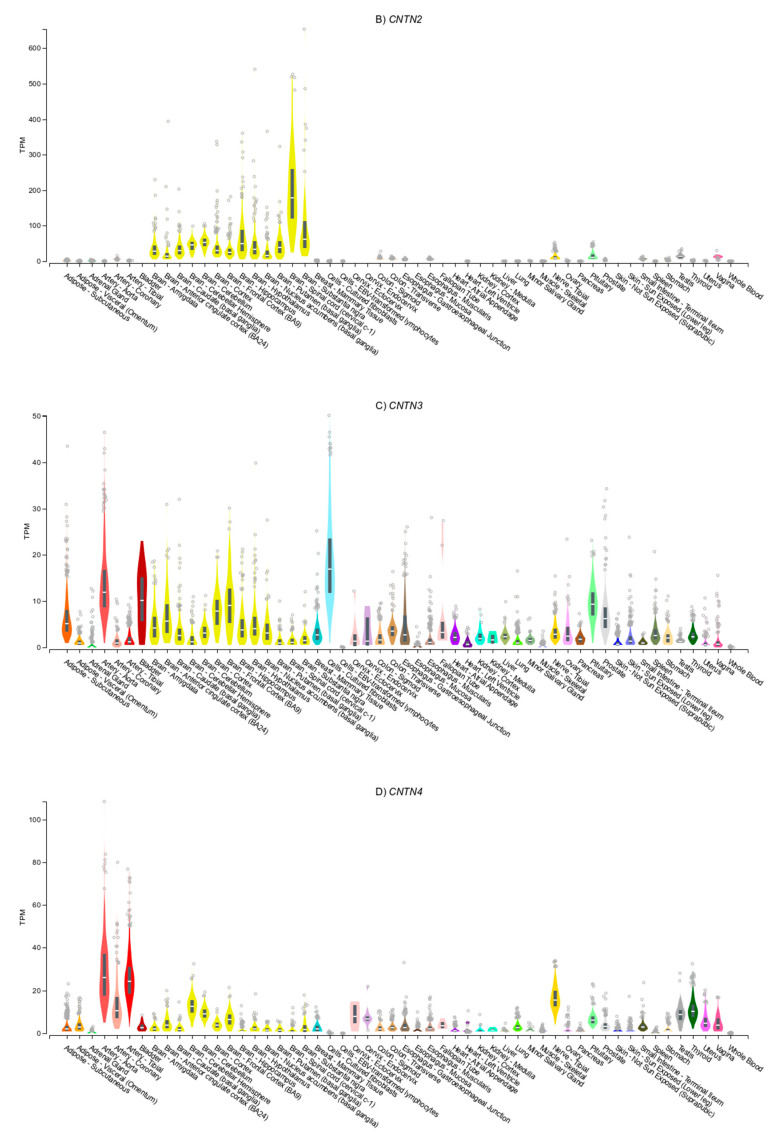

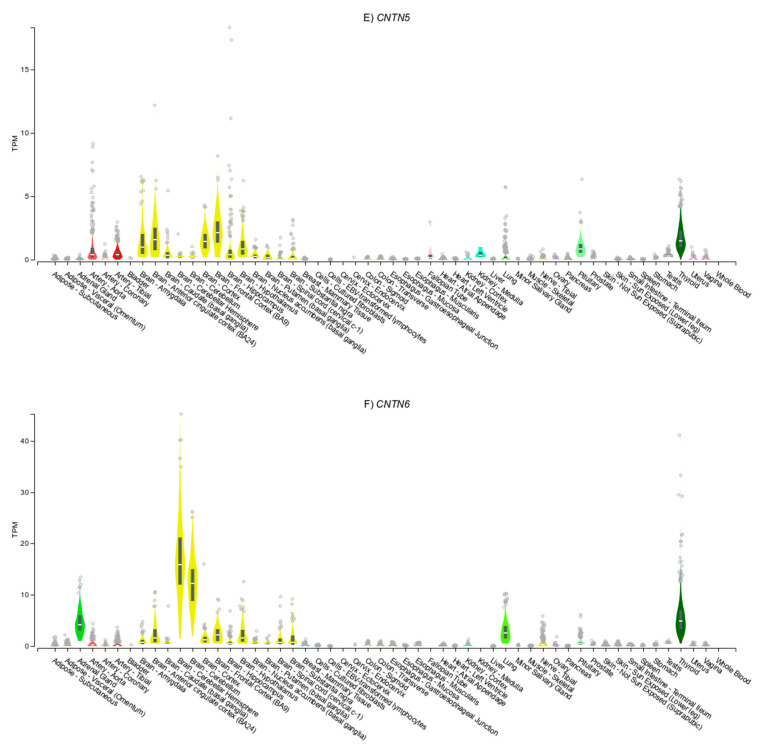
Tissue expression profiles of members of the CNTN family in the GTex dataset(**A**) *CNTN1*, (**B**) *CNTN2*, (**C**) *CNTN3*, (**D**) *CNTN4*, (**E**) *CNTN5* and (**F**) *CNTN6*.

Tissue expression patterns of the *CNTN* genes are presented in Figure 2. Expression of *CNTN1* (Figure 2A) is highest in the brain, therefore the association of SNPs in this gene with a behavioural trait such as smoking is plausible. *CNTN2* is predominantly expressed in the brain (Figure 2B), which is consistent with effects on behavioural traits such as risk-taking. The effect on WHRadjBMI is less clear but could be indirectly through influencing eating preferences and behaviours. Whilst there is evidence to suggest that genetic variants associated with WHRadjBMI act through expression of genes in adipose tissue, [16], there is significant [16,30] genetic overlap of this trait with BMI, whereby genetic variants are through to act via gene expression in the brain [30]. In contrast, *CNTN4* is widely expressed (Figure 2D), with brain (consistent with risk-taking behaviour) and adipose (consistent with BMI) expression being observed. The expression of *CNTN5* is predominantly observed (Figure 2E) in the brain (consistent with neuroticism). Whilst *CNTN5* expression was also observed in arterial tissue (consistent with SBP and DBP) and the pituitary gland (consistent with HbA1c).

### 3.5. Genotype-Specific Expression Patterns for Associated SNPs (eQTLs)

Using the GTEx data, genetic variants with genotype-specific effects on *CNTN* gene expression were identified (Table S7). Of the eQTLs identified for the contactin family, only those for *CNTN2* expression levels overlapped with significant associations in these analyses. The missense SNP rs3851294 (*CNTN2*, associated with WHRadjBMI) shows effects on *CNTN2* expression in the thyroid and oesophageal muscle. Additional eQTL SNPs for *CNTN2* which overlap with those significantly associated with risk-taking behaviour or WHRadjBMI demonstrated effects in a variety of tissues, which is curious given that *CNTN2* expression is predominantly in the brain (Table S7). How expression of *CNTN2* levels in the oesophagus, nerve, skin or thyroid influence risk-taking behaviour or WHRadjBMI is unclear. Irrespective of the tissue analysed, the WHRadjBMI-lowering allele was associated with reduced levels of *CNTN2* expression. Of the SNPs in *CNTN5*, those associated with blood pressure and HbA1c are downstream of *CNTN5* and closer to *ARHGAP42* and *TMEM133* than to *CNTN5.* The lead SNPs for these signals have genotype-specific effects on expression of *ARHGAP42* and *TMEM133* in arterial and heart tissues (Table S8), suggesting that the effect of these signals are via *ARHGAP42* or *TMEM133* rather than *CNTN5*.

## 4. Discussion

This study, of multiple phenotypes in a single-protocol, very large population-based cohort, identified genomic loci for current smoking at *CNTN1*, risk-taking and WHRadjBMI at *CNTN2*, risk-taking and BMI at *CNTN4* and neuroticism, DBP/SBP and HbA1c at *CNTN5*. Loci for psychological traits were independent from those for cardio-metabolic traits, therefore these results do not support contactins as a putative biological link between mental and physical illness.

The association between *CNTN1* and current smoking is novel. This locus has previously been associated with neuro-psychiatric (Parkinson’s disease [31], Alzheimer’s disease [32], bipolar disorder [33] and antidepressant response [34]), but not behavioural traits. Whilst *CNTN1* gene expression is not limited to the brain, this study provides no evidence for a role of *CNTN1* in cardio-metabolic disease.

We provide replication of the association between *CNTN2* and risk-taking behaviour/adventurousness [35], however the association with a metabolic phenotype, specifically WHRadjBMI, is novel. *CNTN2* has previously been associated with WHR and schizophrenia [36,37,38]. Whilst it has been established that patients with schizophrenia have a tendency towards abdominal obesity [3,4], our results suggest that the signals for psychiatric and metabolic phenotypes are independent signals, with the schizophrenia-associated SNPs showing only nominal associations with BMI (lowest *p* = 0.0053).

Whilst an association between the *CNTN4* locus and longitudinal BMI has previously been reported [39], our demonstration of two independent signals for BMI is novel. In addition, the association of this region with risk-taking is novel. Although it has previously been demonstrated that genetic regulation of risk-taking overlaps with that for schizophrenia, and *CNTN4* has previously been associated with schizophrenia [12,40], the null association between schizophrenia-associated SNPs and risk-taking in this study suggests that the signals for these traits are independent.

The associations observed between *CNTN5* and neuroticism, blood pressure and HbA1c are not novel, however the demonstration that these signals are independent of each other is an advancement in understanding the impact of this locus on biology. It is worth noting there is a *cntn5*-knockout mouse model, which reported no behavioural phenotype but effects on obesity and blood pressure variables [41]. According to the NCBI genome data viewer (*mus musculus*), *arhgap42* overlaps the *cntn5* gene, therefore it is conceivable that the *cntn5*-knockout model also results in *arhgap42* loss of function and thus a phenotype consistent with that in humans.

This study did not find evidence for the effects of the contactin family on MDD; this is unlikely to be due to the number of cases present. One possibility is due to imprecise phenotyping: We used a measure of probable lifetime MDD; a confirmed diagnosis of MDD as per DSM or ICD criteria may have yielded different results. Investigating the locus in the Psychiatric Genetics Consortium (PGC) data was considered; however, the conclusions drawn would be less clear: The PGC data also using highly heterogeneous phenotype definitions, depending upon where the samples were collected, and covariates differ between cohorts and analyses. In addition, assessing the independence of signals (multiple signals for one trait or between multiple traits) is more difficult from summary statistics, and could be biased by the population structure/ancestry and/or how the population structure was accounted for in the analysis. Hence restricting the analysis to only UK Biobank meant that the assessment of genetic architecture of the contactin genes was robust, even if the lack of clinical diagnosis for psychiatric illness is a weakness. Additionally, due to the nature of individuals recruited to UK Biobank, there are unlikely to be many cases of severe MDD. Having a preponderance of mild/single episode depression cases could dilute the phenotype, therefore differences between MDD cases and controls (which potentially include subclinical or undiagnosed MDD) are relatively small.

MDD is a heterogenous phenotype [42,43,44]. Attempts to subdivide MDD [45] have included by clinical presentation (e.g., atypical, melancholic, psychotic), by timing of onset (age of onset, seasonality, postpartum), course (single episode, recurrent, chronic) and severity. Subtypes of MDD may have different genetic risk factors, for example, childhood-onset MDD is genetically more similar to schizophrenia and bipolar disorder than to adult-onset MDD [46]. However, large-scale studies assessing whether different clinical presentations of MDD have different genetic risk factors are in the early stages [47], despite increasing evidence for different biological pathways being implicated (HPA-axis dysregulation for melancholic depression and inflammation in atypical depression) [8,46,48]. It remains to be seen whether the *CNTN* family influences subtypes of depression specifically.

We did not find evidence of association between the contactin family and addiction, mood instability, bipolar disorder, ischaemic heart disease and type 2 diabetes. *CNTN5* has been considered a candidate gene for bipolar disorder [9], however we did not find evidence of an association, potentially because of the relatively small number of BD cases in this cohort.

## 5. Conclusions

In summary, our systematic analysis of genetic variation in the CNTN family of genes identified roles for *CNTN1*, *CNTN2, CNTN4* and *CNTN5* in a variety of psychological and cardiometabolic traits. The evidence presented here suggests that the effects of these genes on psychological and cardiometabolic traits are likely distinct, with no evidence of shared mechanisms.

## Figures and Tables

**Table 1:** Summary of reported associations with contactin loci.

**Locus**	**DISEASE/TRAIT**	**PMID**
*CNTN1*	Antidepressant treatment resistance (>2 drugs prescribed)	30700811
	Bipolar disorder	22925353
	Blood protein levels	30072576
	Cerebral amyloid deposition in APOEe4 non-carriers (PET imaging)	26252872
	Cognitive ability in schizophrenia	31206164
	Dementia and core Alzheimer’s disease neuropathologic changes	25188341
	IgG glycosylation	23382691
	Metabolite levels (X-11787)	23934736
	Parkinson’s disease	24842889
	Reaction time	29844566
*CNTN2*	Adventurousness	30643258
	Blood protein levels	29875488, 28240269
	Cerebrospinal fluid biomarker levels	28031287
	Highest math class taken	30038396, 30038396
	Red blood cell count	27863252
	Schizophrenia	30285260, 26198764, 28991256
	High density lipoprotein cholesterol levels	32203549
	Type 2 Diabetes	32541925
	Waist–hip ratio	30239722
*CNTN3*	Age at first sexual intercourse	27089180
	Economic and political preferences	22566634
	Educational attainment (years of education)	30038396
	Frontal fibrosing alopecia	30850646
	Heart rate response to recovery post exercise	29769521, 29497042
	Paediatric bone mineral content (hip)	28181694
	Peripheral arterial disease (traffic-related air pollution interaction)	27082954
	Smoking initiation and quantity	30643251, 24665060
	Systolic blood pressure	30224653
*CNTN4*	Adolescent idiopathic scoliosis	30019117
	Amyotrophic lateral sclerosis	19451621
	Atypical femoral fracture in phosphonate treatment	31006051
	White blood cell phenotypes	27863252, 29403010
	Bisphosphonate-associated atypical femoral fracture	31006051
	Blood pressure	17903302, 25189868
	Blood protein levels	30072576, 29875488
	Body Mass Index (change over time) in chronic obstructive pulmonary disease	28044437
	Brain connectivity	23471985
	Chronotype and sleep variables	30696823, 27126917, 30804565, 30595370
	Cognitive ability, years of educational attainment or schizophrenia (pleiotropy)	31374203
	Multiple psychiatric disorders	31835028, 28540026, 26198764, 30285260, 28991256, 30285260, 31268507, 31740837, 25056061
	DNA methylation variation (age effect)	30348214
	Gallbladder cancer	22318345
	Gut microbiota	32572223
	Immune biomaker levels	32066700, 27989323
	Intelligence	22449649
	Metabolite levels	23823483, 31005972, 24379826
	Middle childhood and early adolescence aggressive behaviour	26087016
	Monoclonal gammopathy of undetermined significance	30134812
	Pit-and-fissure caries	24556642
	Post bronchodilator FEV1/FVC ratio	26634245
	Response to aripiprazole in schizophrenia	29503163
	Self-reported math ability	30038396, 30038396
	Sensorimotor dexterity	31596458
	Smoking initiation (ever regular vs. never regular)	30643251
	Stimulated adipocyte lipolysis	2180562
	Tuberculosis	28928442
*CNTN5*	Blood cell phenotypes	32888493, 27863252, 30595370, 28957414
	Protein levels in obesity	29234017
	Bipolar and/or Depressive disorders	31926635, 31969693, 27089181, 29942085, 30643256, 29292387
	Carotid intima media thickness interaction with smoking	32117412
	Adolescent idiopathic scoliosis	30019117
	Alcohol dependence	26365420
	Atrial fibrillation	17903304
	Blood protein levels	29875488, 30072576, 28240269
	Bone mineral density (femoral neck)	26911590
	Chronotype and sleep phenotypes	30696823, 30804565, 30595370
	Core binding factor acute myeloid leukaemia	27903959
	Educational attainment (MTAG)	30038396
	Factor VII activity	30642921
	Feeling miserable	29500382
	Glycated haemoglobin levels	28898252
	Gout vs. Hyperuricemia	31289104
	Immune response to smallpox (secreted IL-2)	22610502
	Interleukin-2 receptor antagonist levels	27989323
	Life satisfaction	30643256
	Loneliness (multivariate analysis)	27629369
	Lung function (FEV1/FVC)	30595370
	Menarche (age at onset)	23667675
	Metastasis in stage I-III microsatellite instability low/stable colorectal cancer (time to event)	30738427
	Myopia (pathological)	23049088
	Neurological blood protein biomarker levels	31320639
	Neuroticism	30595370, 29942085, 29292387, 30643256, 27089181, 29292387
	Objective response to lithium treatment in bipolar disorder	26503763
	Plasma kynurenine levels in major depressive disorder	29317604
	Positive affect	30643256
	Post bronchodilator FEV1/FVC ratio in COPD	26634245
	Reaction time	29844566
	Smoking initiation (ever regular vs. never regular) (MTAG)	30643251
	Spatial memory	31596458
	Suicidality	30745170
	Systolic blood pressure	30595370
	Trans fatty acid levels	25646338
	Waist–hip ratio	25673412
	Well-being spectrum (multivariate analysis)	30643256
	Volumetric brain MRI	17903297
*CNTN6*	Caudate activity during reward	28927378
	Depressive symptoms (stressful life events interaction)	27529621
	Diastolic blood pressure	28270201
	DNA methylation variation (age effect)	30348214
	Gut microbiota	31519223, 27723756, 32572223
	Iris colour (L* coordinate)	30895295
	Loneliness	27629369
	Metabolite levels	23823483, 22675492
	Neurocognitive impairment in HIV-1 infection (continuous)	28447399
	PR interval in Tripanosoma cruzi seropositivity	24324551
	Prostate cancer (SNP x SNP interaction)	22219177
	Subjective response to placebo treatment in childhood asthma (change in cough/wheeze)	31557306
	Systemic lupus erythematosus	24871463
	Visceral adipose tissue/subcutaneous adipose tissue ratio	22589738

**Table 2:** Genes encoding the contactin family and the regions studied.

**Gene**	**Chr**	**Start**	**End**	**−400 kb**	**+400 kb**
*CNTN1*	12	40,692,442	41,072,412	40,292,442	41,472,412
*CNTN2*	1	205,042,937	205,078,272	204,642,937	205,478,272
*CNTN3*	3	74,262,568	74,521,140	73,862,568	74,921,140
*CNTN4*	3	2,098,803	3,057,961	1,698,803	3,457,961
*CNTN5*	11	99,021,190	100,358,885	98,621,190	100,758,885
*CNTN6*	3	1,092,661	1,403,594	692,661	1,803,594

Where: Positions refer to build 37.

**Table 3:** UK Biobank cohort description.

	**Men**	**Women**	**All**
N (% men)	185,228	217,635	402,863 (46.0)
Age (years)	57.1 (8.1)	56.7 (7.9)	56.9 (8.0)
WHR	0.94 (0.06)	0.82 (0.07)	0.87 (0.09)
BMI (kg/m^2^)	27.8 (4.2)	27.0 (5.1)	27.4 (4.8)
SBP (mmHg)	141 (17)	136 (19)	138 (19)
DBP (mmHg)	84 (10)	81 (10)	82 (10)
SBP * (mmHg)	145 (19)	138 (21)	141 (21)
DBP * (mmHg)	87 (11)	82 (11)	84 (11)
HbA1C (mmol/mol)	36.3 (7.3)	35.7 (5.7)	36.0 (6.5)
Neuroticism score	3.6 (3.2)	4.6 (3.2)	4.1 (3.3)
CVD	5886 (3.2)	2294 (1.1)	8180 (2.03)
T2D	11,189 (6.0)	6232 (2.9)	17421 (4.3)
BD	875 (1.5)	999 (1.4)	1874 (1.5)
GAD	3069 (7.2)	6029 (12.8)	9098 (10.1)
MDD	9684 (19.8)	21,199 (35.0)	30,883 (28.2)
Addiction	3895 (6.9)	3580 (4.9)	7475 (5.8)
Mood instability	77,173 (41.7)	100,841 (46.3)	178,014 (44.2)
Risk-taking	18,519 (33.2)	13,702 (19.4)	32,221 (25.5)
Current Smoking	21,821 (11.8)	18,803 (8.7)	40,624 (10.1)

Where *, adjusted to reflect treatment-naïve levels; CVD, cardiovascular disease; BD, bipolar disorder; GAD, generalised anxiety disorder; MDD, major depressive disorder. Continuous variables are presented as means (standard deviation), binary variables are presented as N (%).

**Table 4:** Lead SNP by gene and phenotype.

**Gene**	**Trait**	**Lead SNP**	**A1**	**A1F**	**N**	**β/OR**	**SE**	**P**
*CNTN1*	Smoking	rs11174809	T	0.35	398,675	1.025	0.005	9.52 × 10^−7^
*CNTN2*	Risks	rs35068223	T	0.19	377,622	1.036	0.007	1.77 × 10^−7^
*CNTN2*	WHRadjBMI	rs6593925	G	0.09	401,149	−0.001	0.000	2.38 × 10^−11^
*CNTN2*	WHRadjBMI *	rs11240349	A	0.46	400,770	−0.001	0.000	3.98 × 10^−8^
*CNTN4*	BMI	rs3856837	C	0.48	394,050	−0.054	0.011	4.28 × 10^−7^
*CNTN4*	BMI *	rs4685542	C	0.14	391,199	−0.075	0.016	1.66 × 10^−6^
*CNTN4*	Risks	rs62232818	T	0.10	383,065	1.042	0.009	3.09 × 10^−6^
*CNTN5*	Neuroticism	rs10790767	T	0.40	324,775	0.042	0.008	2.43 × 10^−7^
*CNTN5*	HbA1C	rs11606890	C	0.10	384,094	−0.007	0.001	1.14 × 10^−37^
*CNTN5*	DBP	rs633185	G	0.29	370,841	−0.459	0.028	1.94 × 10^−59^
*CNTN5*	SBP	rs633185	G	0.29	370,770	−0.787	0.049	2.85 × 10^−58^

WhereA1, effect allele; A1F, effect allele frequency; risks, risk-taking behaviour, 1:204945338 = rs35068223; *, conditional analysis.

## Supplementary Materials

The following are available online at https://www.mdpi.com/2073-4425/11/11/1326/s1, Figure S1: Linkage disequilibrium within the contactin genes: (A) *CNTN1*, (B) *CNTN2*, (C) *CNTN4*, (D) *CNTN5*. Red dots indicate lead SNPs. Table S1: Genetic variants in *CNTN1* significantly associated with current smoking. Table S2: Genetic variants in *CNTN2* significantly associated with WHRadjBMI or risk-taking. Table S3: Genetic variants in *CNTN4* with significant associations with BMI or risk-taking. Table S4: Genetic variants in *CNTN5* significantly associated with neuroticism, systolic and diastolic blood pressure, HbA1c. Table S5: Previously reported associations with the CNTN family of genes. Table S6: Predicted effects of SNPs significantly associated with one or more trait. Table S7: Genetic variants in *CNTN1 CNTN2, CNTN4* and *CNTN5* with genotype-specific effects on gene expression. Table S8: Genotype-specific expression of *ARHGAP42* and *TMEM133*.
