# Supplementary material for: Exploring the Role of Contactins across Psychological, Psychiatric and Cardiometabolic Traits within UK Biobank
Source: Genes (Basel). 2020 Nov 10;11(11):1326. doi: 10.3390/genes11111326 (PMC7697406; doi:10.3390/genes11111326)
Supplement: Supplementary file 1 [file genes-11-01326-s001.pdf]

Supplementary Figure 1: Linkage disequilibrium within the contactin genes: A) *CNTN1*, B) *CNTN2*, C) *CNTN4*, D) *CNTN5*. Red dots indicate lead SNPs.

A)

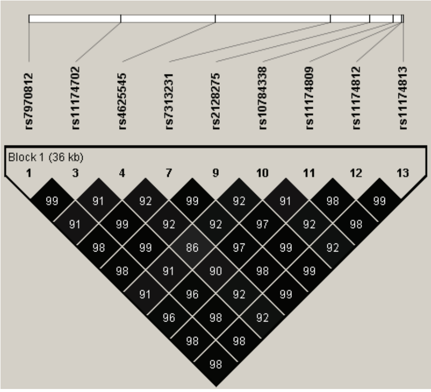
rs7970812
rs11174702
rs4625545
rs7313231
rs2128275
rs10784338
rs11174809
rs11174812
rs11174813
Block 1 (36 kb)
1 3 4 7 9 10 11 12 13

B)

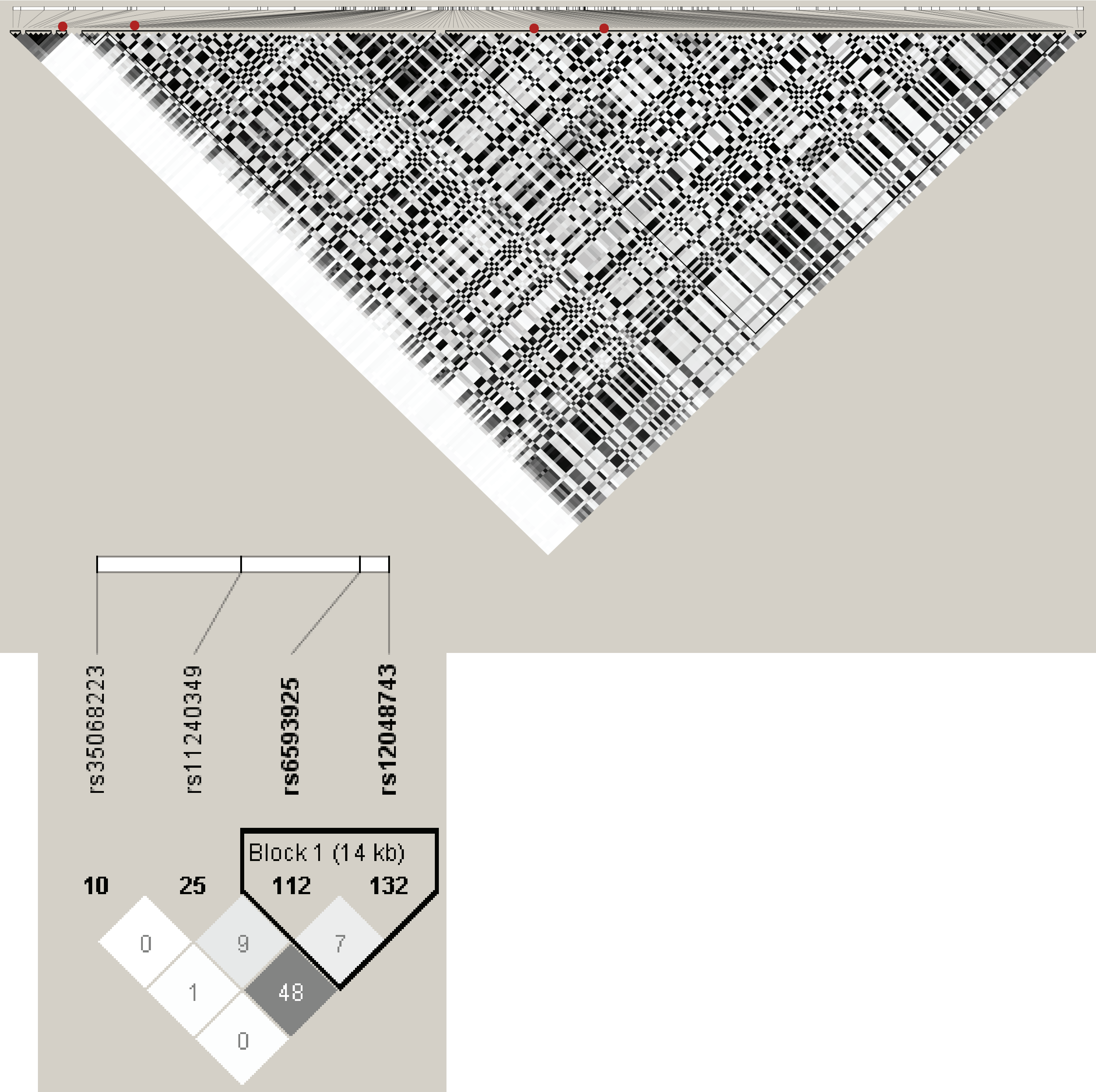
rs35068223
rs11240349
rs6593925
rs12048743
Block 1 (14 kb)
10
25
112
132
0
9
7
1
48
0

C)

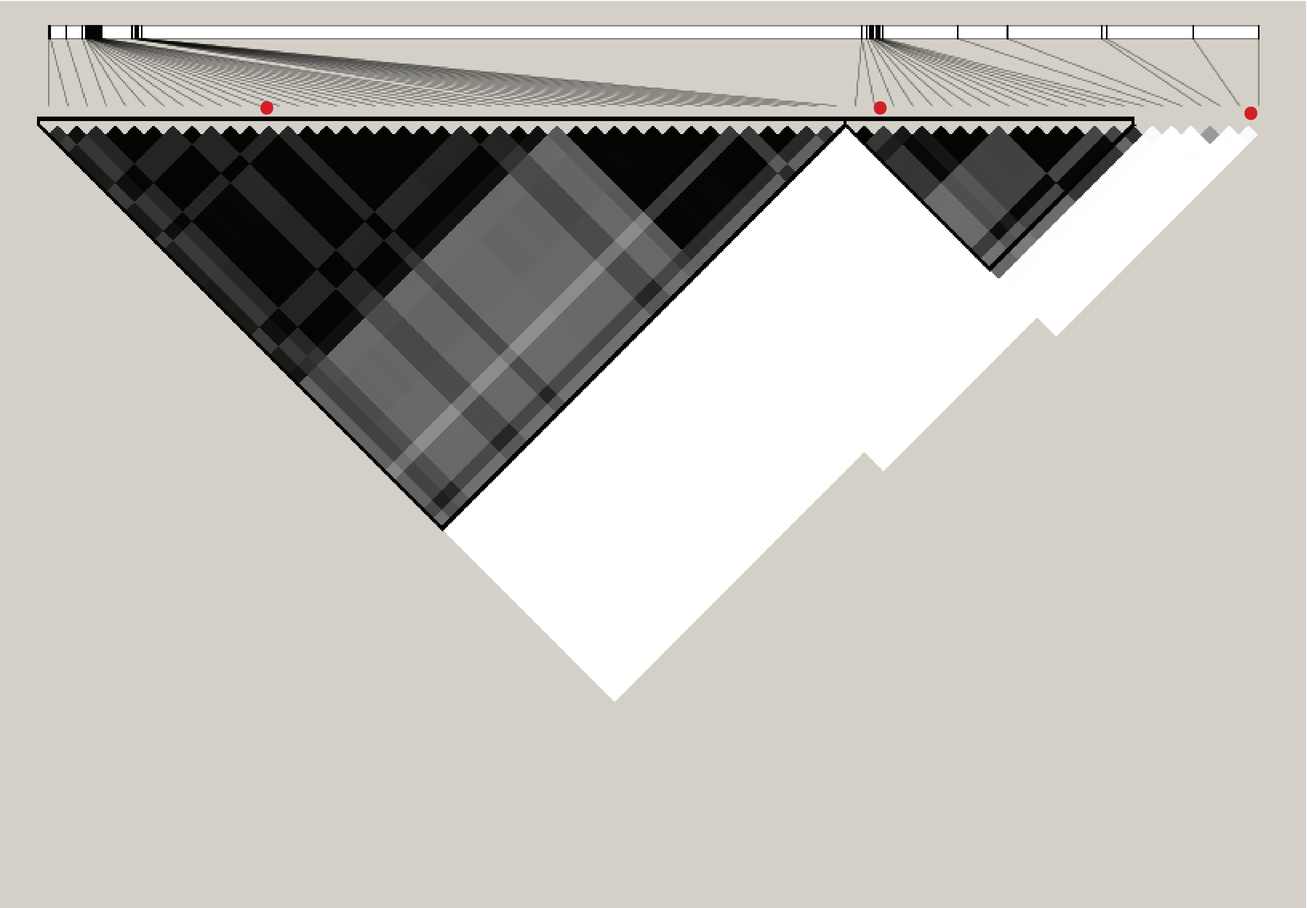

D)

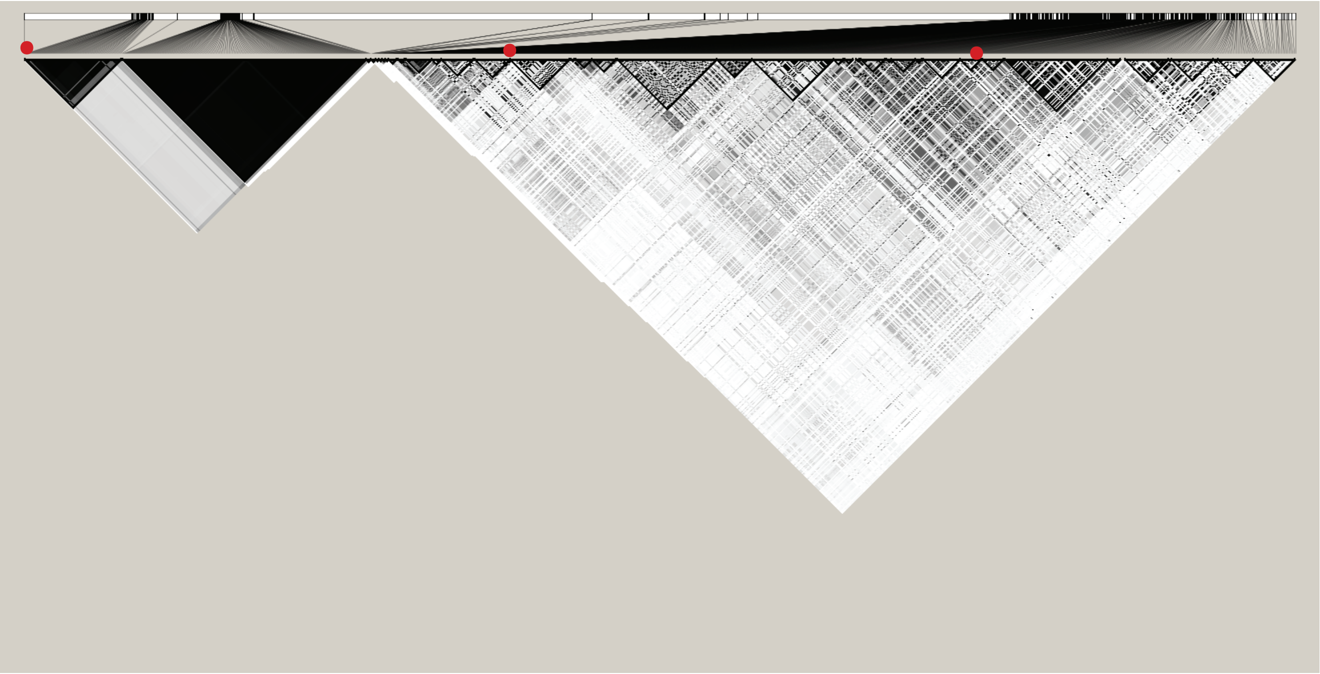

STable 1: Genetic variants in *CNTN1* with significant or suggestive associations with current smoking

SNP	A1	A1F	NMISS	OR	SE	P
rs7970812	C	0.35	397292	1.02	0.00	**2.17E-06**
rs11174702	C	0.35	397700	1.02	0.00	**1.53E-06**
rs4625545	A	0.34	400251	1.02	0.01	**2.91E-06**
rs7313231	T	0.35	401748	1.02	0.00	**1.78E-06**
rs2128275	C	0.35	401648	1.02	0.00	**1.38E-06**
rs10784338	T	0.37	400799	1.02	0.00	**4.92E-06**
rs11174809	T	0.35	398675	1.03	0.00	**9.52E-07**
rs11174812	T	0.35	400597	1.02	0.00	**1.09E-06**
rs11174813	C	0.35	400660	1.02	0.00	**1.05E-06**

STable 2: Genetic variants in *CNTN2* with significant or suggestive associations with risk-taking or WHRadjBMI

SNP	A1	A1F	NMISS	OR	SE	P_risks	NMISS	Beta	SE	P_whradjBMI	BETA	SE	P_whradjBMI*
rs6593925	G	0.09	389508	0.977	0.009	0.0106	**401149**	**-0.001**	**0.000**	**2.38E-11**	NA	NA	NA
rs12078075	G	0.08	388553	0.975	0.009	0.0067	**400183**	**-0.001**	**0.000**	**2.63E-11**	-0.001	0.001	0.5651
rs2369633	T	0.09	389301	0.978	0.009	0.0138	**400947**	**-0.001**	**0.000**	**3.13E-11**	NA	NA	NA
rs3851294	A	0.09	389799	0.977	0.009	0.0130	**401453**	**-0.001**	**0.000**	**4.07E-11**	NA	NA	NA
rs896319	G	0.08	388706	0.976	0.009	0.0096	**400332**	**-0.001**	**0.000**	**1.14E-10**	0.000	0.001	0.8618
rs12048743	G	0.44	388884	1.021	0.005	0.0001	**400504**	**0.001**	**0.000**	**2.86E-10**	**0.001**	**0.000**	**2.90E-06**
rs4951163	T	0.08	388439	0.975	0.010	0.0082	**400049**	**-0.001**	**0.000**	**6.28E-10**	0.000	0.001	0.915
rs1572993	A	0.43	388684	1.018	0.005	0.0008	**400308**	**0.001**	**0.000**	**7.14E-10**	**0.001**	**0.000**	**4.61E-06**
rs10900446	A	0.39	389019	1.020	0.005	0.0003	**400648**	**0.001**	**0.000**	**7.40E-10**	**0.001**	**0.000**	**3.16E-06**
rs9659978	A	0.09	389368	0.978	0.009	0.0147	**401012**	**-0.001**	**0.000**	**1.09E-09**	0.002	0.001	0.1169
rs17728	T	0.09	389359	0.978	0.009	0.0149	**401002**	**-0.001**	**0.000**	**1.09E-09**	0.002	0.001	0.1183
rs11240358	A	0.40	389040	1.020	0.005	0.0003	**400676**	**0.001**	**0.000**	**1.30E-09**	**0.001**	**0.000**	**5.60E-06**
rs10900448	A	0.40	388909	1.020	0.005	0.0002	**400543**	**0.001**	**0.000**	**1.43E-09**	**0.001**	**0.000**	**6.16E-06**
rs1172134	A	0.09	388761	0.976	0.009	0.0097	**400386**	**-0.001**	**0.000**	**1.60E-09**	0.002	0.001	0.03506
rs1172128	A	0.08	389799	0.974	0.010	0.0059	**401453**	**-0.001**	**0.000**	**2.61E-09**	0.001	0.001	0.4477
rs11240351	G	0.41	388380	1.016	0.005	0.0025	**399990**	**0.001**	**0.000**	**4.21E-09**	0.001	0.000	1.60E-05
rs4951159	G	0.08	381034	0.986	0.010	0.1405	**392378**	**-0.001**	**0.000**	**2.53E-08**	0.000	0.000	0.9121
1:205163688	TA	0.12	389616	0.982	0.008	0.0268	**401266**	**-0.001**	**0.000**	**2.96E-08**	0.000	0.000	0.8558
rs2369645	A	0.13	389646	0.982	0.008	0.0241	**401299**	**-0.001**	**0.000**	**3.01E-08**	0.000	0.000	0.8966
rs11240368	T	0.13	389639	0.982	0.008	0.0248	**401292**	**-0.001**	**0.000**	**3.09E-08**	0.000	0.000	0.8833
rs34797971	A	0.12	386850	0.985	0.008	0.0575	**398421**	**-0.001**	**0.000**	**3.27E-08**	0.000	0.000	0.7747
rs3902193	C	0.13	389638	0.982	0.008	0.0260	**401291**	**-0.001**	**0.000**	**3.40E-08**	0.000	0.000	0.8579
rs10900449	T	0.13	389637	0.982	0.008	0.0259	**401291**	**-0.001**	**0.000**	**3.42E-08**	0.000	0.000	0.8469
rs10900451	A	0.13	389621	0.982	0.008	0.0252	**401274**	**-0.001**	**0.000**	**3.58E-08**	0.000	0.000	0.8514
rs386638726	G	0.12	389394	0.982	0.008	0.0277	**401043**	**-0.001**	**0.000**	**3.62E-08**	0.000	0.000	0.8584
rs6703574	A	0.13	389758	0.982	0.008	0.0249	**401413**	**-0.001**	**0.000**	**3.67E-08**	0.000	0.000	0.8658
1:205055361	ATT	0.41	293841	0.983	0.006	0.0057	**302671**	**-0.001**	**0.000**	**3.81E-08**	-0.001	0.000	2.17E-03
rs12728202	T	0.12	389694	0.982	0.008	0.0235	**401347**	**-0.001**	**0.000**	**3.95E-08**	0.000	0.000	0.812

rs7536494	C	0.13	389721	0.982	0.008	0.0281	**401376**	**-0.001**	**0.000**	**3.97E-08**	0.000	0.000	0.8297
rs2095854	G	0.13	389661	0.982	0.008	0.0253	**401315**	**-0.001**	**0.000**	**4.13E-08**	0.000	0.000	0.7953
rs3851292	A	0.13	389624	0.982	0.008	0.0228	**401276**	**-0.001**	**0.000**	**4.31E-08**	0.000	0.000	0.7979
rs3851286	C	0.12	389636	0.982	0.008	0.0287	**401286**	**-0.001**	**0.000**	**4.38E-08**	0.000	0.000	0.7706
rs10900466	C	0.13	389778	0.982	0.008	0.0264	**401431**	**-0.001**	**0.000**	**4.46E-08**	0.000	0.000	0.8216
rs6700182	A	0.13	389659	0.982	0.008	0.0269	**401311**	**-0.001**	**0.000**	**4.46E-08**	0.000	0.000	0.777
rs3851291	T	0.13	389621	0.982	0.008	0.0240	**401273**	**-0.001**	**0.000**	**4.57E-08**	0.000	0.000	0.7725
rs10900458	C	0.12	389706	0.982	0.008	0.0287	**401360**	**-0.001**	**0.000**	**4.67E-08**	0.000	0.000	0.7497
rs6593929	A	0.13	389780	0.982	0.008	0.0281	**401434**	**-0.001**	**0.000**	**4.73E-08**	0.000	0.000	0.8039
rs11240374	T	0.13	389782	0.982	0.008	0.0278	**401436**	**-0.001**	**0.000**	**4.77E-08**	0.000	0.000	0.8092
rs12023022	A	0.12	389691	0.982	0.008	0.0282	**401345**	**-0.001**	**0.000**	**4.78E-08**	0.000	0.000	0.7349
rs11240371	C	0.13	389310	0.982	0.008	0.0254	**400954**	**-0.001**	**0.000**	**4.81E-08**	0.000	0.000	0.8207
rs4950982	T	0.13	389581	0.983	0.008	0.0306	**401228**	**-0.001**	**0.000**	**4.88E-08**	0.000	0.000	0.7765
rs12137432	A	0.13	389419	0.983	0.008	0.0320	**401065**	**-0.001**	**0.000**	**4.97E-08**	0.000	0.000	0.8194
rs6684220	A	0.12	389430	0.982	0.008	0.0280	**401074**	**-0.001**	**0.000**	**5.12E-08**	0.000	0.000	0.7597
rs2369634	G	0.12	389713	0.982	0.008	0.0278	**401368**	**-0.001**	**0.000**	**5.72E-08**	0.000	0.000	0.7109
rs2887527	A	0.13	389799	0.982	0.008	0.0281	**401453**	**-0.001**	**0.000**	**5.89E-08**	0.000	0.000	0.7575
rs896322	A	0.12	389295	0.983	0.008	0.0354	**400939**	**-0.001**	**0.000**	**5.91E-08**	0.000	0.000	0.7201
rs10793727	A	0.12	389317	0.983	0.008	0.0342	**400962**	**-0.001**	**0.000**	**6.69E-08**	0.000	0.000	0.7021
rs1172141	C	0.13	389327	0.983	0.008	0.0366	**400971**	**-0.001**	**0.000**	**6.73E-08**	0.000	0.000	0.7493
rs11240396	T	0.13	389341	0.983	0.008	0.0361	**400985**	**-0.001**	**0.000**	**7.31E-08**	0.000	0.000	0.7431
rs1172155	T	0.13	389109	0.982	0.008	0.0266	**400751**	**-0.001**	**0.000**	**9.45E-08**	0.000	0.000	0.7243
rs55966786	A	0.07	389110	0.984	0.010	0.1146	**400748**	**-0.001**	**0.000**	**1.76E-07**	0.000	0.000	0.9542
rs7541409	T	0.12	389292	0.983	0.008	0.0312	**400932**	**-0.001**	**0.000**	**1.91E-07**	0.000	0.000	0.606
rs11240365	C	0.13	389584	0.983	0.008	0.0312	**401236**	**-0.001**	**0.000**	**1.94E-07**	0.000	0.000	0.6108
rs10900443	G	0.13	389494	0.985	0.008	0.0647	**401131**	**-0.001**	**0.000**	**2.12E-07**	0.000	0.000	0.6136
rs10900444	C	0.12	389400	0.986	0.008	0.0706	**401035**	**-0.001**	**0.000**	**2.12E-07**	0.000	0.000	0.5491
rs202021413	T	0.49	313732	1.014	0.006	0.0177	**323164**	**0.001**	**0.000**	**2.17E-07**	0.001	0.000	5.16E-04
rs61060413	A	0.13	389693	0.985	0.008	0.0581	**401343**	**-0.001**	**0.000**	**2.20E-07**	0.000	0.000	0.6143
rs4330974	T	0.13	389554	0.983	0.008	0.0331	**401207**	**-0.001**	**0.000**	**2.37E-07**	0.000	0.000	0.563
rs7518906	G	0.13	389594	0.985	0.008	0.0627	**401237**	**-0.001**	**0.000**	**2.37E-07**	0.000	0.000	0.5925

rs1817001	T	0.13	389548	0.984	0.008	0.0442	**401196**	**-0.001**	**0.000**	**2.55E-07**	0.000	0.000	0.5592
rs2197909	T	0.13	389656	0.985	0.008	0.0485	**401308**	**-0.001**	**0.000**	**2.65E-07**	0.000	0.000	0.5983
rs4950979	G	0.14	389799	0.984	0.008	0.0432	**401453**	**-0.001**	**0.000**	**2.66E-07**	0.000	0.000	0.7861
rs7536902	A	0.14	389659	0.984	0.008	0.0377	**401306**	**-0.001**	**0.000**	**2.86E-07**	0.000	0.000	0.7282
rs372105635	C	0.14	387455	0.984	0.008	0.0366	**399021**	**-0.001**	**0.000**	**2.91E-07**	0.000	0.000	0.6576
rs4951168	C	0.14	389799	0.985	0.008	0.0573	**401453**	**-0.001**	**0.000**	**3.05E-07**	0.000	0.000	0.5615
rs7523477	T	0.14	389742	0.985	0.008	0.0589	**401393**	**-0.001**	**0.000**	**3.07E-07**	0.000	0.000	0.5811
rs4509642	C	0.14	389799	0.984	0.008	0.0355	**401453**	**-0.001**	**0.000**	**3.14E-07**	0.000	0.000	0.5234
rs9651227	A	0.13	389667	0.985	0.008	0.0611	**401319**	**-0.001**	**0.000**	**3.16E-07**	0.000	0.000	0.5336
rs373079331	CA	0.11	374151	0.979	0.009	0.0141	**385364**	**-0.001**	**0.000**	**3.19E-07**	0.001	0.000	0.1792
rs4950981	G	0.13	389677	0.984	0.008	0.0373	**401326**	**-0.001**	**0.000**	**3.27E-07**	0.000	0.000	0.5022
rs4951166	A	0.12	389006	0.985	0.008	0.0572	**400623**	**-0.001**	**0.000**	**3.42E-07**	0.000	0.000	0.5017
rs12409915	T	0.14	389732	0.985	0.008	0.0523	**401382**	**-0.001**	**0.000**	**3.43E-07**	0.000	0.000	0.5189
rs7537322	G	0.14	389736	0.985	0.008	0.0523	**401387**	**-0.001**	**0.000**	**3.46E-07**	0.000	0.000	0.5184
rs10664521	A	0.14	388868	0.983	0.008	0.0314	**400490**	**-0.001**	**0.000**	**3.54E-07**	0.000	0.000	0.5616
rs2185686	C	0.13	389672	0.984	0.008	0.0384	**401321**	**-0.001**	**0.000**	**3.54E-07**	0.000	0.000	0.4893
rs10736840	A	0.14	389469	0.984	0.008	0.0334	**401112**	**-0.001**	**0.000**	**3.56E-07**	0.000	0.000	0.6789
rs74848951	T	0.14	389735	0.985	0.008	0.0513	**401387**	**-0.001**	**0.000**	**3.56E-07**	0.000	0.000	0.513
1:205064498	GT	0.13	389368	0.985	0.008	0.0517	**401011**	**-0.001**	**0.000**	**3.86E-07**	0.000	0.000	0.4682
rs3820338	A	0.14	389166	0.984	0.008	0.0408	**400785**	**-0.001**	**0.000**	**3.94E-07**	0.000	0.000	0.5558
rs6683762	T	0.13	389553	0.984	0.008	0.0399	**401196**	**-0.001**	**0.000**	**4.36E-07**	0.000	0.000	0.4547
rs3753855	A	0.14	389253	0.984	0.008	0.0390	**400875**	**-0.001**	**0.000**	**4.59E-07**	0.000	0.000	0.5309
rs56323810	A	0.07	389799	0.985	0.010	0.1395	**401453**	**-0.001**	**0.000**	**4.68E-07**	0.000	0.000	0.8325
1:205099997	CT	0.12	388275	0.983	0.008	0.0308	**399881**	**-0.001**	**0.000**	**5.43E-07**	0.000	0.000	0.3605
rs2211330	T	0.22	367057	1.001	0.007	0.8261	**378006**	**-0.001**	**0.000**	**5.97E-07**	0.000	0.000	0.02399
rs10157145	T	0.49	380752	0.982	0.005	0.0009	**392177**	**-0.001**	**0.000**	**6.88E-07**	0.000	0.000	1.76E-03
rs1668874	A	0.12	389049	0.983	0.008	0.0330	**400689**	**-0.001**	**0.000**	**7.85E-07**	0.000	0.000	0.3184
rs1062715	A	0.24	389405	1.007	0.006	0.2977	**401048**	**0.001**	**0.000**	**1.27E-06**	0.001	0.000	1.67E-04
rs61822628	C	0.25	389439	1.007	0.006	0.2970	**401086**	**0.001**	**0.000**	**1.45E-06**	0.001	0.000	1.91E-04
rs3183297	G	0.24	389307	1.006	0.006	0.3108	**400960**	**0.001**	**0.000**	**1.48E-06**	0.001	0.000	1.95E-04
rs6703220	T	0.47	384680	1.019	0.005	0.0006	**396193**	**0.001**	**0.000**	**1.49E-06**	0.000	0.000	1.64E-03

rs7538328	C	0.24	389713	1.007	0.006	0.2700	**401366**	**0.001**	**0.000**	**1.50E-06**	0.001	0.000	1.96E-04
rs61822627	A	0.24	389628	1.006	0.006	0.3167	**401278**	**0.001**	**0.000**	**1.53E-06**	0.001	0.000	1.97E-04
rs11804620	A	0.24	389136	1.006	0.006	0.3393	**400775**	**0.001**	**0.000**	**1.63E-06**	0.001	0.000	1.93E-04
rs3795556	C	0.25	389351	1.006	0.006	0.3191	**401003**	**0.001**	**0.000**	**1.72E-06**	0.001	0.000	2.23E-04
rs1339321	A	0.21	389312	1.006	0.007	0.3231	**400958**	**0.001**	**0.000**	**1.72E-06**	0.001	0.000	1.58E-04
rs16855154	C	0.25	389313	1.006	0.006	0.3323	**400965**	**0.001**	**0.000**	**1.83E-06**	0.001	0.000	2.35E-04
rs11811115	C	0.24	389109	1.006	0.006	0.3488	**400746**	**0.001**	**0.000**	**1.84E-06**	0.001	0.000	2.13E-04
rs12048416	C	0.24	389112	1.006	0.006	0.3588	**400748**	**0.001**	**0.000**	**1.89E-06**	0.001	0.000	2.21E-04
rs12048453	C	0.25	389202	1.006	0.006	0.3779	**400846**	**0.001**	**0.000**	**1.92E-06**	0.001	0.000	2.35E-04
rs3753847	T	0.15	381967	0.987	0.008	0.0796	**393343**	**-0.001**	**0.000**	**1.99E-06**	0.000	0.000	0.7856
rs10494860	G	0.25	389618	1.006	0.006	0.2987	**401265**	**0.001**	**0.000**	**2.07E-06**	0.001	0.000	2.68E-04
rs6593930	T	0.25	389729	1.006	0.006	0.3051	**401381**	**0.001**	**0.000**	**2.20E-06**	0.001	0.000	2.72E-04
rs6687271	C	0.25	389663	1.006	0.006	0.3042	**401313**	**0.001**	**0.000**	**2.21E-06**	0.001	0.000	2.78E-04
rs16855186	C	0.21	389460	1.006	0.007	0.3951	**401110**	**0.001**	**0.000**	**2.21E-06**	0.001	0.000	1.97E-04
rs3903399	C	0.21	388557	1.007	0.007	0.3145	**400180**	**0.001**	**0.000**	**2.23E-06**	0.001	0.000	2.01E-04
rs4950984	G	0.22	388271	1.005	0.007	0.4761	**399877**	**0.001**	**0.000**	**2.30E-06**	0.001	0.000	2.04E-04
rs1061132	A	0.24	389233	1.006	0.006	0.3070	**400884**	**0.001**	**0.000**	**2.40E-06**	0.001	0.000	2.70E-04
rs1572995	C	0.13	389069	0.988	0.008	0.1167	**400695**	**-0.001**	**0.000**	**2.72E-06**	0.000	0.000	0.5805
rs7529665	A	0.24	389194	1.006	0.006	0.3397	**400834**	**0.001**	**0.000**	**2.86E-06**	0.001	0.000	3.09E-04
rs12039805	A	0.24	389331	1.007	0.006	0.2914	**400987**	**0.001**	**0.000**	**2.94E-06**	0.001	0.000	3.11E-04
rs3862948	A	0.23	389201	1.007	0.006	0.2484	**400849**	**0.001**	**0.000**	**2.95E-06**	0.001	0.000	3.09E-04
rs6670870	T	0.25	389270	1.007	0.006	0.2638	**400908**	**0.001**	**0.000**	**2.97E-06**	0.001	0.000	3.37E-04
rs12033493	T	0.19	388977	1.006	0.007	0.4093	**400614**	**0.001**	**0.000**	**2.99E-06**	0.001	0.000	2.01E-04
rs61822621	A	0.24	389210	1.006	0.006	0.3076	**400858**	**0.001**	**0.000**	**3.01E-06**	0.001	0.000	3.18E-04
1:205098289	GT	0.18	305857	0.986	0.008	0.0733	**314923**	**-0.001**	**0.000**	**3.06E-06**	0.000	0.000	0.8576
rs7530217	A	0.24	389219	1.007	0.006	0.2874	**400871**	**0.001**	**0.000**	**3.17E-06**	0.001	0.000	3.37E-04
rs74787661	A	0.19	388952	1.006	0.007	0.4116	**400589**	**0.001**	**0.000**	**3.21E-06**	0.001	0.000	2.12E-04
rs896321	G	0.12	389799	0.981	0.008	0.0194	**401453**	**-0.001**	**0.000**	**3.63E-06**	0.000	0.000	0.1939
rs6664360	T	0.24	389535	1.006	0.006	0.3774	**401187**	**0.001**	**0.000**	**3.89E-06**	0.001	0.000	0.00041
rs754413251	G	0.20	388501	1.006	0.007	0.3879	**400118**	**0.001**	**0.000**	**3.91E-06**	0.001	0.000	2.89E-04
rs10793726	G	0.15	388141	0.987	0.007	0.0844	**399737**	**-0.001**	**0.000**	**3.98E-06**	0.000	0.000	0.4851

rs11808879	A	0.24	388798	1.006	0.006	0.3360	**400438**	**0.001**	**0.000**	**4.00E-06**	0.001	0.000	4.07E-04
rs61822618	T	0.20	389171	1.006	0.007	0.3600	**400814**	**0.001**	**0.000**	**4.24E-06**	0.001	0.000	3.05E-04
rs12025836	C	0.20	389169	1.006	0.007	0.3637	**400810**	**0.001**	**0.000**	**4.32E-06**	0.001	0.000	3.06E-04
rs4951182	C	0.45	387272	1.017	0.005	0.0016	**398859**	**0.001**	**0.000**	**4.48E-06**	0.000	0.000	4.19E-03
rs12027259	C	0.20	389022	1.006	0.007	0.3811	**400659**	**0.001**	**0.000**	**4.49E-06**	0.001	0.000	3.17E-04
rs7514499	C	0.20	389022	1.006	0.007	0.3766	**400659**	**0.001**	**0.000**	**4.50E-06**	0.001	0.000	3.18E-04
rs7513016	A	0.10	377926	0.992	0.009	0.3892	**389175**	**-0.001**	**0.000**	**4.50E-06**	0.000	0.000	0.9361
rs12022384	C	0.20	389068	1.006	0.007	0.3717	**400705**	**0.001**	**0.000**	**4.73E-06**	0.001	0.000	3.33E-04
rs1172122	C	0.12	389585	0.982	0.008	0.0262	**401235**	**-0.001**	**0.000**	**4.88E-06**	0.000	0.000	0.1821
rs1172123	G	0.12	389581	0.982	0.008	0.0273	**401231**	**-0.001**	**0.000**	**4.91E-06**	0.000	0.000	0.1776
rs3851287	T	0.21	389680	1.005	0.006	0.4330	**401331**	**0.001**	**0.000**	**5.50E-06**	0.001	0.000	4.38E-04
1:205198281	TTGTG	0.14	381520	0.989	0.008	0.1708	**392922**	**-0.001**	**0.000**	**6.18E-06**	0.000	0.000	0.322
rs3927069	G	0.21	389622	1.005	0.006	0.4219	401271	0.001	0.000	0.0000	0.001	0.000	5.01E-04
rs12042196	T	0.21	389543	1.005	0.006	0.4005	401191	0.001	0.000	0.0000	0.001	0.000	5.98E-04
1:205188495	CT	0.06	345565	0.974	0.012	0.0295	355896	-0.001	0.000	0.0000	0.000	0.000	0.4309
rs16855045	C	0.20	388876	1.004	0.007	0.5328	400514	0.001	0.000	0.0000	0.001	0.000	5.12E-04
rs61823969	G	0.21	389475	1.005	0.006	0.4058	401122	0.001	0.000	0.0000	0.001	0.000	6.47E-04
rs28447128	C	0.20	388759	1.004	0.007	0.5563	400391	0.001	0.000	0.0000	0.001	0.000	5.21E-04
rs16855051	G	0.20	388887	1.004	0.007	0.5158	400526	0.001	0.000	0.0000	0.001	0.000	5.44E-04
rs16855053	G	0.20	388890	1.004	0.007	0.5142	400529	0.001	0.000	0.0000	0.001	0.000	5.46E-04
rs56283998	C	0.20	388305	1.004	0.007	0.5535	399916	0.001	0.000	0.0000	0.001	0.000	5.29E-04
rs1002926	T	0.20	388674	1.004	0.007	0.5338	400305	0.001	0.000	0.0000	0.001	0.000	5.61E-04
rs2071533	T	0.12	378315	0.990	0.008	0.2073	389576	-0.001	0.000	0.0000	0.000	0.000	0.7654
1:205038158	T	0.33	301890	0.994	0.006	0.3673	310928	-0.001	0.000	0.0001	**-0.001**	**0.000**	**5.57E-08**
rs11240349	A	0.46	389137	0.989	0.005	0.0305	400770	0.000	0.000	0.0014	**-0.001**	**0.000**	**3.98E-08**
rs6662930	C	0.46	388713	0.989	0.005	0.0307	400342	0.000	0.000	0.0015	**-0.001**	**0.000**	**5.14E-08**
rs12134724	C	0.46	387842	0.989	0.005	0.0414	399432	0.000	0.000	0.0016	**-0.001**	**0.000**	**5.03E-08**
rs1042831	C	0.46	388662	0.989	0.005	0.0292	400286	0.000	0.000	0.0017	**-0.001**	**0.000**	**5.57E-08**
rs6696846	T	0.47	388773	0.990	0.005	0.0622	400403	0.000	0.000	0.0051	**-0.001**	**0.000**	**2.81E-07**
rs112673145	TA	0.43	382003	0.991	0.005	0.0782	393445	0.000	0.000	0.0055	**-0.001**	**0.000**	**9.22E-07**
rs12142402	T	0.43	387421	0.988	0.005	0.0257	398995	0.000	0.000	0.0062	**-0.001**	**0.000**	**1.14E-06**

rs11240370	G	0.43	387906	0.987	0.005	0.0154	399505	0.000	0.000	0.0066	**-0.001**	**0.000**	**1.22E-06**
rs11240355	A	0.43	389167	0.988	0.005	0.0217	400803	0.000	0.000	0.0069	**-0.001**	**0.000**	**1.11E-06**
rs12117194	C	0.49	389799	1.010	0.005	0.0598	401453	0.000	0.000	0.0069	**0.001**	**0.000**	**2.31E-07**
rs11576867	A	0.43	389188	0.988	0.005	0.0216	400826	0.000	0.000	0.0069	**-0.001**	**0.000**	**1.14E-06**
rs61822565	C	0.43	389095	0.988	0.005	0.0218	400730	0.000	0.000	0.0071	**-0.001**	**0.000**	**1.29E-06**
rs6593922	T	0.43	389117	0.988	0.005	0.0207	400753	0.000	0.000	0.0071	**-0.001**	**0.000**	**1.30E-06**
rs3738155	T	0.43	389145	0.988	0.005	0.0211	400781	0.000	0.000	0.0071	**-0.001**	**0.000**	**1.30E-06**
rs12125465	A	0.43	389144	0.988	0.005	0.0213	400780	0.000	0.000	0.0071	**-0.001**	**0.000**	**1.32E-06**
rs11577406	T	0.43	389152	0.988	0.005	0.0221	400789	0.000	0.000	0.0071	**-0.001**	**0.000**	**1.16E-06**
rs61822568	G	0.43	389165	0.988	0.005	0.0218	400802	0.000	0.000	0.0072	**-0.001**	**0.000**	**1.33E-06**
rs11240360	C	0.43	389238	0.988	0.005	0.0212	400879	0.000	0.000	0.0072	**-0.001**	**0.000**	**1.33E-06**
rs61822567	C	0.43	389045	0.988	0.005	0.0230	400679	0.000	0.000	0.0072	**-0.001**	**0.000**	**1.35E-06**
rs17344537	G	0.43	389799	0.987	0.005	0.0178	401453	0.000	0.000	0.0072	**-0.001**	**0.000**	**1.36E-06**
rs61822619	G	0.43	389728	0.988	0.005	0.0191	401379	0.000	0.000	0.0072	**-0.001**	**0.000**	**1.35E-06**
rs72757524	T	0.43	389248	0.988	0.005	0.0230	400893	0.000	0.000	0.0072	**-0.001**	**0.000**	**1.35E-06**
rs12239712	T	0.43	389222	0.988	0.005	0.0217	400864	0.000	0.000	0.0072	**-0.001**	**0.000**	**1.34E-06**
rs72757512	A	0.43	389153	0.988	0.005	0.0218	400790	0.000	0.000	0.0073	**-0.001**	**0.000**	**1.37E-06**
rs112653854	T	0.43	389001	0.988	0.005	0.0222	400635	0.000	0.000	0.0075	**-0.001**	**0.000**	**1.39E-06**
rs759669171	A	0.43	389041	0.988	0.005	0.0196	400670	0.000	0.000	0.0077	**-0.001**	**0.000**	**1.57E-06**
rs10158848	T	0.44	388939	0.989	0.005	0.0321	400570	0.000	0.000	0.0078	**-0.001**	**0.000**	**1.40E-06**
rs4394715	G	0.43	389016	0.987	0.005	0.0139	400639	0.000	0.000	0.0080	**-0.001**	**0.000**	**1.65E-06**
rs112267268	C	0.43	388799	0.988	0.005	0.0262	400432	0.000	0.000	0.0080	**-0.001**	**0.000**	**1.54E-06**
rs12139373	C	0.44	388830	0.989	0.005	0.0375	400461	0.000	0.000	0.0081	**-0.001**	**0.000**	**1.58E-06**
rs7520752	G	0.44	388792	0.989	0.005	0.0395	400422	0.000	0.000	0.0082	**-0.001**	**0.000**	**1.58E-06**
rs1130311	T	0.44	388940	0.989	0.005	0.0338	400572	0.000	0.000	0.0082	**-0.001**	**0.000**	**1.55E-06**
rs4092141	T	0.43	389032	0.987	0.005	0.0157	400655	0.000	0.000	0.0082	**-0.001**	**0.000**	**1.79E-06**
rs113817010	A	0.43	385497	0.989	0.005	0.0317	397022	0.000	0.000	0.0084	**-0.001**	**0.000**	**2.16E-06**
rs2836	C	0.43	389017	0.987	0.005	0.0146	400641	0.000	0.000	0.0085	**-0.001**	**0.000**	**1.86E-06**
rs373494891	AT	0.42	381625	0.987	0.005	0.0168	393027	0.000	0.000	0.0087	**-0.001**	**0.000**	**2.15E-06**
rs11240352	C	0.44	388767	0.989	0.005	0.0411	400392	0.000	0.000	0.0089	**-0.001**	**0.000**	**1.95E-06**
rs61822626	C	0.43	389032	0.987	0.005	0.0168	400654	0.000	0.000	0.0090	**-0.001**	**0.000**	**2.05E-06**

rs11240369	A	0.43	388951	0.987	0.005	0.0157	400570	0.000	0.000	0.0091	**-0.001**	**0.000**	**2.10E-06**
rs114230634	G	0.43	388808	0.988	0.005	0.0236	400443	0.000	0.000	0.0094	**-0.001**	**0.000**	**2.21E-06**
rs113699533	A	0.43	389057	0.988	0.005	0.0201	400676	0.000	0.000	0.0097	**-0.001**	**0.000**	**2.59E-06**
rs746149817	C	0.43	388697	0.988	0.005	0.0226	400332	0.000	0.000	0.0097	**-0.001**	**0.000**	**2.29E-06**
rs12123773	G	0.43	389098	0.988	0.005	0.0205	400719	0.000	0.000	0.0098	**-0.001**	**0.000**	**2.62E-06**
rs12130384	G	0.43	389356	0.988	0.005	0.0197	401001	0.000	0.000	0.0102	**-0.001**	**0.000**	**2.49E-06**
rs6593920	G	0.45	389799	0.997	0.005	0.6265	401453	0.000	0.000	0.0102	-0.001	0.000	8.30E-06
rs12132194	G	0.43	388261	0.987	0.005	0.0139	399857	0.000	0.000	0.0102	**-0.001**	**0.000**	**2.89E-06**
rs753568788	A	0.42	387927	0.988	0.005	0.0188	399509	0.000	0.000	0.0108	**-0.001**	**0.000**	**3.33E-06**
rs17345153	G	0.43	388881	0.987	0.005	0.0166	400493	0.000	0.000	0.0109	**-0.001**	**0.000**	**3.37E-06**
rs61822659	A	0.42	387150	0.986	0.005	0.0081	398735	0.000	0.000	0.0113	**-0.001**	**0.000**	**4.18E-06**
rs144224664	CA	0.43	388691	0.987	0.005	0.0153	400296	0.000	0.000	0.0116	**-0.001**	**0.000**	**3.81E-06**
rs6692706	T	0.43	389008	0.987	0.005	0.0137	400623	0.000	0.000	0.0119	**-0.001**	**0.000**	**4.13E-06**
rs7515178	A	0.47	389128	0.989	0.005	0.0335	400769	0.000	0.000	0.0124	**-0.001**	**0.000**	**1.77E-06**
rs11579220	T	0.42	387167	0.986	0.005	0.0081	398750	0.000	0.000	0.0127	**-0.001**	**0.000**	**5.08E-06**
rs6593921	G	0.47	388947	0.989	0.005	0.0427	400576	0.000	0.000	0.0127	**-0.001**	**0.000**	**1.66E-06**
rs12143085	G	0.47	389100	0.989	0.005	0.0334	400736	0.000	0.000	0.0129	**-0.001**	**0.000**	**1.98E-06**
rs11578293	C	0.47	389067	0.989	0.005	0.0305	400703	0.000	0.000	0.0130	**-0.001**	**0.000**	**1.97E-06**
rs7553876	G	0.47	389158	0.989	0.005	0.0328	400795	0.000	0.000	0.0134	**-0.001**	**0.000**	**2.08E-06**
rs12123922	A	0.42	388885	0.987	0.005	0.0124	400500	0.000	0.000	0.0135	**-0.001**	**0.000**	**5.45E-06**
rs61822566	C	0.47	389093	0.989	0.005	0.0335	400728	0.000	0.000	0.0141	**-0.001**	**0.000**	**2.27E-06**
rs375980630	GGGGAA	0.42	388046	0.988	0.005	0.0201	399631	0.000	0.000	0.0147	**-0.001**	**0.000**	**5.87E-06**
rs2369526	T	0.47	388818	0.990	0.005	0.0539	400449	0.000	0.000	0.0149	**-0.001**	**0.000**	**2.47E-06**
rs6664397	T	0.47	388712	0.990	0.005	0.0517	400337	0.000	0.000	0.0153	**-0.001**	**0.000**	**2.81E-06**
rs6692170	A	0.47	388702	0.989	0.005	0.0341	400327	0.000	0.000	0.0159	**-0.001**	**0.000**	**3.03E-06**
rs9651228	G	0.47	388725	0.990	0.005	0.0531	400350	0.000	0.000	0.0163	**-0.001**	**0.000**	**3.15E-06**
rs7546850	A	0.47	388807	0.990	0.005	0.0473	400433	0.000	0.000	0.0169	**-0.001**	**0.000**	**2.87E-06**
rs12744678	T	0.27	381798	1.027	0.006	7.55E-06	393224	0.000	0.000	0.0722	0.000	0.000	0.2501
rs55678495	A	0.14	**368020**	**1.038**	**0.008**	**8.71E-07**	379058	0.000	0.000	0.2061	0.000	0.000	0.4175
1:204945338	C	0.18	**387578**	**1.032**	**0.007**	**2.92E-06**	399173	0.000	0.000	0.2346	0.000	0.000	0.4488
rs3892308	T	0.18	389331	1.031	0.007	7.27E-06	400967	0.000	0.000	0.3085	0.000	0.000	0.5578

rs3892248	A	0.19	**389799**	**1.031**	**0.007**	**5.98E-06**	401453	0.000	0.000	0.3585	0.000	0.000	0.625
rs2595944	A	0.18	**370065**	**1.032**	**0.007**	**4.17E-06**	381108	0.000	0.000	0.4546	0.000	0.000	0.7954
rs4017874	G	0.18	**385763**	**1.032**	**0.007**	**4.41E-06**	397298	0.000	0.000	0.4591	0.000	0.000	0.7622
rs17415523	C	0.20	378595	1.030	0.007	7.08E-06	389920	0.000	0.000	0.7083	0.000	0.000	0.9379
rs67699525	A	0.18	**378611**	**1.033**	**0.007**	**1.85E-06**	389926	0.000	0.000	0.8067	0.000	0.000	0.8352
rs35068223	T	0.19	**377622**	**1.036**	**0.007**	**1.77E-07**	388907	0.000	0.000	0.8125	0.000	0.000	0.791
rs34870821	T	0.23	388825	1.028	0.006	8.46E-06	400456	0.000	0.000	0.9530	0.000	0.000	0.5819
rs7516408	T	0.23	387794	1.028	0.006	7.82E-06	399403	0.000	0.000	0.9593	0.000	0.000	0.6427

Where: *, conditional.

STable 3: Genetic variants in *CNTN4* with significant or suggestive associations with BMI or risk-taking

SNP	A1	A1F	NMISS	BETA	SE	P_bmi	NMISS	OR	SE	P_risks	NMISS	BETA	SE	P_bmi
rs1145041	T	0.49	393584	0.052	0.011	**1.22E-06**	382116	1.00	0.01	0.4285	386608	0.021	0.031	0.4951
rs4685508	A	0.46	394059	-0.051	0.011	**2.25E-06**	382564	1.00	0.01	0.5719	387049	-0.017	0.023	0.4612
rs7639586	T	0.45	397771	-0.053	0.011	**5.51E-07**	386146	1.00	0.01	0.6091	390656	-0.022	0.026	0.3927
rs1627079	T	0.49	400166	-0.053	0.011	**6.42E-07**	388494	1.00	0.01	0.5738	393044	NA	NA	NA
rs1666326	C	0.49	399815	-0.053	0.011	**4.71E-07**	388150	1.00	0.01	0.5569	392695	NA	NA	NA
rs1720179	G	0.49	400082	-0.053	0.011	**5.12E-07**	388412	1.00	0.01	0.579	392955	NA	NA	NA
rs1685491	T	0.48	400166	-0.052	0.011	**7.49E-07**	388493	1.00	0.01	0.5935	393042	NA	NA	NA
rs4685515	A	0.44	400055	-0.052	0.011	**9.09E-07**	388386	1.00	0.01	0.5982	392899	-0.023	0.027	0.3816
rs12639121	G	0.44	400123	-0.053	0.011	**7.09E-07**	388453	1.00	0.01	0.5997	392968	-0.023	0.027	0.3834
rs1685492	C	0.49	400079	-0.052	0.011	**7.06E-07**	388402	1.00	0.01	0.613	392962	NA	NA	NA
rs1554562	G	0.49	399898	-0.053	0.011	**6.54E-07**	388226	1.00	0.01	0.5857	392796	NA	NA	NA
rs1566382	C	0.49	400122	-0.052	0.011	**8.77E-07**	388448	1.00	0.01	0.6439	393009	NA	NA	NA
rs3856836	C	0.44	400358	-0.052	0.011	**1.01E-06**	388681	1.00	0.01	0.6447	393219	-0.019	0.027	0.4719
rs1666329	A	0.48	394063	-0.054	0.011	**4.38E-07**	382544	1.00	0.01	0.5855	394047	NA	NA	NA
rs3856837	C	0.48	394050	-0.054	0.011	**4.28E-07**	382532	1.00	0.01	0.5902	394050	NA	NA	NA
rs3883937	T	0.48	394050	-0.054	0.011	**4.28E-07**	382532	1.00	0.01	0.5902	394050	NA	NA	NA
rs3856838	T	0.48	394051	-0.054	0.011	**4.28E-07**	382533	1.00	0.01	0.5894	394050	NA	NA	NA
rs1617892	C	0.49	400530	-0.051	0.011	**1.15E-06**	388848	1.00	0.01	0.6361	393407	NA	NA	NA
rs1666331	A	0.49	400540	-0.052	0.011	**1.02E-06**	388858	1.00	0.01	0.6249	393417	NA	NA	NA
3:2388826	CATT	0.49	397663	-0.052	0.011	**1.12E-06**	386078	1.00	0.01	0.5835	392486	NA	NA	NA
rs2728517	T	0.49	400769	-0.051	0.011	**1.24E-06**	389080	1.00	0.01	0.6146	393536	NA	NA	NA
rs1685495	C	0.49	400578	-0.052	0.011	**1.06E-06**	388899	1.00	0.01	0.6466	393345	NA	NA	NA
rs4684343	A	0.44	400807	-0.052	0.011	**1.03E-06**	389124	1.00	0.01	0.6252	393520	-0.017	0.027	0.5346
rs200861416	A	0.44	399988	-0.052	0.011	**9.57E-07**	388320	1.00	0.01	0.6167	392986	-0.015	0.027	0.5715
rs968057	A	0.49	401225	-0.051	0.011	**1.11E-06**	389524	1.00	0.01	0.6324	393890	NA	NA	NA
rs4685517	G	0.49	401044	-0.052	0.011	**9.22E-07**	389345	1.00	0.01	0.6264	393689	NA	NA	NA
rs4684345	A	0.50	388789	0.049	0.011	**5.63E-06**	377420	1.00	0.01	0.3877	381650	-0.019	0.041	0.6396
rs4685518	A	0.38	388064	-0.050	0.011	**4.89E-06**	376735	1.00	0.01	0.6554	380960	-0.019	0.017	0.2779
rs58360846	C	0.38	387436	-0.052	0.011	**2.74E-06**	376107	1.00	0.01	0.7333	380367	-0.023	0.017	0.1895

rs113521342	C	0.38	388168	-0.053	0.011	**1.85E-06**	376843	1.00	0.01	0.6005	381286	-0.022	0.017	0.2109
3:2409882	A	0.38	388195	-0.053	0.011	**1.62E-06**	376861	1.00	0.01	0.6140	381282	-0.022	0.017	0.1971
rs13093013	T	0.38	388347	-0.052	0.011	**2.08E-06**	377016	1.00	0.01	0.5992	381458	-0.022	0.017	0.2150
rs13093020	T	0.38	388338	-0.053	0.011	**1.88E-06**	377006	1.00	0.01	0.6044	381449	-0.022	0.017	0.2073
rs13091719	A	0.38	387262	-0.051	0.011	**3.96E-06**	375942	1.00	0.01	0.6733	380194	-0.021	0.017	0.2196
rs13091946	C	0.38	386937	-0.051	0.011	**4.33E-06**	375633	1.00	0.01	0.6819	379874	-0.022	0.017	0.2035
rs3913590	C	0.38	387387	-0.052	0.011	**3.06E-06**	376064	1.00	0.01	0.6591	380314	-0.022	0.017	0.1973
rs77917680	G	0.32	387560	-0.052	0.011	**6.13E-06**	376239	1.00	0.01	0.5081	380453	-0.025	0.015	0.1026
rs58529380	G	0.38	387421	-0.051	0.011	**3.74E-06**	376099	1.00	0.01	0.6396	380324	-0.020	0.017	0.2386
rs57756639	T	0.38	387229	-0.052	0.011	**2.64E-06**	375912	1.00	0.01	0.6610	380154	-0.023	0.017	0.1814
rs12496950	G	0.38	387346	-0.052	0.011	**3.17E-06**	376028	1.00	0.01	0.6633	380269	-0.021	0.017	0.2129
rs6792956	C	0.43	385901	-0.051	0.011	**3.09E-06**	374629	1.00	0.01	0.9699	378828	-0.019	0.021	0.3528
rs6795825	A	0.37	385741	-0.050	0.011	**6.07E-06**	374479	1.00	0.01	0.8446	378663	-0.023	0.017	0.1771
rs76345211	A	0.13	401506	-0.079	0.016	**8.22E-07**	389799	0.99	0.01	0.2751	394050	-0.078	0.016	**1.67E-06**
rs12632872	T	0.13	400137	-0.079	0.016	**8.88E-07**	388468	0.99	0.01	0.2024	392700	-0.078	0.016	**1.72E-06**
rs559108268	TTG	0.13	382478	-0.074	0.016	**3.90E-06**	371311	0.99	0.01	0.1768	375351	-0.074	0.016	**5.42E-06**
rs4685542	C	0.14	398618	-0.075	0.015	**1.07E-06**	386997	0.99	0.01	0.1227	391199	-0.075	0.016	**1.66E-06**
rs2320959	C	0.15	396955	-0.068	0.015	**4.59E-06**	385370	0.99	0.01	0.2470	389568	-0.067	0.015	**9.63E-06**
rs373714481	TTTATTTAC	0.15	394810	-0.073	0.015	**1.16E-06**	383283	0.99	0.01	0.1732	387474	-0.071	0.015	**2.70E-06**
rs6790416	G	0.15	396853	-0.068	0.015	**5.42E-06**	385277	0.99	0.01	0.2335	389467	-0.066	0.015	**1.13E-05**
rs6793002	C	0.15	396838	-0.068	0.015	**5.78E-06**	385261	0.99	0.01	0.2298	389452	-0.066	0.015	**1.20E-05**
rs908495	C	0.20	397406	-0.063	0.013	**2.34E-06**	385820	1.00	0.01	0.8734	390026	-0.062	0.014	**4.22E-06**
rs2320958	A	0.20	397286	-0.063	0.013	**3.25E-06**	385715	1.00	0.01	0.8564	389919	-0.062	0.014	**5.68E-06**
rs908494	A	0.20	397304	-0.063	0.013	**3.16E-06**	385730	1.00	0.01	0.8425	389934	-0.062	0.014	**5.57E-06**
rs2320957	C	0.19	397124	-0.062	0.013	**4.10E-06**	385554	1.00	0.01	0.8564	389754	-0.061	0.014	**7.08E-06**
rs144052920	GTAAA	0.19	391857	-0.062	0.014	**5.55E-06**	380421	1.00	0.01	0.8625	384569	-0.061	0.014	**9.32E-06**
rs58942797	C	0.15	395670	-0.069	0.015	**5.48E-06**	384146	0.99	0.01	0.2957	388312	-0.067	0.015	**1.06E-05**
rs4685546	G	0.19	392133	-0.062	0.014	**5.48E-06**	380726	1.00	0.01	0.8346	384871	-0.061	0.014	**9.03E-06**
rs12631108	A	0.17	385549	-0.065	0.014	**5.54E-06**	374283	1.00	0.01	0.5729	378389	-0.064	0.014	**8.55E-06**
rs62232818	T	0.10	394563	0.007	0.018	0.6820	383065	1.04	0.01	**3.09E-06**	387244	0.013	0.018	0.4825

STable 4: Genetic variants in *CNTN5* significantly associated with neuroticism, systolic and diastolic blood pressure, HbA1c

SNP	A1	A1F	NMISS	BETA	P neuroticism	BETA	P_DBP	BETA	P_SBP	BETA	P_HbA1c
rs76314809	G	0.16	328532	-0.003	0.7730	0.155	**5.25E-06**	0.201	0.0007	0.001	0.2587
rs11220280	T	0.30	324751	0.040	**2.90E-06**	-0.009	0.7520	-0.060	0.2113	0.000	0.2701
rs10501915	T	0.30	327559	0.040	**3.51E-06**	-0.008	0.7570	-0.054	0.2567	0.000	0.2870
rs10790767	T	0.40	324775	0.042	**2.43E-07**	-0.012	0.6497	-0.058	0.1943	0.000	0.4240
rs10790768	T	0.30	327805	0.041	**2.38E-06**	-0.008	0.7676	-0.049	0.3023	0.000	0.3027
rs10750310	G	0.32	325059	0.039	**5.98E-06**	-0.012	0.6642	-0.054	0.2478	0.000	0.3669
rs10893455	G	0.32	325140	0.039	**6.09E-06**	-0.012	0.6673	-0.055	0.2400	0.000	0.3791
rs10893456	G	0.32	325029	0.039	**6.18E-06**	-0.014	0.6144	-0.059	0.2118	0.000	0.3631
rs149803865	TTTTAA	0.31	321719	0.039	**4.19E-06**	-0.009	0.7509	-0.050	0.2910	0.000	0.3060
11:99397691	A	0.30	327999	0.039	**5.30E-06**	-0.016	0.5678	-0.064	0.1782	0.000	0.3571
rs10750313	C	0.31	328258	0.040	**3.43E-06**	-0.015	0.5945	-0.062	0.1876	0.000	0.3787
rs10893461	T	0.30	328213	0.039	**3.99E-06**	-0.014	0.5992	-0.061	0.1986	0.000	0.3017
11:99399673	A	0.30	324174	0.039	**4.99E-06**	-0.014	0.5999	-0.051	0.2870	0.000	0.3579
rs10790772	C	0.31	328332	0.040	**3.53E-06**	-0.015	0.5703	-0.061	0.1932	0.000	0.3227
rs10790773	A	0.31	328321	0.039	**3.68E-06**	-0.015	0.5712	-0.061	0.1925	0.000	0.3232
rs10893462	G	0.31	328307	0.039	**3.96E-06**	-0.016	0.5665	-0.062	0.1916	0.000	0.3258
rs71475569	CA	0.30	324828	0.040	**4.34E-06**	-0.016	0.5615	-0.073	0.1263	0.000	0.3980
rs10790774	C	0.31	328532	0.039	**4.85E-06**	-0.015	0.5939	-0.060	0.2014	0.000	0.3180
rs11220319	A	0.31	328377	0.040	**3.07E-06**	-0.014	0.6020	-0.059	0.2071	0.000	0.3194
rs11532028	G	0.31	328307	0.039	**3.85E-06**	-0.013	0.6205	-0.059	0.2098	0.000	0.3204
rs113238743	T	0.31	328276	0.039	**3.88E-06**	-0.013	0.6203	-0.059	0.2088	0.000	0.3248
rs71474525	T	0.30	328203	0.039	**4.08E-06**	-0.014	0.6177	-0.061	0.1931	0.000	0.3806
rs10893463	T	0.31	327939	0.040	**2.25E-06**	-0.015	0.5910	-0.061	0.1930	0.000	0.3451
rs10750314	G	0.30	328112	0.040	**3.35E-06**	-0.013	0.6350	-0.060	0.2072	0.000	0.3639
rs10750315	G	0.30	328058	0.040	**3.17E-06**	-0.014	0.6007	-0.061	0.1940	0.000	0.3735
rs11220327	G	0.30	327733	0.040	**2.82E-06**	-0.014	0.6195	-0.063	0.1826	0.000	0.4041
rs10790775	G	0.31	327605	0.040	**2.42E-06**	-0.015	0.5933	-0.062	0.1910	0.000	0.3279
rs10893467	C	0.30	327354	0.040	**2.72E-06**	-0.014	0.5996	-0.064	0.1782	0.000	0.4057
rs10893468	C	0.30	327255	0.040	**2.24E-06**	-0.014	0.6139	-0.062	0.1927	0.000	0.3582

rs10893469	A	0.30	327146	0.040	**2.54E-06**	-0.014	0.5958	-0.064	0.1726	0.000	0.3598
rs10893470	G	0.30	327053	0.040	**2.53E-06**	-0.014	0.6079	-0.064	0.1786	0.000	0.3578
rs11220331	A	0.30	326936	0.040	**3.07E-06**	-0.015	0.5822	-0.066	0.1598	0.000	0.3449
rs10893472	C	0.31	326180	0.039	**5.36E-06**	-0.011	0.6863	-0.062	0.1894	0.000	0.4846
rs10750316	T	0.31	325815	0.039	**5.20E-06**	-0.010	0.7114	-0.058	0.2183	0.000	0.4726
rs10790776	A	0.31	325806	0.039	**5.22E-06**	-0.010	0.7084	-0.058	0.2163	0.000	0.4705
rs10790777	T	0.31	325829	0.039	**4.62E-06**	-0.010	0.7140	-0.059	0.2133	0.000	0.4719
rs11220342	G	0.31	325741	0.039	**5.59E-06**	-0.010	0.7065	-0.057	0.2242	0.000	0.4586
rs10893477	G	0.31	325658	0.039	**5.54E-06**	-0.009	0.7514	-0.057	0.2246	0.000	0.4187
rs10790778	T	0.31	325651	0.039	**5.24E-06**	-0.009	0.7550	-0.057	0.2250	0.000	0.4144
rs10790779	T	0.31	325689	0.039	**5.33E-06**	-0.009	0.7492	-0.057	0.2232	0.000	0.4143
rs10750317	G	0.31	325702	0.039	**4.56E-06**	-0.008	0.7711	-0.056	0.2321	0.000	0.4219
rs10790780	C	0.31	324148	0.040	**3.91E-06**	-0.007	0.7943	-0.056	0.2396	0.000	0.3831
rs10893478	G	0.31	325570	0.039	**4.34E-06**	-0.007	0.7867	-0.055	0.2459	0.000	0.4019
rs10790781	C	0.31	324975	0.039	**4.24E-06**	-0.007	0.7849	-0.051	0.2831	0.000	0.4168
rs10790782	T	0.31	324981	0.039	**4.24E-06**	-0.008	0.7692	-0.051	0.2776	0.000	0.4150
rs10893479	G	0.31	325605	0.039	**3.99E-06**	-0.008	0.7612	-0.054	0.2535	0.000	0.4148
rs10893480	C	0.31	325601	0.039	**4.09E-06**	-0.008	0.7626	-0.054	0.2532	0.000	0.4125
rs10790785	A	0.31	324574	0.039	**6.18E-06**	-0.008	0.7812	-0.055	0.2474	0.000	0.3756
rs10736549	G	0.41	322786	0.041	**4.90E-07**	-0.009	0.7174	-0.065	0.1446	0.000	0.4568
rs10893481	C	0.31	325616	0.039	**4.35E-06**	-0.009	0.7454	-0.056	0.2369	0.000	0.3994
rs10790786	T	0.31	325591	0.039	**5.41E-06**	-0.009	0.7404	-0.057	0.2304	0.000	0.3979
rs10750320	C	0.31	325611	0.039	**5.77E-06**	-0.009	0.7547	-0.056	0.2337	0.000	0.4015
rs10893482	T	0.31	325614	0.039	**5.10E-06**	-0.009	0.7453	-0.057	0.2297	0.000	0.3992
rs10790788	C	0.31	325575	0.039	**4.69E-06**	-0.009	0.7479	-0.056	0.2317	0.000	0.4103
rs12279785	G	0.31	325567	0.039	**4.07E-06**	-0.009	0.7452	-0.057	0.2300	0.000	0.4038
rs10736550	A	0.41	323776	0.041	**3.12E-07**	-0.011	0.6745	-0.063	0.1557	0.000	0.5266
rs10790789	G	0.42	323205	0.040	**9.15E-07**	-0.007	0.7981	-0.063	0.1589	0.000	0.4606
rs61054860	TCATGTAAA	0.40	320102	0.038	**3.36E-06**	-0.009	0.7207	-0.063	0.1646	0.000	0.6969
rs7925532	C	0.40	324319	0.039	**1.27E-06**	-0.005	0.8341	-0.063	0.1580	0.000	0.5506
rs7936053	G	0.41	324134	0.040	**9.38E-07**	-0.005	0.8422	-0.065	0.1462	0.000	0.5579

rs11220772	G	0.32	322720	0.041	**1.79E-06**	-0.012	0.6722	-0.098	0.0386	0.000	0.9972
rs7122777	T	0.32	324723	0.039	**5.18E-06**	-0.007	0.8069	-0.096	0.0419	0.000	0.9848
rs606249	C	0.48	319821	0.039	**1.49E-06**	0.009	0.7253	-0.033	0.4568	0.000	0.8049
rs655610	T	0.48	320328	0.039	**1.11E-06**	0.012	0.6369	-0.029	0.5083	0.000	0.7965
rs584931	C	0.48	320300	0.039	**1.10E-06**	0.012	0.6346	-0.029	0.5079	0.000	0.7623
rs149787271	T	0.08	328261	0.015	0.3295	-0.184	0.0001	-0.380	**4.28E-06**	0.000	0.9075
rs112365137	G	0.08	328423	0.015	0.3158	-0.184	0.0001	-0.379	**4.49E-06**	0.000	0.8967
rs113555997	G	0.08	328417	0.015	0.3212	-0.183	0.0001	-0.378	**4.83E-06**	0.000	0.9255
rs1452574	A	0.24	322296	-0.002	0.7975	0.137	**3.06E-06**	0.190	0.0002	0.000	0.8801
rs1452575	G	0.25	321787	0.000	0.9981	0.137	**2.68E-06**	0.187	0.0002	0.000	0.9989
rs73003149	C	0.06	321644	0.009	0.5653	-0.029	0.5762	0.007	0.9363	-0.003	**2.82E-07**
rs117831801	C	0.06	321509	0.008	0.6266	-0.016	0.7524	-0.005	0.9572	-0.003	**4.99E-07**
rs72992346	C	0.08	320229	0.005	0.7176	0.006	0.8934	0.095	0.2423	-0.006	**3.02E-24**
rs72992349	G	0.08	320631	0.008	0.5993	0.009	0.8470	0.094	0.2459	-0.006	**7.35E-25**
rs1939309	C	0.40	328532	0.002	0.8332	0.216	**4.70E-17**	0.362	**3.71E-16**	0.001	0.0011
rs1939308	T	0.40	327660	0.002	0.8226	0.215	**7.49E-17**	0.361	**5.46E-16**	0.001	0.0014
rs12280410	A	0.40	328532	-0.006	0.4376	-0.135	**1.59E-07**	-0.280	**3.75E-10**	0.001	0.0015
rs866903	A	0.08	326722	0.010	0.4788	0.023	0.6266	0.064	0.4242	-0.007	**5.51E-33**
rs6590793	A	0.49	325348	-0.005	0.5454	-0.120	**2.19E-06**	-0.245	**2.31E-08**	-0.001	0.0013
rs199903594	CT	0.12	318121	0.016	0.1902	0.019	0.6271	0.008	0.9020	-0.006	**8.08E-27**
rs7102640	A	0.37	325825	-0.011	0.1969	-0.128	**1.10E-06**	-0.278	**8.93E-10**	0.001	7.37E-05
rs147861539	A	0.08	326779	0.010	0.4878	0.021	0.6437	0.055	0.4897	-0.007	**1.24E-32**
rs201835771	C	0.08	325763	0.012	0.4123	0.026	0.5755	0.064	0.4253	-0.007	**2.16E-33**
rs72992361	A	0.08	327045	0.012	0.3990	0.023	0.6217	0.055	0.4958	-0.007	**1.10E-32**
rs1502277	C	0.32	321451	-0.007	0.4038	0.173	**2.05E-10**	0.306	**7.45E-11**	0.000	0.6659
rs6590794	C	0.45	323855	0.006	0.4836	0.171	**2.21E-11**	0.326	**1.66E-13**	-0.002	**1.94E-08**
rs17095168	T	0.08	327191	0.012	0.4115	0.026	0.5725	0.058	0.4702	-0.007	**3.79E-33**
rs6590795	C	0.45	323878	0.006	0.4631	0.171	**1.99E-11**	0.326	**1.62E-13**	-0.002	**1.56E-08**
rs6590796	T	0.32	321545	-0.007	0.4167	0.173	**1.86E-10**	0.306	**6.92E-11**	0.000	0.6350
11:100429834	GC	0.45	320272	0.006	0.4393	0.172	**2.43E-11**	0.334	**5.42E-14**	-0.002	**2.17E-08**
rs10894981	T	0.32	320931	-0.008	0.3697	0.173	**1.77E-10**	0.304	**9.12E-11**	0.000	0.6742

rs72992370	T	0.08	327218	0.012	0.4218	0.027	0.5548	0.059	0.4602	-0.007	**4.25E-33**
rs10791419	C	0.45	324067	0.006	0.4328	0.172	**1.86E-11**	0.326	**1.54E-13**	-0.002	**1.15E-08**
rs1393354	G	0.45	324080	0.006	0.4267	0.171	**2.02E-11**	0.325	**1.81E-13**	-0.002	**1.11E-08**
rs1393353	A	0.45	324090	0.006	0.4264	0.171	**2.00E-11**	0.325	**1.86E-13**	-0.002	**1.12E-08**
rs11224277	T	0.12	325095	0.027	0.0328	0.046	0.2471	0.136	0.0468	-0.005	**2.00E-23**
rs11224278	A	0.08	327263	0.012	0.4179	0.027	0.5642	0.058	0.4674	-0.007	**6.94E-33**
rs962259	C	0.45	324327	0.007	0.4128	0.171	**2.02E-11**	0.326	**1.57E-13**	-0.002	**1.18E-08**
rs962258	C	0.45	324579	0.006	0.4341	0.171	**1.96E-11**	0.327	**1.43E-13**	-0.002	**1.12E-08**
rs7947335	C	0.40	322820	-0.003	0.7419	0.165	**1.70E-10**	0.296	**3.67E-11**	-0.002	**6.69E-10**
rs1502275	G	0.08	327489	0.012	0.4043	0.024	0.6077	0.048	0.5449	-0.007	**4.93E-33**
rs10791420	C	0.43	328532	0.010	0.2033	0.184	**4.60E-13**	0.352	**1.42E-15**	-0.002	**2.04E-09**
rs4754688	T	0.43	328386	0.010	0.1999	0.184	**5.64E-13**	0.350	**1.92E-15**	-0.002	**1.67E-09**
rs4754689	G	0.38	326040	0.001	0.9024	0.179	**4.55E-12**	0.321	**7.89E-13**	-0.002	**7.84E-11**
rs4754690	T	0.43	328260	0.011	0.1798	0.183	**6.20E-13**	0.350	**2.01E-15**	-0.002	**1.76E-09**
rs35165060	C	0.33	326603	0.007	0.4113	0.198	**1.97E-13**	0.376	**5.75E-16**	0.000	0.3436
rs10894983	G	0.08	327501	0.012	0.4077	0.022	0.6336	0.042	0.5962	-0.007	**3.36E-33**
rs12365580	A	0.15	324075	0.015	0.1688	0.032	0.3730	0.055	0.3700	-0.004	**1.96E-18**
rs35091610	T	0.28	324724	-0.004	0.6146	0.200	**6.93E-13**	0.355	**1.64E-13**	0.000	0.5511
rs11224282	A	0.09	327252	0.019	0.1672	0.206	**2.26E-06**	0.411	**4.88E-08**	0.001	0.0282
rs146214587	GT	0.08	326322	0.011	0.4479	0.040	0.3944	0.084	0.2981	-0.007	**8.55E-34**
rs11224283	C	0.08	327506	0.013	0.3661	0.028	0.5482	0.055	0.4897	-0.007	**1.61E-33**
rs11224284	A	0.08	327512	0.013	0.3669	0.027	0.5535	0.055	0.4916	-0.007	**1.65E-33**
rs11224285	G	0.08	327510	0.013	0.3637	0.027	0.5541	0.054	0.4968	-0.007	**1.22E-33**
rs66518530	T	0.09	327275	0.019	0.1694	0.206	**2.27E-06**	0.413	**4.27E-08**	0.001	0.0292
rs1216575	G	0.22	325213	-0.001	0.8755	0.145	**1.30E-06**	0.258	**6.44E-07**	0.000	0.6501
rs1216576	A	0.22	324746	-0.001	0.9113	0.144	**1.64E-06**	0.256	**8.29E-07**	0.000	0.5995
rs1216577	G	0.22	325111	-0.001	0.8782	0.145	**1.27E-06**	0.258	**6.42E-07**	0.000	0.6369
rs11224286	G	0.17	317546	0.018	0.0924	0.142	4.49E-05	0.288	**1.62E-06**	0.001	0.0887
rs1216578	T	0.23	325132	-0.001	0.8910	0.142	**1.81E-06**	0.261	**4.24E-07**	0.000	0.6449
rs2845930	A	0.46	323525	-0.010	0.2114	-0.192	**6.29E-14**	-0.357	**6.96E-16**	0.002	**1.37E-06**
rs67786711	C	0.10	326723	0.018	0.1758	0.190	9.20E-06	0.374	**4.82E-07**	0.001	0.0253

rs10894984	T	0.15	326202	0.017	0.1351	0.028	0.4386	0.044	0.4839	-0.004	**2.64E-19**
rs11224287	G	0.08	327173	0.015	0.3084	0.035	0.4501	0.063	0.4284	-0.007	**3.66E-33**
rs1216579	T	0.40	324689	0.010	0.2174	0.184	**1.21E-12**	0.367	**2.52E-16**	-0.002	**1.22E-08**
rs1216580	C	0.49	324648	0.014	0.0793	0.191	**5.75E-14**	0.356	**5.33E-16**	-0.002	**1.30E-07**
rs1234614	C	0.33	323141	0.001	0.9456	0.113	2.90E-05	0.220	**2.52E-06**	-0.003	**8.69E-13**
rs78355106	AT	0.08	325274	0.017	0.2313	0.037	0.4281	0.071	0.3753	-0.007	**4.96E-31**
rs11224289	A	0.08	327197	0.017	0.2417	0.035	0.4441	0.067	0.3961	-0.007	**2.94E-33**
rs11224290	C	0.08	327214	0.017	0.2431	0.032	0.4848	0.066	0.4071	-0.007	**3.44E-33**
rs744036	T	0.31	325080	0.004	0.6064	0.126	**4.45E-06**	0.246	**2.04E-07**	-0.003	**5.73E-15**
rs11224291	C	0.08	327263	0.017	0.2245	0.031	0.4983	0.062	0.4373	-0.007	**2.05E-33**
11:100445426	C	0.39	323347	0.008	0.3564	0.126	**1.28E-06**	0.228	**4.30E-07**	-0.002	**5.08E-12**
rs7931511	A	0.08	327371	0.017	0.2461	0.031	0.4982	0.069	0.3863	-0.007	**2.41E-33**
rs11224292	A	0.08	327403	0.017	0.2413	0.030	0.5151	0.065	0.4154	-0.007	**2.17E-33**
rs1216528	G	0.49	324806	-0.013	0.0909	-0.186	**2.30E-13**	-0.353	**1.00E-15**	0.002	**1.38E-08**
rs595239	G	0.23	327288	0.001	0.8807	0.135	**6.01E-06**	0.270	**1.74E-07**	0.000	0.5490
rs11224293	A	0.08	327550	0.016	0.2801	0.032	0.4846	0.073	0.3543	-0.007	**3.64E-33**
rs650297	G	0.33	323948	0.002	0.8404	0.110	4.61E-05	0.223	**1.78E-06**	-0.003	**9.54E-13**
rs11224294	C	0.09	328497	0.017	0.2030	0.202	**3.00E-06**	0.405	**6.25E-08**	0.001	0.0284
rs11224295	A	0.09	328532	0.018	0.1971	0.204	**2.57E-06**	0.405	**6.94E-08**	0.001	0.0229
rs635541	A	0.23	328127	0.002	0.8523	0.136	**5.46E-06**	0.275	**9.84E-08**	0.000	0.5709
11:100450188	G	0.09	322888	0.020	0.1565	0.189	1.69E-05	0.384	**4.63E-07**	0.001	0.0164
11:100450266	G	0.10	320255	0.015	0.2650	-0.002	0.9578	0.025	0.7441	-0.006	**2.03E-25**
rs146097009	CAG	0.24	323502	-0.002	0.8365	0.114	0.0001	0.232	**4.88E-06**	0.000	0.7546
rs1939310	A	0.49	326735	0.014	0.0694	0.188	**1.32E-13**	0.362	**1.42E-16**	-0.002	**4.38E-08**
rs66489236	G	0.08	327646	0.016	0.2792	0.031	0.5022	0.072	0.3639	-0.007	**1.03E-33**
rs11224296	T	0.08	327681	0.015	0.2858	0.029	0.5248	0.073	0.3575	-0.007	**1.31E-33**
rs11224297	T	0.10	328074	0.018	0.1873	0.189	9.76E-06	0.372	**4.83E-07**	0.001	0.0161
rs11600772	C	0.08	327636	0.016	0.2799	0.029	0.5214	0.071	0.3684	-0.007	**1.73E-33**
rs11606531	T	0.08	327618	0.015	0.2937	0.031	0.5011	0.076	0.3351	-0.007	**1.54E-33**
rs11224298	A	0.10	327967	0.018	0.1680	0.194	**5.61E-06**	0.378	**3.13E-07**	0.001	0.0127
rs72996113	T	0.10	327567	0.017	0.2172	0.084	0.0488	0.150	0.0431	-0.007	**9.14E-37**

rs618781	G	0.39	323064	0.000	0.9855	0.128	**8.88E-07**	0.209	**3.59E-06**	-0.002	**7.08E-12**
rs1783683	G	0.49	321557	0.009	0.2635	0.193	**4.34E-14**	0.334	**3.90E-14**	-0.002	**1.23E-07**
rs11224302	T	0.10	327505	0.016	0.2308	0.089	0.0369	0.147	0.0466	-0.007	**8.25E-37**
rs11224303	A	0.10	327496	0.016	0.2326	0.089	0.0366	0.147	0.0466	-0.007	**8.65E-37**
rs11224304	T	0.10	327499	0.016	0.2323	0.089	0.0361	0.147	0.0465	-0.007	**1.05E-36**
rs72996119	G	0.10	327500	0.017	0.1992	0.089	0.0379	0.144	0.0503	-0.007	**1.08E-36**
rs3016199	A	0.42	323540	0.012	0.1529	0.192	**9.50E-14**	0.350	**4.22E-15**	-0.002	**3.88E-11**
rs661134	G	0.32	323972	0.007	0.4044	0.180	**3.98E-11**	0.333	**1.57E-12**	0.000	0.2189
rs661210	G	0.32	324102	0.007	0.4198	0.180	**4.04E-11**	0.334	**1.42E-12**	0.000	0.2101
rs10894986	A	0.10	327520	0.018	0.1731	0.089	0.0370	0.139	0.0593	-0.007	**5.46E-37**
rs11224306	T	0.35	320602	-0.002	0.8228	-0.233	**3.44E-18**	-0.423	**6.49E-20**	0.001	0.0040
rs625505	G	0.47	322879	0.012	0.1206	0.206	**8.19E-16**	0.356	**8.31E-16**	-0.002	**9.78E-09**
rs11224309	G	0.10	327606	0.018	0.1741	0.089	0.0365	0.140	0.0588	-0.007	**5.19E-37**
rs72996129	C	0.04	324703	-0.032	0.1061	0.298	**1.80E-06**	0.505	**3.02E-06**	0.001	0.1304
rs1216680	T	0.31	323820	0.007	0.3880	0.180	**4.53E-11**	0.336	**1.07E-12**	0.000	0.2444
rs12223916	G	0.10	327625	0.018	0.1745	0.089	0.0361	0.140	0.0581	-0.007	**6.05E-37**
rs1216681	A	0.31	323818	0.007	0.3898	0.180	**4.89E-11**	0.335	**1.31E-12**	0.000	0.2470
rs56821743	C	0.10	326405	0.018	0.1741	0.197	**4.73E-06**	0.375	**4.63E-07**	0.001	0.0192
rs61324436	C	0.10	326397	0.018	0.1769	0.196	**5.22E-06**	0.373	**5.41E-07**	0.001	0.0187
rs658072	G	0.47	322264	0.012	0.1200	0.208	**4.64E-16**	0.363	**2.19E-16**	-0.002	**1.51E-08**
rs639494	C	0.47	322262	0.013	0.1032	0.208	**4.26E-16**	0.361	**3.45E-16**	-0.002	**2.09E-08**
rs679014	T	0.47	322179	0.014	0.0870	0.209	**2.70E-16**	0.365	**1.73E-16**	-0.002	**2.10E-08**
rs7105522	C	0.10	326364	0.018	0.1724	0.197	**4.91E-06**	0.372	**5.71E-07**	0.001	0.0218
rs7125960	C	0.47	322214	0.013	0.1074	0.205	**1.01E-15**	0.358	**5.93E-16**	-0.002	**2.02E-08**
rs118190236	T	0.02	326322	0.020	0.4853	0.240	0.0085	0.453	0.0040	-0.007	**4.41E-08**
rs1604519	G	0.47	322099	0.013	0.1114	0.205	**9.51E-16**	0.359	**4.88E-16**	-0.002	**2.96E-08**
rs1502285	A	0.47	322091	0.013	0.1096	0.205	**9.51E-16**	0.359	**5.06E-16**	-0.002	**2.64E-08**
rs10791421	G	0.15	326584	0.013	0.2557	0.097	0.0063	0.134	0.0295	-0.004	**3.11E-21**
rs1145415	A	0.47	322052	0.013	0.0990	0.205	**9.90E-16**	0.358	**5.93E-16**	-0.002	**1.37E-08**
rs11606890	C	0.10	328532	0.018	0.1683	0.090	0.0331	0.146	0.0465	-0.007	**1.14E-37**
rs1216693	G	0.48	321831	0.012	0.1291	0.206	**7.25E-16**	0.362	**2.78E-16**	-0.002	**4.41E-08**

rs11600132	C	0.10	327728	0.019	0.1460	0.083	0.0504	0.143	0.0509	-0.007	**1.75E-35**
rs11601576	A	0.10	327712	0.019	0.1520	0.084	0.0476	0.146	0.0475	-0.007	**2.79E-35**
rs143707862	TCCCA	0.10	326045	0.018	0.1823	0.076	0.0715	0.136	0.0627	-0.007	**2.76E-36**
rs756218807	T	0.10	325753	0.018	0.1786	0.079	0.0642	0.138	0.0614	-0.007	**1.26E-36**
rs11224314	A	0.10	326460	0.018	0.1849	0.079	0.0616	0.141	0.0548	-0.007	**1.52E-35**
rs80280902	AG	0.10	325759	0.018	0.1788	0.082	0.0543	0.128	0.0831	-0.007	**1.74E-35**
rs11530658	G	0.10	326357	0.018	0.1732	0.078	0.0674	0.140	0.0562	-0.007	**6.92E-36**
rs76871092	A	0.10	326967	0.020	0.1280	0.080	0.0590	0.140	0.0570	-0.007	**5.44E-35**
rs118100922	C	0.10	326965	0.020	0.1312	0.081	0.0587	0.139	0.0593	-0.007	**5.64E-35**
rs11224317	A	0.10	326975	0.020	0.1291	0.079	0.0631	0.138	0.0609	-0.007	**5.06E-35**
11:100482500	G	0.05	311185	0.012	0.4991	0.115	0.0439	0.188	0.0579	-0.007	**2.33E-20**
rs201152353	TA	0.10	326851	0.021	0.1222	0.080	0.0598	0.134	0.0689	-0.007	**5.44E-35**
rs112845240	A	0.09	328216	0.019	0.1865	0.204	6.47E-06	0.415	**1.05E-07**	0.001	0.0550
rs72998145	G	0.10	327323	0.020	0.1367	0.083	0.0504	0.141	0.0559	-0.007	**1.47E-34**
rs117995162	G	0.10	322746	-0.001	0.9687	0.152	0.0003	0.351	**1.03E-06**	-0.001	0.1765
rs1145421	C	0.25	325038	0.013	0.1711	0.120	4.94E-05	0.231	**6.06E-06**	-0.003	**5.65E-11**
rs12224813	T	0.10	327183	0.020	0.1258	0.080	0.0618	0.137	0.0642	-0.007	**8.91E-35**
rs717662	T	0.11	325016	0.014	0.2824	0.083	0.0442	0.145	0.0418	-0.006	**1.96E-30**
rs80135755	T	0.10	322247	0.001	0.9454	0.156	0.0002	0.356	**6.49E-07**	-0.001	0.1931
rs17095235	T	0.09	328140	0.020	0.1633	0.204	6.47E-06	0.421	**7.07E-08**	0.001	0.0616
rs78194778	C	0.10	326671	0.021	0.1191	0.084	0.0506	0.143	0.0539	-0.007	**9.15E-35**
rs74743669	T	0.10	326446	0.021	0.1229	0.087	0.0411	0.145	0.0497	-0.007	**6.65E-35**
rs947867	G	0.10	326264	0.020	0.1419	0.091	0.0336	0.147	0.0481	-0.007	**6.94E-35**
rs594476	G	0.29	318858	-0.019	0.0294	0.135	**1.68E-06**	0.227	**3.13E-06**	0.002	**2.81E-06**
rs749433	T	0.09	326283	0.021	0.1378	0.116	0.0096	0.193	0.0129	-0.007	**7.17E-31**
rs11224331	T	0.09	326855	0.022	0.1136	0.112	0.0126	0.188	0.0150	-0.007	**3.11E-30**
rs675515	A	0.20	320645	-0.003	0.7773	0.148	**3.14E-06**	0.264	**1.58E-06**	-0.001	0.1330
rs76021287	C	0.09	326953	0.022	0.1109	0.107	0.0164	0.185	0.0172	-0.007	**3.79E-30**
rs7934276	C	0.25	324258	-0.028	0.0024	0.189	**1.16E-10**	0.301	**2.91E-09**	0.002	**1.70E-10**
rs11224334	G	0.09	326602	0.023	0.0997	0.108	0.0156	0.185	0.0167	-0.007	**7.10E-30**
rs11224335	T	0.09	326739	0.023	0.0990	0.108	0.0163	0.181	0.0193	-0.007	**4.73E-30**

rs11224336	A	0.09	326716	0.023	0.0942	0.107	0.0172	0.182	0.0190	-0.007	**3.99E-30**
11:100503579	C	0.09	326280	0.021	0.1361	0.106	0.0178	0.181	0.0198	-0.007	**1.58E-29**
rs1502282	C	0.39	324071	-0.005	0.5289	0.211	**7.34E-16**	0.360	**1.71E-15**	0.000	0.6382
rs11224337	A	0.39	324034	-0.005	0.5260	0.210	**9.42E-16**	0.358	**2.41E-15**	0.000	0.6613
rs7944000	A	0.30	326131	0.013	0.1339	0.109	7.07E-05	0.227	**1.67E-06**	0.000	0.4510
rs76578189	T	0.09	327385	0.021	0.1287	0.109	0.0151	0.178	0.0210	-0.007	**1.51E-29**
rs7942811	A	0.31	328260	-0.027	0.0017	0.168	**7.38E-10**	0.263	**2.29E-08**	0.002	**7.73E-12**
rs10894995	A	0.32	328532	-0.027	0.0017	0.165	**1.21E-09**	0.258	**4.33E-08**	0.002	**1.91E-11**
11:100507826	A	0.09	327246	0.021	0.1379	0.109	0.0150	0.180	0.0201	-0.007	**8.84E-29**
rs4753978	C	0.31	328046	-0.027	0.0017	0.168	**6.94E-10**	0.262	**2.54E-08**	0.002	**6.69E-12**
rs11224340	A	0.09	327464	0.022	0.1246	0.113	0.0114	0.188	0.0153	-0.007	**1.71E-29**
rs4754691	T	0.38	326334	0.013	0.1176	-0.255	**1.33E-22**	-0.459	**2.72E-24**	-0.002	**5.30E-09**
rs11224342	C	0.32	325718	-0.028	0.0012	0.174	**1.76E-10**	0.277	**4.10E-09**	0.002	**2.16E-11**
rs10750591	G	0.29	324252	0.007	0.4151	-0.343	**1.74E-34**	-0.601	**2.63E-35**	0.000	0.3918
rs35469123	CTA	0.40	317241	0.023	0.0059	-0.251	**1.08E-21**	-0.429	**3.40E-21**	-0.002	**4.96E-07**
rs916482	C	0.41	322359	0.022	0.0070	-0.251	**4.83E-22**	-0.424	**3.80E-21**	-0.002	**1.48E-07**
rs7932493	A	0.09	326095	0.022	0.1164	0.120	0.0082	0.193	0.0135	-0.007	**4.37E-28**
rs1032778	C	0.30	322248	0.014	0.1126	-0.334	**2.52E-33**	-0.573	**8.76E-33**	0.000	0.5966
rs11224347	A	0.06	326566	0.017	0.2933	0.141	0.0070	0.174	0.0540	-0.006	**1.68E-17**
rs11224348	T	0.06	326598	0.019	0.2528	0.144	0.0059	0.181	0.0462	-0.006	**2.04E-17**
rs75986749	C	0.06	326621	0.016	0.3136	0.140	0.0072	0.175	0.0537	-0.006	**1.12E-17**
rs74788811	A	0.06	326535	0.018	0.2644	0.141	0.0069	0.176	0.0523	-0.006	**1.18E-17**
rs9888185	A	0.32	324231	0.013	0.1195	0.165	**1.22E-09**	0.268	**1.22E-08**	-0.003	**2.07E-12**
rs11224350	A	0.06	326613	0.017	0.3001	0.140	0.0075	0.175	0.0535	-0.006	**1.10E-17**
rs1032779	A	0.25	324511	0.011	0.2387	0.144	**7.15E-07**	0.251	**6.23E-07**	-0.001	0.0083
rs11224351	C	0.06	326433	0.018	0.2848	0.141	0.0072	0.177	0.0504	-0.006	**1.13E-17**
rs2000520	T	0.32	324655	0.013	0.1200	0.165	**1.23E-09**	0.267	**1.37E-08**	-0.003	**1.27E-12**
rs10791423	C	0.28	323529	0.008	0.3640	-0.377	**1.50E-40**	-0.658	**3.24E-41**	0.000	0.6111
rs7480623	G	0.19	320512	-0.022	0.0259	0.091	0.0047	0.200	0.0003	0.002	**1.69E-06**
rs1587647	A	0.31	323435	0.013	0.1266	-0.348	**1.72E-36**	-0.596	**8.39E-36**	0.000	0.5342
rs11607909	T	0.37	322329	-0.024	0.0037	0.161	**1.00E-09**	0.297	**7.63E-11**	0.002	**2.59E-10**

rs7934743	T	0.37	322374	-0.024	0.0034	0.161	**9.55E-10**	0.298	**6.25E-11**	0.002	**2.85E-10**
rs11224353	A	0.31	323632	0.013	0.1309	-0.349	**9.03E-37**	-0.596	**7.38E-36**	0.000	0.5051
rs7924327	T	0.30	324027	0.016	0.0710	0.171	**6.00E-10**	0.292	**1.01E-09**	-0.003	**7.51E-14**
rs7934207	T	0.37	322433	-0.024	0.0036	0.161	**1.02E-09**	0.297	**6.80E-11**	0.002	**2.19E-10**
rs7935202	G	0.37	322454	-0.024	0.0036	0.161	**1.05E-09**	0.297	**6.85E-11**	0.002	**2.01E-10**
rs10894997	A	0.31	323672	0.013	0.1313	-0.350	**7.49E-37**	-0.597	**5.82E-36**	0.000	0.5156
rs1938907	T	0.30	325861	0.015	0.0842	0.171	**5.32E-10**	0.284	**2.35E-09**	-0.003	**5.33E-13**
rs55657437	A	0.31	323735	0.013	0.1311	-0.350	**6.54E-37**	-0.598	**4.06E-36**	0.000	0.5181
rs71476638	A	0.05	321013	0.007	0.7008	-0.339	**1.10E-08**	-0.614	**2.37E-09**	-0.001	0.1517
rs79174253	C	0.06	326991	0.018	0.2632	0.144	0.0059	0.180	0.0459	-0.006	**6.36E-18**
rs75760535	A	0.06	327222	0.019	0.2556	0.144	0.0057	0.180	0.0453	-0.006	**5.27E-18**
rs12221896	T	0.31	324063	0.013	0.1293	-0.349	**1.05E-36**	-0.599	**3.19E-36**	0.000	0.5088
rs11224354	A	0.06	327458	0.018	0.2611	0.144	0.0058	0.179	0.0469	-0.006	**9.79E-18**
rs11224356	G	0.32	328033	0.013	0.1216	0.166	**7.90E-10**	0.268	**1.01E-08**	-0.003	**1.57E-12**
rs12363256	A	0.32	323611	-0.023	0.0063	0.157	**6.38E-09**	0.300	**1.59E-10**	0.002	**6.19E-10**
rs11224357	G	0.30	328251	0.014	0.0985	0.172	**3.48E-10**	0.286	**1.55E-09**	-0.003	**9.75E-13**
rs11224358	A	0.32	327990	0.013	0.1260	0.165	**1.01E-09**	0.266	**1.23E-08**	-0.003	**1.32E-12**
rs12361534	A	0.37	324217	-0.023	0.0046	0.162	**7.38E-10**	0.296	**6.97E-11**	0.002	**2.47E-10**
rs77132106	AT	0.07	317851	0.027	0.0990	0.130	0.0115	0.180	0.0443	-0.006	**2.47E-17**
rs12362972	G	0.37	324219	-0.023	0.0047	0.162	**7.65E-10**	0.296	**7.30E-11**	0.002	**2.38E-10**
rs11224359	T	0.30	328198	0.014	0.0986	0.172	**3.07E-10**	0.288	**1.19E-09**	-0.003	**8.05E-13**
rs11535737	T	0.30	328209	0.014	0.0983	0.172	**3.43E-10**	0.287	**1.34E-09**	-0.003	**9.44E-13**
rs35693891	G	0.37	324246	-0.023	0.0050	0.161	**8.05E-10**	0.295	**7.87E-11**	0.002	**2.60E-10**
rs1938906	C	0.37	324277	-0.023	0.0043	0.161	**9.76E-10**	0.296	**7.38E-11**	0.002	**2.69E-10**
11:100522374	A	0.37	319964	-0.026	0.0018	0.157	**3.14E-09**	0.287	**3.78E-10**	0.002	**3.34E-10**
rs2088952	C	0.30	328532	0.014	0.0981	0.171	**3.71E-10**	0.285	**1.81E-09**	-0.003	**7.37E-13**
rs61908725	G	0.26	322675	-0.020	0.0285	0.134	**3.26E-06**	0.275	**3.10E-08**	0.002	**4.57E-08**
rs11224360	C	0.31	324536	0.013	0.1282	-0.347	**1.90E-36**	-0.599	**2.45E-36**	0.000	0.5031
rs2407694	A	0.30	328364	0.014	0.1036	0.172	**3.67E-10**	0.285	**1.72E-09**	-0.003	**1.03E-12**
rs7941473	G	0.37	324392	-0.023	0.0050	0.160	**9.95E-10**	0.297	**6.10E-11**	0.002	**4.58E-10**
rs2105582	C	0.30	327721	0.014	0.1035	0.170	**5.08E-10**	0.283	**2.38E-09**	-0.003	**1.61E-12**

rs4272757	A	0.30	321042	0.015	0.0902	-0.351	**8.22E-37**	-0.601	**4.82E-36**	0.000	0.5429
rs11530779	T	0.06	326998	0.019	0.2487	0.142	0.0066	0.177	0.0498	-0.006	**5.09E-19**
rs7480411	A	0.28	320981	0.009	0.3407	-0.381	**7.99E-41**	-0.669	**5.21E-42**	0.000	0.7356
rs7480600	T	0.06	326979	0.019	0.2466	0.142	0.0065	0.177	0.0494	-0.006	**4.49E-19**
rs568696696	C	0.02	327217	0.039	0.1431	0.080	0.3382	0.258	0.0753	-0.006	**5.24E-09**
rs375200975	T	0.02	325967	0.025	0.3307	0.067	0.4105	0.200	0.1549	-0.006	**1.00E-07**
rs7484139	G	0.30	320729	0.014	0.1026	-0.351	**9.84E-37**	-0.603	**3.47E-36**	0.000	0.6021
rs2096710	A	0.32	325601	0.014	0.0962	0.163	**1.97E-09**	0.268	**1.18E-08**	-0.002	**5.08E-12**
rs4285842	C	0.32	325364	0.014	0.1030	0.162	**2.52E-09**	0.265	**1.79E-08**	-0.002	**3.04E-12**
rs9711476	A	0.37	320150	-0.026	0.0020	0.164	**4.99E-10**	0.296	**1.01E-10**	0.002	**1.64E-10**
rs11224361	C	0.30	320686	0.015	0.0927	-0.351	**9.31E-37**	-0.602	**3.77E-36**	0.000	0.6434
rs11224362	C	0.32	323169	0.014	0.0917	0.158	**7.42E-09**	0.259	**4.16E-08**	-0.002	**5.38E-12**
rs11224363	C	0.30	320566	0.015	0.0795	0.162	**4.86E-09**	0.281	**4.64E-09**	-0.003	**8.36E-13**
rs4347357	C	0.28	321247	0.009	0.3083	-0.381	**6.33E-41**	-0.669	**4.26E-42**	0.000	0.7484
rs4340009	T	0.30	320713	0.015	0.0898	-0.351	**8.61E-37**	-0.603	**3.35E-36**	0.000	0.6434
rs79853957	G	0.30	321060	0.015	0.0868	-0.351	**8.76E-37**	-0.603	**3.06E-36**	0.000	0.6830
rs75633940	T	0.30	321441	0.015	0.0863	0.164	**3.21E-09**	0.280	**5.25E-09**	-0.003	**1.05E-12**
rs36195752	G	0.37	317651	-0.026	0.0019	0.167	**2.97E-10**	0.299	**7.39E-11**	0.002	**1.98E-10**
rs55869272	T	0.30	322518	0.014	0.0960	-0.354	**1.59E-37**	-0.611	**2.19E-37**	0.000	0.6437
rs11500929	A	0.33	324902	0.012	0.1584	0.169	**3.62E-10**	0.264	**1.54E-08**	-0.002	**4.00E-11**
rs2134303	A	0.31	324990	0.013	0.1377	0.174	**1.72E-10**	0.281	**2.93E-09**	-0.002	**2.30E-11**
rs11224366	G	0.28	325731	0.008	0.3846	-0.375	**2.00E-40**	-0.661	**6.91E-42**	0.000	0.8645
rs78192924	A	0.06	326958	0.019	0.2425	0.137	0.0088	0.165	0.0676	-0.006	**2.30E-18**
11:100531620	C	0.46	318454	0.017	0.0305	-0.258	**1.05E-23**	-0.493	**1.60E-28**	-0.002	**6.56E-08**
rs7111819	G	0.16	325374	0.018	0.0890	0.131	0.0001	0.203	0.0006	-0.004	**5.76E-17**
rs10750593	G	0.31	328348	0.018	0.0395	-0.375	**9.01E-43**	-0.656	**1.34E-43**	0.000	0.4423
rs7127830	C	0.19	325707	0.022	0.0270	0.131	5.08E-05	0.218	9.67E-05	-0.003	**3.28E-15**
rs4753979	C	0.22	323004	-0.004	0.6622	0.165	**4.74E-08**	0.264	**4.19E-07**	0.000	0.2225
rs7117419	C	0.16	325664	0.018	0.0961	0.130	0.0001	0.202	0.0006	-0.004	**3.55E-17**
rs7942556	C	0.28	328330	0.012	0.1785	-0.410	**4.86E-48**	-0.734	**2.86E-51**	0.000	0.4972
rs11224367	G	0.16	325663	0.018	0.0948	0.129	0.0002	0.202	0.0006	-0.004	**3.68E-17**

rs11224368	A	0.16	325716	0.016	0.1392	0.129	0.0002	0.194	0.0010	-0.004	**2.63E-17**
rs79152974	G	0.06	327115	0.019	0.2335	0.136	0.0093	0.168	0.0635	-0.006	**2.15E-18**
rs11224369	A	0.19	325548	0.024	0.0182	0.134	3.13E-05	0.229	3.75E-05	-0.003	**6.61E-15**
rs12221817	A	0.06	327315	0.019	0.2536	0.138	0.0080	0.172	0.0568	-0.006	**1.35E-18**
rs11224372	T	0.28	327105	0.012	0.1732	-0.412	**3.96E-48**	-0.735	**4.91E-51**	0.000	0.4985
rs17095329	A	0.28	327217	0.012	0.1794	-0.411	**5.73E-48**	-0.733	**6.94E-51**	0.000	0.5032
rs3958711	G	0.47	324783	0.024	0.0022	-0.249	**1.18E-22**	-0.448	**2.16E-24**	-0.002	**5.40E-08**
11:100542265	G	0.06	324932	0.020	0.2430	0.162	0.0025	0.201	0.0308	-0.006	**2.05E-19**
rs924647	G	0.02	327751	0.030	0.2779	0.260	0.0033	0.458	0.0027	-0.005	**3.54E-06**
rs7350545	G	0.47	323980	0.024	0.0031	-0.248	**1.71E-22**	-0.443	**7.64E-24**	-0.002	**7.20E-09**
rs6590804	C	0.22	322909	-0.007	0.4695	0.154	**4.38E-07**	0.239	**5.76E-06**	0.001	0.1006
rs10431107	C	0.25	325810	0.016	0.0852	0.156	**1.40E-07**	0.268	**1.85E-07**	-0.003	**9.81E-14**
rs10160451	T	0.17	326914	0.013	0.2182	0.153	6.93E-06	0.301	**3.17E-07**	-0.001	0.0051
rs6590805	C	0.49	325637	0.007	0.3980	-0.223	**1.26E-18**	-0.411	**5.29E-21**	0.001	0.0903
rs61908735	G	0.26	327901	-0.024	0.0076	0.142	**6.02E-07**	0.281	**1.01E-08**	0.002	**2.08E-07**
rs34324928	C	0.21	325752	-0.006	0.5340	0.165	**9.79E-08**	0.268	**5.93E-07**	0.001	0.1449
rs4753980	A	0.49	327337	0.008	0.3098	-0.226	**3.34E-19**	-0.419	**7.16E-22**	0.001	0.1131
rs756756606	A	0.27	326647	-0.025	0.0044	0.138	**1.28E-06**	0.273	**2.79E-08**	0.002	**3.19E-08**
rs11224376	A	0.17	327423	0.017	0.1094	0.131	0.0001	0.202	0.0006	-0.004	**7.40E-16**
rs79398819	A	0.26	326364	-0.023	0.0090	0.139	**1.21E-06**	0.271	**4.41E-08**	0.002	**6.03E-07**
rs201751360	T	0.26	326364	-0.023	0.0090	0.139	**1.21E-06**	0.271	**4.41E-08**	0.002	**6.03E-07**
rs61908736	G	0.27	328532	-0.025	0.0050	0.144	**4.19E-07**	0.281	**1.01E-08**	0.002	**1.15E-07**
rs11224377	A	0.16	327650	0.017	0.1049	0.132	9.42E-05	0.205	0.0005	-0.004	**9.77E-16**
rs201901540	CT	0.09	315481	0.003	0.8299	0.135	0.0035	0.198	0.0132	-0.004	**1.05E-09**
rs10895006	T	0.20	328326	0.012	0.2126	0.161	**3.68E-07**	0.268	**9.55E-07**	-0.003	**4.89E-14**
rs4754692	A	0.49	327651	0.006	0.4197	-0.226	**2.79E-19**	-0.423	**3.18E-22**	0.001	0.0625
rs145548665	A	0.03	328271	0.013	0.5616	-0.387	**1.58E-08**	-0.553	**3.11E-06**	0.000	0.6692
11:100550077	G	0.10	327650	0.004	0.7465	0.173	5.70E-05	0.281	0.0002	-0.004	**8.44E-13**
rs6590806	A	0.25	327337	-0.022	0.0164	0.181	**4.53E-10**	0.334	**3.43E-11**	0.002	9.36E-06
rs10895007	C	0.20	328434	0.012	0.2447	0.164	**2.05E-07**	0.271	**7.54E-07**	-0.003	**2.82E-14**
rs11224378	C	0.20	328437	0.011	0.2487	0.164	**1.96E-07**	0.269	**8.88E-07**	-0.003	**3.08E-14**

rs10160533	C	0.20	328438	0.011	0.2493	0.164	**1.96E-07**	0.269	**8.84E-07**	-0.003	**3.02E-14**
rs10160326	T	0.20	328450	0.011	0.2474	0.164	**2.05E-07**	0.268	**9.44E-07**	-0.003	**3.03E-14**
rs151206225	G	0.01	326884	-0.007	0.8489	-0.511	7.43E-06	-0.943	**1.77E-06**	0.004	0.0181
rs11224379	A	0.20	328473	0.011	0.2470	0.165	**1.84E-07**	0.269	**8.45E-07**	-0.003	**3.78E-14**
rs4754693	A	0.45	326926	-0.010	0.2211	0.240	**2.60E-21**	0.423	**6.08E-22**	-0.001	0.0298
rs4754694	A	0.25	327021	-0.022	0.0160	0.181	**5.14E-10**	0.337	**2.38E-11**	0.002	1.03E-05
rs11224380	A	0.16	327956	0.017	0.1103	0.135	7.12E-05	0.202	0.0006	-0.004	**6.07E-16**
rs11224381	A	0.25	320616	0.019	0.0341	0.022	0.4425	0.065	0.1980	-0.003	**5.16E-12**
rs79124516	T	0.02	328532	-0.023	0.3522	0.030	0.7093	-0.091	0.5155	-0.006	**1.78E-09**
rs11224382	G	0.20	328532	0.012	0.2292	0.163	**2.41E-07**	0.266	**1.16E-06**	-0.003	**3.05E-14**
rs10160586	C	0.20	328460	0.012	0.2364	0.165	**1.69E-07**	0.271	**7.07E-07**	-0.003	**2.79E-14**
rs10160408	T	0.20	328479	0.012	0.2326	0.165	**1.82E-07**	0.270	**7.59E-07**	-0.003	**3.28E-14**
rs10160729	A	0.20	328482	0.012	0.2323	0.165	**1.88E-07**	0.270	**7.84E-07**	-0.003	**3.14E-14**
rs6590807	A	0.20	328457	0.012	0.2350	0.164	**2.02E-07**	0.270	**8.19E-07**	-0.003	**3.17E-14**
rs11224383	G	0.06	328509	0.018	0.2648	0.137	0.0079	0.169	0.0583	-0.006	**2.08E-17**
rs7107791	A	0.20	328226	0.011	0.2777	0.167	**1.39E-07**	0.274	**5.57E-07**	-0.003	**1.96E-14**
rs11224384	T	0.06	328532	0.019	0.2516	0.137	0.0078	0.170	0.0568	-0.006	**1.78E-17**
rs1021794	A	0.20	328479	0.012	0.2379	0.165	**1.77E-07**	0.271	**7.49E-07**	-0.003	**3.20E-14**
rs61908737	A	0.25	325221	-0.021	0.0200	0.182	**4.80E-10**	0.336	**2.81E-11**	0.002	1.07E-05
rs10895008	C	0.20	328445	0.012	0.2423	0.165	**1.77E-07**	0.270	**7.61E-07**	-0.003	**3.13E-14**
rs11224387	C	0.16	327950	0.017	0.1106	0.135	6.86E-05	0.205	0.0005	-0.004	**4.42E-16**
rs61908738	G	0.25	325141	-0.021	0.0203	0.182	**5.16E-10**	0.337	**2.46E-11**	0.002	1.08E-05
rs7938601	T	0.50	325959	-0.006	0.4770	0.253	**1.39E-23**	0.452	**4.36E-25**	-0.001	0.0154
rs11373240	AT	0.20	328259	0.012	0.2335	0.166	**1.46E-07**	0.273	**5.77E-07**	-0.003	**4.69E-14**
11:100556443	G	0.16	326013	0.018	0.0884	0.137	6.11E-05	0.199	0.0008	-0.003	**9.94E-15**
rs76715013	T	0.16	327454	0.019	0.0730	0.138	4.61E-05	0.210	0.0003	-0.004	**1.45E-15**
rs7928871	T	0.50	325797	-0.006	0.4661	0.253	**1.72E-23**	0.454	**3.35E-25**	-0.001	0.0175
rs7929942	G	0.20	328195	0.011	0.2726	0.165	**1.79E-07**	0.277	**4.15E-07**	-0.003	**7.21E-14**
rs12280935	C	0.45	325832	-0.010	0.2222	0.244	**6.59E-22**	0.432	**9.00E-23**	-0.001	0.0305
rs61908739	C	0.25	325566	-0.022	0.0161	0.174	**2.06E-09**	0.328	**6.81E-11**	0.002	2.60E-05
rs77786711	G	0.06	328376	0.016	0.3196	0.150	0.0036	0.190	0.0330	-0.006	**7.17E-17**

rs201445134	AT	0.09	315219	0.018	0.2205	0.073	0.1094	0.119	0.1333	-0.004	**3.16E-13**
rs11224389	A	0.17	327341	0.016	0.1368	0.135	6.28E-05	0.207	0.0004	-0.004	**1.52E-15**
rs77802037	C	0.03	328532	0.012	0.5781	-0.413	**1.16E-09**	-0.587	**5.85E-07**	0.001	0.5700
rs6590808	T	0.17	327297	0.016	0.1321	0.135	6.75E-05	0.208	0.0004	-0.004	**1.37E-15**
rs61908740	A	0.25	324920	-0.022	0.0148	0.173	**2.93E-09**	0.323	**1.46E-10**	0.002	4.91E-05
rs61908741	T	0.25	324923	-0.022	0.0145	0.173	**2.83E-09**	0.323	**1.45E-10**	0.002	4.93E-05
11:100560993	G	0.06	327957	0.019	0.2321	0.151	0.0035	0.192	0.0317	-0.006	**2.71E-17**
rs151184227	G	0.06	328278	0.017	0.2966	0.150	0.0036	0.192	0.0316	-0.006	**5.82E-17**
rs61472500	T	0.28	323884	-0.024	0.0062	0.200	**9.92E-13**	0.375	**1.16E-14**	0.001	0.0002
rs71476642	T	0.18	319670	0.002	0.8271	-0.414	**9.66E-37**	-0.687	**5.95E-34**	0.000	0.5500
11:100562744	G	0.25	321621	-0.024	0.0077	0.171	**5.69E-09**	0.327	**1.20E-10**	0.002	3.95E-05
rs61908744	G	0.25	324323	-0.024	0.0100	0.174	**2.46E-09**	0.322	**1.67E-10**	0.002	6.64E-05
rs61908745	A	0.25	324302	-0.023	0.0102	0.173	**2.82E-09**	0.322	**1.74E-10**	0.002	7.05E-05
rs7947643	G	0.17	326195	0.016	0.1314	0.136	6.20E-05	0.210	0.0003	-0.004	**2.61E-15**
rs11224390	T	0.06	327802	0.018	0.2602	0.152	0.0034	0.191	0.0335	-0.006	**3.35E-16**
rs17712139	G	0.25	324435	-0.023	0.0134	0.171	**3.93E-09**	0.319	**2.26E-10**	0.002	6.07E-05
rs7952198	A	0.16	325469	0.014	0.1871	0.137	5.68E-05	0.209	0.0004	-0.004	**3.19E-15**
rs78901113	A	0.06	327276	0.016	0.3286	0.154	0.0030	0.194	0.0310	-0.006	**4.44E-16**
rs11224393	G	0.06	327214	0.016	0.3350	0.157	0.0026	0.196	0.0298	-0.005	**7.99E-16**
rs73001993	G	0.25	324364	-0.022	0.0149	0.171	**4.74E-09**	0.318	**2.65E-10**	0.002	6.08E-05
rs77474430	CA	0.06	325780	0.020	0.2300	0.158	0.0025	0.198	0.0283	-0.006	**4.87E-16**
rs61621482	G	0.25	324325	-0.022	0.0156	0.170	**4.96E-09**	0.318	**2.63E-10**	0.002	5.75E-05
rs57637131	C	0.25	324236	-0.022	0.0140	0.169	**6.31E-09**	0.316	**3.54E-10**	0.002	5.05E-05
11:100570230	T	0.03	310699	-0.015	0.5372	0.264	0.0009	0.357	0.0093	-0.005	**2.81E-06**
rs7129917	G	0.28	323765	-0.024	0.0074	0.195	**3.85E-12**	0.371	**2.15E-14**	0.001	0.0001
11:100571137	C	0.06	324789	0.019	0.2609	0.150	0.0045	0.186	0.0422	-0.006	**2.70E-16**
rs73573308	G	0.16	325078	0.014	0.1774	0.139	5.00E-05	0.210	0.0004	-0.004	**5.08E-15**
rs34225168	A	0.05	322240	-0.002	0.9329	-0.337	**1.56E-08**	-0.620	**1.80E-09**	-0.001	0.0940
rs1216744	T	0.23	319413	-0.010	0.2658	0.185	**6.65E-10**	0.274	**1.29E-07**	0.001	0.0807
rs750534145	G	0.49	316620	-0.006	0.4242	0.230	**3.79E-19**	0.430	**3.46E-22**	-0.001	0.0044
rs76151625	T	0.04	327033	0.017	0.3898	0.125	0.0511	0.110	0.3205	-0.006	**8.98E-13**

rs7938194	G	0.49	322108	-0.007	0.4093	0.236	**1.74E-20**	0.442	**8.82E-24**	-0.001	0.0043
rs12278242	A	0.27	324954	-0.022	0.0148	0.161	**1.74E-08**	0.324	**5.06E-11**	0.001	0.0017
rs12291857	A	0.27	324954	-0.022	0.0147	0.161	**1.75E-08**	0.324	**5.21E-11**	0.001	0.0016
rs201584625	CA	0.09	327136	-0.024	0.0756	0.055	0.1974	0.153	0.0388	0.003	**4.29E-06**
rs754232464	G	0.38	312817	-0.015	0.0730	0.174	**5.65E-11**	0.365	**2.36E-15**	0.000	0.2543
rs34296918	G	0.05	318809	0.009	0.6544	-0.268	1.02E-05	-0.506	**1.51E-06**	0.000	0.8460
rs10431109	A	0.07	326421	0.014	0.3592	0.164	0.0010	0.192	0.0252	-0.005	**5.82E-13**
rs11224400	T	0.07	326428	0.014	0.3692	0.163	0.0010	0.192	0.0255	-0.005	**5.20E-13**
rs6590810	G	0.49	322760	-0.007	0.4060	0.234	**3.46E-20**	0.439	**1.95E-23**	-0.001	0.0044
rs11224402	G	0.07	326524	0.015	0.3387	0.164	0.0009	0.193	0.0241	-0.005	**5.14E-13**
rs150036452	A	0.07	326529	0.014	0.3583	0.166	0.0008	0.198	0.0211	-0.005	**5.72E-13**
11:100581324	C	0.49	314651	-0.004	0.5813	0.230	**4.18E-19**	0.434	**2.03E-22**	-0.001	0.0070
rs61910470	A	0.07	326530	0.014	0.3530	0.165	0.0008	0.198	0.0212	-0.005	**5.75E-13**
rs6590812	A	0.49	322923	-0.008	0.3002	0.235	**2.36E-20**	0.433	**6.54E-23**	-0.001	0.0027
rs12295008	A	0.27	325518	-0.022	0.0138	0.162	**1.32E-08**	0.323	**5.84E-11**	0.001	0.0014
rs2298609	A	0.47	322158	0.006	0.4302	-0.272	**1.14E-26**	-0.486	**2.95E-28**	0.001	0.0685
rs2298610	G	0.27	325516	-0.022	0.0137	0.162	**1.34E-08**	0.322	**6.02E-11**	0.001	0.0014
rs2298611	T	0.27	325369	-0.022	0.0141	0.164	**9.14E-09**	0.324	**5.40E-11**	0.001	0.0016
rs1502284	T	0.47	322217	0.007	0.3734	-0.272	**1.15E-26**	-0.484	**5.10E-28**	0.001	0.0744
rs73003836	T	0.10	328532	-0.024	0.0725	0.048	0.2621	0.139	0.0591	0.003	**2.01E-06**
rs7945656	A	0.30	325115	-0.025	0.0036	0.188	**1.03E-11**	0.375	**4.07E-15**	0.001	0.0011
rs7106603	C	0.49	322791	-0.007	0.4083	0.243	**1.16E-21**	0.450	**1.54E-24**	-0.001	0.0069
rs143012079	GT	0.03	327560	-0.010	0.6925	0.348	9.76E-06	0.642	**2.42E-06**	0.001	0.6155
rs61910471	T	0.06	325536	0.005	0.7492	0.150	0.0043	0.163	0.0728	-0.004	**1.54E-10**
rs11224405	G	0.30	325055	-0.023	0.0070	0.196	**1.67E-12**	0.384	**1.05E-15**	0.001	0.0012
rs61910472	T	0.07	326469	0.014	0.3634	0.170	0.0006	0.205	0.0167	-0.005	**1.08E-12**
rs61910473	T	0.07	326468	0.014	0.3635	0.170	0.0006	0.206	0.0166	-0.005	**1.08E-12**
rs7926526	C	0.50	322216	-0.008	0.2940	0.260	**1.87E-24**	0.453	**7.20E-25**	0.000	0.2593
rs11224406	A	0.07	326466	0.014	0.3599	0.170	0.0006	0.206	0.0162	-0.005	**1.08E-12**
rs74997273	T	0.26	325346	-0.025	0.0053	0.167	**4.96E-09**	0.329	**2.76E-11**	0.001	0.0012
rs61910474	A	0.07	326466	0.014	0.3599	0.170	0.0006	0.206	0.0162	-0.005	**1.08E-12**

rs61910475	G	0.07	326466	0.014	0.3665	0.169	0.0006	0.206	0.0165	-0.005	**1.09E-12**
rs1502283	G	0.07	326466	0.014	0.3665	0.169	0.0006	0.206	0.0165	-0.005	**1.09E-12**
rs6590814	T	0.47	322182	0.005	0.5379	-0.280	**4.38E-28**	-0.498	**1.30E-29**	0.001	0.0802
rs7944469	T	0.29	324927	-0.025	0.0038	0.198	**9.90E-13**	0.387	**7.84E-16**	0.001	0.0011
rs7945336	T	0.29	324927	-0.025	0.0038	0.198	**9.96E-13**	0.387	**7.86E-16**	0.001	0.0011
rs117594293	T	0.09	325445	-0.023	0.0845	0.056	0.2007	0.135	0.0734	0.003	**5.11E-06**
rs117311034	T	0.09	325459	-0.024	0.0833	0.055	0.2099	0.132	0.0795	0.003	**4.98E-06**
rs11224407	T	0.07	326468	0.014	0.3739	0.169	0.0006	0.206	0.0165	-0.005	**1.12E-12**
rs11224408	A	0.07	326470	0.014	0.3757	0.169	0.0006	0.205	0.0167	-0.005	**1.14E-12**
11:100591414	C	0.46	317520	-0.009	0.2635	0.220	**1.17E-17**	0.402	**1.63E-19**	-0.001	0.0160
11:100591876	T	0.07	324993	0.015	0.3268	0.156	0.0019	0.186	0.0322	-0.005	**7.55E-13**
rs61910477	T	0.07	326469	0.014	0.3764	0.169	0.0007	0.205	0.0169	-0.005	**1.17E-12**
rs12284151	C	0.30	324956	-0.024	0.0053	0.197	**1.13E-12**	0.389	**5.14E-16**	0.001	0.0010
rs552114751	CT	0.27	316911	-0.023	0.0111	0.165	**1.19E-08**	0.332	**3.03E-11**	0.001	0.0047
rs61910478	T	0.27	325318	-0.024	0.0079	0.166	**6.18E-09**	0.330	**2.38E-11**	0.001	0.0011
rs4753982	C	0.42	322730	-0.013	0.1176	0.222	**8.52E-18**	0.406	**9.24E-20**	0.001	0.0196
rs4754696	C	0.42	322727	-0.013	0.1183	0.221	**8.91E-18**	0.406	**9.57E-20**	0.001	0.0196
rs4753983	C	0.30	324925	-0.024	0.0051	0.198	**9.98E-13**	0.390	**3.98E-16**	0.001	0.0010
rs4753984	T	0.47	322098	0.005	0.5079	-0.281	**2.66E-28**	-0.495	**2.58E-29**	0.001	0.0599
rs61910479	T	0.07	326468	0.014	0.3789	0.169	0.0007	0.205	0.0168	-0.005	**1.23E-12**
rs7105451	A	0.49	322100	-0.008	0.2933	0.261	**1.03E-24**	0.453	**8.10E-25**	0.000	0.2051
rs61910480	T	0.27	325208	-0.024	0.0080	0.167	**5.17E-09**	0.330	**2.24E-11**	0.001	0.0010
rs633185	G	0.29	322167	0.008	0.3514	-0.459	**1.94E-59**	-0.787	**2.85E-58**	0.001	0.0817
rs74594440	T	0.03	326701	0.027	0.2151	-0.414	**3.18E-09**	-0.612	**4.36E-07**	0.000	0.8426
rs11224411	A	0.07	326464	0.014	0.3747	0.169	0.0006	0.205	0.0170	-0.005	**1.41E-12**
rs10128687	G	0.50	322157	-0.008	0.3079	0.261	**1.22E-24**	0.454	**6.38E-25**	0.000	0.2837
rs7130275	T	0.47	322197	0.005	0.5244	-0.280	**3.69E-28**	-0.497	**1.86E-29**	0.001	0.0788
rs7103397	T	0.47	322235	0.005	0.5353	-0.280	**3.82E-28**	-0.496	**2.05E-29**	0.001	0.0797
11:100595567	C	0.50	317199	-0.009	0.2707	0.257	**1.59E-23**	0.446	**9.81E-24**	0.000	0.1593
rs7114867	A	0.50	322240	-0.008	0.3101	0.261	**1.25E-24**	0.452	**9.05E-25**	0.000	0.2703
rs7126604	G	0.42	322841	-0.012	0.1251	0.220	**1.39E-17**	0.404	**1.26E-19**	0.001	0.0130

rs7112041	A	0.47	322257	0.005	0.5380	-0.280	**4.21E-28**	-0.496	**2.25E-29**	0.001	0.0828
rs76163145	T	0.07	324028	0.009	0.5545	0.145	0.0029	0.161	0.0548	-0.005	**3.34E-13**
rs11224412	T	0.42	322836	-0.012	0.1273	0.220	**1.35E-17**	0.404	**1.28E-19**	0.001	0.0132
rs11224413	T	0.47	316660	0.006	0.4646	-0.276	**6.93E-27**	-0.488	**4.57E-28**	0.001	0.0618
rs7952055	G	0.47	322146	0.005	0.5258	-0.280	**4.18E-28**	-0.494	**3.97E-29**	0.001	0.0595
rs7928576	C	0.47	322083	0.005	0.5090	-0.278	**8.73E-28**	-0.491	**7.09E-29**	0.001	0.0614
rs79571719	A	0.03	327858	-0.013	0.5872	0.338	1.64E-05	0.624	**4.23E-06**	0.000	0.7125
rs139591012	CCACT	0.03	327593	-0.011	0.6554	0.348	1.01E-05	0.641	**2.51E-06**	0.000	0.6467
rs7126015	G	0.30	324771	-0.023	0.0074	0.197	**1.28E-12**	0.386	**8.57E-16**	0.001	0.0010
rs61910503	A	0.07	326453	0.014	0.3731	0.167	0.0007	0.203	0.0181	-0.005	**1.58E-12**
rs117695557	G	0.02	328462	-0.032	0.2877	-0.501	**2.02E-07**	-0.858	**2.67E-07**	0.003	0.0100
rs12291493	G	0.49	322582	-0.006	0.4635	0.242	**1.92E-21**	0.448	**2.32E-24**	-0.001	0.0078
rs12286062	T	0.46	322587	-0.009	0.2700	0.225	**1.39E-18**	0.409	**2.02E-20**	-0.001	0.0420
rs34025953	G	0.02	328532	-0.032	0.2810	-0.508	**1.27E-07**	-0.872	**1.61E-07**	0.003	0.0094
rs10895011	G	0.49	322626	-0.006	0.4659	0.242	**1.75E-21**	0.448	**2.26E-24**	-0.001	0.0078
rs11224417	C	0.46	322611	-0.009	0.2695	0.225	**1.20E-18**	0.409	**1.90E-20**	-0.001	0.0411
rs6590817	A	0.49	322605	-0.006	0.4618	0.242	**1.72E-21**	0.448	**2.28E-24**	-0.001	0.0077
rs7946913	A	0.39	323403	-0.013	0.1068	0.188	**5.83E-13**	0.368	**3.96E-16**	0.001	0.1107
rs78894707	A	0.07	326444	0.013	0.3853	0.169	0.0006	0.205	0.0168	-0.005	**1.36E-12**
rs77431356	T	0.07	326460	0.013	0.3896	0.169	0.0007	0.205	0.0170	-0.005	**1.38E-12**
rs117241374	C	0.07	326437	0.013	0.3900	0.169	0.0006	0.206	0.0162	-0.005	**1.27E-12**
rs6590818	C	0.30	324775	-0.024	0.0056	0.198	**9.87E-13**	0.388	**6.31E-16**	0.001	0.0009
rs150999923	TC	0.07	324830	0.017	0.2728	0.156	0.0017	0.189	0.0286	-0.005	**3.65E-12**
rs141913306	CT	0.05	325347	-0.022	0.2068	0.355	**1.42E-10**	0.571	**2.45E-09**	0.001	0.4300
11:100611020	G	0.49	318042	-0.005	0.5612	0.239	**1.03E-20**	0.443	**1.74E-23**	-0.001	0.0031
rs7930157	G	0.49	322601	-0.006	0.4403	0.242	**1.89E-21**	0.447	**2.81E-24**	-0.001	0.0072
rs61910504	G	0.07	326444	0.014	0.3790	0.169	0.0006	0.206	0.0166	-0.005	**1.90E-12**
rs61910505	G	0.27	325193	-0.024	0.0082	0.165	**6.92E-09**	0.327	**3.44E-11**	0.001	0.0010
rs10686612	CTG	0.49	317669	-0.005	0.5489	0.242	**4.24E-21**	0.447	**6.94E-24**	-0.001	0.0022
rs80276828	C	0.03	327872	-0.013	0.6034	0.341	1.40E-05	0.630	**3.49E-06**	0.000	0.7234
rs7120147	C	0.31	324664	-0.023	0.0088	0.193	**1.88E-12**	0.366	**1.34E-14**	0.001	0.0025

rs142374585	TGTGC	0.46	317606	-0.008	0.3229	0.223	**5.25E-18**	0.405	**9.09E-20**	-0.001	0.0251
rs11224426	A	0.31	324879	-0.023	0.0066	0.196	**8.33E-13**	0.370	**6.19E-15**	0.001	0.0014
rs4754697	A	0.46	322533	-0.009	0.2664	0.225	**1.20E-18**	0.408	**2.42E-20**	-0.001	0.0470
rs115034314	C	0.03	328248	-0.012	0.6356	0.334	1.85E-05	0.609	**6.17E-06**	0.000	0.8447
rs75695106	CA	0.48	319310	-0.007	0.3936	0.238	**1.41E-20**	0.439	**3.66E-23**	-0.001	0.0014
rs11824393	T	0.29	326749	-0.027	0.0019	0.194	**3.41E-12**	0.391	**5.71E-16**	0.001	0.0039
rs11824432	A	0.29	326901	-0.027	0.0022	0.193	**4.54E-12**	0.388	**9.35E-16**	0.001	0.0038
rs73005719	C	0.04	325500	-0.040	0.0338	0.368	**7.93E-10**	0.594	**9.55E-09**	0.001	0.3032
rs61910508	T	0.29	327142	-0.021	0.0146	0.176	**2.45E-10**	0.361	**4.96E-14**	0.001	0.0551
rs80333418	A	0.07	326757	0.017	0.2631	0.171	0.0006	0.210	0.0144	-0.004	**5.34E-12**
rs12223958	C	0.07	326776	0.018	0.2522	0.169	0.0007	0.205	0.0169	-0.004	**4.77E-12**
rs4087602	T	0.29	327400	-0.021	0.0139	0.173	**3.97E-10**	0.358	**8.67E-14**	0.001	0.0527
11:100622087	C	0.29	325013	-0.020	0.0213	0.170	**9.08E-10**	0.350	**3.41E-13**	0.001	0.0507
rs201443872	AT	0.07	315270	0.016	0.3044	0.180	0.0003	0.206	0.0154	-0.004	**8.11E-11**
rs7117515	G	0.29	327444	-0.021	0.0147	0.173	**4.13E-10**	0.358	**8.70E-14**	0.001	0.0481
rs4337001	G	0.29	327402	-0.021	0.0145	0.174	**3.75E-10**	0.358	**8.31E-14**	0.001	0.0529
rs2088953	A	0.29	327422	-0.021	0.0146	0.173	**4.18E-10**	0.357	**9.37E-14**	0.001	0.0526
rs2407750	A	0.29	327425	-0.021	0.0147	0.173	**4.22E-10**	0.357	**9.45E-14**	0.001	0.0522
rs11224430	G	0.07	326751	0.018	0.2587	0.169	0.0007	0.206	0.0165	-0.004	**5.25E-12**
rs73005729	C	0.29	327445	-0.021	0.0148	0.173	**4.53E-10**	0.357	**9.68E-14**	0.001	0.0494
rs61892334	A	0.29	327452	-0.021	0.0146	0.173	**4.49E-10**	0.357	**9.69E-14**	0.001	0.0487
rs7947761	G	0.29	327452	-0.021	0.0146	0.173	**4.49E-10**	0.357	**9.69E-14**	0.001	0.0487
rs78737960	G	0.03	328260	-0.017	0.4934	0.341	1.00E-05	0.627	**2.70E-06**	0.000	0.8890
rs7116153	G	0.30	327753	-0.021	0.0130	0.177	**1.28E-10**	0.370	**7.23E-15**	0.001	0.0342
rs7130855	T	0.30	327712	-0.022	0.0123	0.176	**1.52E-10**	0.370	**7.84E-15**	0.001	0.0354
rs4495864	C	0.49	324427	-0.007	0.3455	0.242	**1.53E-21**	0.457	**2.00E-25**	-0.001	0.0026
rs17095405	G	0.30	327753	-0.021	0.0129	0.177	**1.29E-10**	0.370	**7.52E-15**	0.001	0.0341
rs61892352	A	0.30	327851	-0.021	0.0133	0.177	**1.26E-10**	0.371	**6.11E-15**	0.001	0.0365
rs58727919	G	0.30	327851	-0.021	0.0141	0.176	**1.49E-10**	0.370	**7.34E-15**	0.001	0.0377
rs12226782	A	0.07	326777	0.017	0.2627	0.170	0.0006	0.210	0.0144	-0.004	**7.00E-12**
rs61892355	T	0.30	327748	-0.021	0.0173	0.174	**2.87E-10**	0.365	**1.91E-14**	0.001	0.0494

rs9919648	G	0.49	324537	-0.008	0.3291	0.242	**1.50E-21**	0.458	**1.49E-25**	-0.001	0.0027
rs4754698	G	0.46	324749	-0.006	0.4779	0.206	**6.04E-16**	0.393	**4.47E-19**	-0.001	0.0032
rs12221887	T	0.07	326844	0.018	0.2556	0.170	0.0006	0.209	0.0151	-0.004	**5.46E-12**
rs61890760	A	0.30	328257	-0.022	0.0094	0.177	**1.05E-10**	0.372	**4.78E-15**	0.001	0.0530
rs17095409	C	0.30	328261	-0.022	0.0097	0.177	**1.11E-10**	0.372	**5.26E-15**	0.001	0.0530
rs12225108	T	0.07	326838	0.018	0.2457	0.171	0.0006	0.210	0.0145	-0.004	**6.74E-12**
rs7126324	T	0.30	328163	-0.022	0.0094	0.178	**1.01E-10**	0.372	**4.88E-15**	0.001	0.0555
rs7125452	A	0.49	324853	-0.008	0.3169	0.239	**4.91E-21**	0.454	**3.76E-25**	-0.001	0.0035
rs4469852	C	0.30	328252	-0.022	0.0099	0.177	**1.12E-10**	0.371	**5.49E-15**	0.001	0.0526
rs11224435	G	0.19	324909	0.020	0.0558	0.152	**3.79E-06**	0.242	2.24E-05	-0.003	**8.31E-10**
rs60708905	T	0.30	328252	-0.022	0.0099	0.177	**1.12E-10**	0.371	**5.49E-15**	0.001	0.0526
rs4517481	G	0.30	328233	-0.022	0.0104	0.177	**1.20E-10**	0.371	**5.41E-15**	0.001	0.0529
rs11224436	T	0.07	326838	0.018	0.2422	0.170	0.0006	0.209	0.0147	-0.004	**7.07E-12**
rs34636774	G	0.04	324593	0.014	0.4997	-0.293	8.64E-06	-0.588	**2.50E-07**	0.000	0.6087
rs11224437	C	0.07	324251	0.021	0.1901	0.161	0.0017	0.188	0.0336	-0.005	**2.24E-13**
rs12792556	T	0.04	324731	0.013	0.5183	-0.300	**5.83E-06**	-0.596	**1.99E-07**	0.000	0.6161
rs7942588	G	0.30	328269	-0.022	0.0099	0.177	**1.31E-10**	0.370	**6.34E-15**	0.001	0.0542
rs7927197	T	0.30	328271	-0.022	0.0099	0.177	**1.30E-10**	0.370	**6.34E-15**	0.001	0.0534
11:100636098	T	0.30	326713	-0.022	0.0127	0.177	**1.21E-10**	0.368	**1.13E-14**	0.001	0.0478
11:100636156	T	0.07	324721	0.015	0.3546	0.152	0.0026	0.185	0.0341	-0.005	**4.27E-12**
rs492237	C	0.49	324857	-0.008	0.3330	0.238	**5.37E-21**	0.454	**4.43E-25**	-0.001	0.0028
rs7105842	C	0.30	328045	-0.022	0.0097	0.177	**1.30E-10**	0.370	**6.48E-15**	0.001	0.0609
rs7120712	A	0.30	328339	-0.022	0.0099	0.172	**3.08E-10**	0.361	**2.45E-14**	0.001	0.0671
rs17713163	G	0.03	326451	0.022	0.3239	-0.431	**8.14E-10**	-0.618	**3.70E-07**	0.000	0.9355
rs17095426	A	0.30	328453	-0.022	0.0093	0.172	**2.95E-10**	0.362	**2.07E-14**	0.001	0.0629
rs17095428	C	0.30	328532	-0.022	0.0111	0.172	**3.24E-10**	0.363	**1.78E-14**	0.001	0.0572
rs140748271	GAA	0.30	326250	-0.023	0.0088	0.174	**2.79E-10**	0.368	**1.06E-14**	0.001	0.0392
rs61890763	A	0.30	328435	-0.022	0.0093	0.172	**3.10E-10**	0.362	**1.92E-14**	0.001	0.0658
rs61890764	T	0.30	328423	-0.022	0.0088	0.172	**3.19E-10**	0.362	**2.03E-14**	0.001	0.0661
rs17095429	A	0.30	328435	-0.022	0.0093	0.172	**3.08E-10**	0.362	**1.92E-14**	0.001	0.0657
rs144709517	G	0.02	327175	0.032	0.2050	0.091	0.2581	0.210	0.1321	-0.006	**6.29E-08**

rs3901516	A	0.30	328145	-0.023	0.0077	0.171	**3.91E-10**	0.361	**2.50E-14**	0.001	0.0678
rs7102634	A	0.30	328197	-0.022	0.0087	0.170	**5.69E-10**	0.357	**4.90E-14**	0.001	0.0663
rs693080	G	0.24	323136	-0.009	0.3148	0.160	**6.29E-08**	0.197	0.0001	0.001	0.1658
rs17095436	T	0.03	328359	-0.015	0.5334	0.334	1.88E-05	0.622	**4.11E-06**	0.000	0.6498
rs7944598	T	0.30	328404	-0.022	0.0090	0.172	**3.01E-10**	0.362	**2.14E-14**	0.001	0.0767
rs79336312	T	0.06	326994	0.009	0.5873	0.158	0.0030	0.180	0.0506	-0.004	**1.44E-09**
rs35132040	C	0.24	320203	-0.012	0.2072	0.163	**3.99E-08**	0.223	1.52E-05	0.001	0.0636
rs17095438	T	0.30	328017	-0.022	0.0125	0.170	**7.11E-10**	0.356	**6.88E-14**	0.001	0.0508
rs17095439	C	0.30	327934	-0.021	0.0134	0.169	**7.24E-10**	0.355	**8.90E-14**	0.001	0.0481
rs7110872	A	0.30	327017	-0.021	0.0142	0.170	**7.48E-10**	0.355	**9.86E-14**	0.001	0.0520
rs598369	C	0.24	323558	-0.010	0.2851	0.159	**7.53E-08**	0.196	0.0001	0.000	0.2004
rs7112228	T	0.30	327271	-0.021	0.0133	0.170	**6.97E-10**	0.356	**7.85E-14**	0.001	0.0587
rs1144128	A	0.24	323891	-0.009	0.3076	0.160	**5.53E-08**	0.196	0.0001	0.000	0.2320
rs11224442	T	0.06	327088	0.010	0.5313	0.158	0.0031	0.185	0.0449	-0.004	**4.05E-09**
rs7112366	T	0.30	327178	-0.021	0.0133	0.170	**7.37E-10**	0.356	**7.96E-14**	0.001	0.0542
rs61890765	G	0.30	327265	-0.021	0.0138	0.170	**6.54E-10**	0.356	**7.60E-14**	0.001	0.0546
rs4121392	T	0.30	327238	-0.021	0.0132	0.170	**6.40E-10**	0.355	**8.37E-14**	0.001	0.0637
rs73007637	T	0.05	325313	-0.033	0.0754	0.357	**1.35E-09**	0.582	**1.12E-08**	0.001	0.2850
rs6590820	G	0.17	325027	0.015	0.1557	0.139	4.32E-05	0.233	7.82E-05	-0.002	**2.96E-08**
rs625761	G	0.25	322334	-0.011	0.2333	0.139	**1.57E-06**	0.156	0.0019	0.000	0.3199
rs11224443	C	0.06	327326	0.010	0.5331	0.157	0.0032	0.186	0.0435	-0.004	**1.23E-09**
11:100645189	A	0.06	327083	0.011	0.5138	0.145	0.0065	0.173	0.0609	-0.004	**1.89E-09**
rs7123718	C	0.11	327227	-0.007	0.5489	0.187	**1.98E-06**	0.331	**1.05E-06**	-0.001	0.1151
rs499241	T	0.28	328532	-0.016	0.0746	0.126	6.72E-06	0.276	**1.10E-08**	0.001	0.0314
rs60799929	A	0.05	328532	0.000	0.9988	0.274	**2.40E-06**	0.466	**3.34E-06**	-0.001	0.0712
rs682847	C	0.32	327150	-0.017	0.0425	0.108	6.80E-05	0.231	**7.89E-07**	0.000	0.3166
rs686056	A	0.32	326298	-0.018	0.0358	0.109	6.21E-05	0.232	**7.40E-07**	0.000	0.2600
rs507695	T	0.32	327096	-0.017	0.0390	0.107	7.14E-05	0.232	**7.00E-07**	0.000	0.3130
11:100647712	C	0.36	315933	-0.017	0.0416	0.149	**2.91E-08**	0.317	**8.53E-12**	0.000	0.1685
rs655922	C	0.42	325924	-0.004	0.6034	0.089	0.0005	0.206	**3.23E-06**	0.000	0.9581
rs148274695	G	0.02	328274	-0.024	0.4196	-0.491	**4.04E-07**	-0.887	**1.21E-07**	0.003	0.0113

rs75057863	G	0.03	328146	-0.026	0.2915	0.364	**4.34E-06**	0.669	**1.03E-06**	0.000	0.7657
rs73007642	T	0.05	326712	-0.026	0.1430	0.333	**3.41E-09**	0.553	**1.41E-08**	0.000	0.9240
rs1867063	A	0.41	320963	0.003	0.6855	0.178	**8.62E-12**	0.258	**9.63E-09**	-0.001	0.0034
rs58051655	G	0.43	323224	0.003	0.7139	0.193	**7.38E-14**	0.284	**1.72E-10**	-0.001	0.0102
rs588615	C	0.44	327406	0.003	0.6704	0.194	**3.23E-14**	0.280	**2.26E-10**	-0.001	0.0133
rs183926634	A	0.03	327751	0.033	0.1387	-0.384	**7.96E-08**	-0.727	**4.37E-09**	0.002	0.1078
rs139790431	C	0.06	326586	0.015	0.3689	0.153	0.0050	0.190	0.0437	-0.004	**2.74E-08**
rs612090	T	0.44	327446	0.003	0.6631	0.194	**2.64E-14**	0.281	**1.97E-10**	-0.001	0.0132
rs595030	T	0.44	327633	0.003	0.7069	0.193	**3.85E-14**	0.280	**2.33E-10**	-0.001	0.0154
rs73007646	T	0.03	328532	0.025	0.3019	-0.323	2.82E-05	-0.646	**1.36E-06**	0.002	0.1198
rs75916535	A	0.03	328206	-0.024	0.3437	0.358	6.57E-06	0.650	**2.20E-06**	0.000	0.8317
rs77797324	G	0.03	328189	-0.024	0.3437	0.357	6.98E-06	0.650	**2.19E-06**	0.000	0.8328
rs79661065	CA	0.06	327357	0.014	0.3934	0.142	0.0077	0.180	0.0514	-0.004	**9.91E-10**
rs660736	A	0.43	327807	0.003	0.7371	0.192	**5.65E-14**	0.282	**1.75E-10**	-0.001	0.0183
rs626558	G	0.43	327698	0.003	0.7319	0.192	**5.63E-14**	0.281	**2.09E-10**	-0.001	0.0173
rs536090	G	0.43	327789	0.003	0.7527	0.192	**6.14E-14**	0.280	**2.19E-10**	-0.001	0.0192
rs509358	C	0.31	326899	-0.006	0.4976	0.189	**6.83E-12**	0.245	**2.47E-07**	-0.001	0.0870
rs71476649	C	0.04	325184	0.022	0.2806	-0.284	1.75E-05	-0.558	**1.02E-06**	0.000	0.7695
rs2407752	G	0.18	326611	0.016	0.1074	0.103	0.0016	0.183	0.0011	-0.002	**4.91E-06**
rs685611	G	0.43	328025	0.003	0.7339	0.193	**4.45E-14**	0.283	**1.50E-10**	-0.001	0.0225
rs600906	T	0.43	328033	0.003	0.7413	0.193	**4.44E-14**	0.283	**1.41E-10**	-0.001	0.0236
rs600453	A	0.43	327491	0.003	0.6750	0.193	**4.44E-14**	0.283	**1.41E-10**	-0.001	0.0227
11:100657852	CA	0.44	324989	0.002	0.7682	0.197	**1.48E-14**	0.294	**3.46E-11**	-0.001	0.0248
rs600372	T	0.43	327750	0.003	0.7120	0.193	**4.18E-14**	0.283	**1.38E-10**	-0.001	0.0228
rs76282170	CT	0.37	326835	-0.001	0.9416	0.172	**5.74E-11**	0.260	**1.04E-08**	0.000	0.4405
rs61890770	A	0.06	327934	0.013	0.4170	0.142	0.0075	0.179	0.0507	-0.004	**4.21E-09**
rs11224446	C	0.06	327936	0.013	0.4230	0.142	0.0075	0.179	0.0511	-0.004	**4.17E-09**
rs582409	T	0.43	328532	0.003	0.7409	0.193	**3.51E-14**	0.283	**1.42E-10**	-0.001	0.0283
rs607890	G	0.43	328426	0.003	0.7083	0.191	**6.23E-14**	0.281	**1.85E-10**	-0.001	0.0257
rs11224447	T	0.06	327890	0.013	0.4216	0.144	0.0065	0.184	0.0454	-0.004	**3.92E-09**
rs622675	G	0.19	327005	-0.008	0.4137	0.153	**1.53E-06**	0.211	0.0001	0.000	0.5505

rs77036212	G	0.49	314055	0.005	0.5398	-0.150	**5.78E-09**	-0.312	**2.68E-12**	0.001	0.0148
rs5794069	A	0.29	319288	0.010	0.2581	-0.285	**7.96E-24**	-0.501	**1.36E-24**	0.001	0.0456
rs662449	A	0.43	328000	0.003	0.6623	0.192	**5.39E-14**	0.277	**3.24E-10**	-0.001	0.0207
rs11224450	A	0.06	327943	0.013	0.4212	0.138	0.0098	0.170	0.0650	-0.004	**6.97E-09**
rs35472062	C	0.43	327718	0.003	0.7204	0.190	**8.68E-14**	0.276	**4.11E-10**	-0.001	0.0211
rs3858412	G	0.43	327667	0.003	0.7160	0.190	**9.72E-14**	0.276	**4.12E-10**	-0.001	0.0234
rs541931	A	0.20	327051	-0.007	0.4927	0.154	**1.21E-06**	0.209	0.0001	0.000	0.5666
rs58232316	G	0.06	328054	0.013	0.4489	0.139	0.0093	0.171	0.0641	-0.004	**7.84E-09**
rs546584	T	0.20	327049	-0.007	0.5005	0.154	**1.26E-06**	0.208	0.0002	0.000	0.5741
rs12793858	G	0.43	327882	0.004	0.6485	0.191	**8.10E-14**	0.275	**4.73E-10**	-0.001	0.0208
rs11823529	C	0.43	327880	0.004	0.6467	0.191	**7.63E-14**	0.275	**4.58E-10**	-0.001	0.0207
rs61999341	A	0.02	328126	-0.032	0.2318	0.403	**2.53E-06**	0.770	**2.07E-07**	0.001	0.5171
rs61890783	G	0.06	328164	0.013	0.4507	0.139	0.0090	0.172	0.0619	-0.004	**7.97E-09**
rs11224457	C	0.06	328164	0.013	0.4498	0.139	0.0090	0.172	0.0620	-0.004	**8.13E-09**
rs537439	A	0.20	327017	-0.006	0.5394	0.153	**1.52E-06**	0.206	0.0002	0.000	0.5435
rs2155140	C	0.43	327766	0.004	0.6182	0.191	**8.20E-14**	0.276	**4.33E-10**	-0.001	0.0226
rs73007660	G	0.03	328322	0.024	0.2920	-0.286	8.57E-05	-0.593	**2.56E-06**	0.001	0.2515
rs614615	T	0.43	327752	0.004	0.6186	0.189	**1.18E-13**	0.274	**5.60E-10**	-0.001	0.0249
rs17651772	A	0.43	327700	0.004	0.6243	0.190	**8.64E-14**	0.275	**4.76E-10**	-0.001	0.0206
rs113672675	G	0.20	326132	-0.006	0.5421	0.157	**8.57E-07**	0.214	0.0001	0.000	0.3960
rs61890784	T	0.06	328218	0.012	0.4585	0.138	0.0096	0.172	0.0623	-0.004	**6.23E-09**
rs116659446	A	0.02	328060	-0.031	0.2435	0.403	**2.66E-06**	0.775	**1.81E-07**	0.001	0.5814
rs79065387	T	0.06	328259	0.012	0.4604	0.135	0.0111	0.166	0.0726	-0.004	**4.52E-09**
rs74694325	A	0.03	326960	0.033	0.1321	-0.428	**5.82E-10**	-0.619	**2.20E-07**	0.000	0.6595
rs528494	T	0.20	327145	-0.006	0.5209	0.151	**2.01E-06**	0.203	0.0002	0.000	0.5999
rs55760506	G	0.37	324818	0.000	0.9889	0.164	**4.18E-10**	0.248	**4.75E-08**	0.000	0.5194
11:100677607	C	0.05	319816	0.004	0.8490	0.148	0.0154	0.171	0.1047	-0.005	**1.42E-09**
rs7941077	G	0.37	327492	0.001	0.9015	0.166	**2.16E-10**	0.246	**5.72E-08**	0.000	0.6208
rs76787621	C	0.02	328008	-0.031	0.2438	0.407	**2.22E-06**	0.779	**1.63E-07**	0.001	0.6316
rs5794070	G	0.19	322688	-0.006	0.5349	0.157	**1.35E-06**	0.216	0.0001	0.000	0.5238
rs74565354	T	0.06	328342	0.013	0.4450	0.136	0.0106	0.169	0.0665	-0.004	**7.64E-09**

rs618291	G	0.48	325933	-0.020	0.0112	0.164	**8.44E-11**	0.289	**4.15E-11**	0.001	0.0363
rs79735088	G	0.02	327974	-0.030	0.2602	0.399	**3.44E-06**	0.765	**2.75E-07**	0.001	0.6272
rs201578748	TC	0.48	324375	-0.022	0.0055	0.165	**7.51E-11**	0.291	**3.30E-11**	0.001	0.0482
rs61890786	A	0.06	328393	0.013	0.4293	0.136	0.0106	0.167	0.0697	-0.004	**5.07E-09**
rs113692841	A	0.02	327967	-0.031	0.2535	0.398	**3.65E-06**	0.764	**2.77E-07**	0.001	0.6377
rs76169825	C	0.02	327957	-0.031	0.2526	0.399	**3.46E-06**	0.764	**2.79E-07**	0.001	0.6412
rs11824259	T	0.02	327956	-0.031	0.2539	0.399	**3.39E-06**	0.765	**2.74E-07**	0.001	0.6413
rs12419467	G	0.43	327403	0.004	0.6365	0.191	**8.51E-14**	0.276	**4.47E-10**	-0.001	0.0205
rs509944	A	0.19	327010	-0.006	0.5269	0.151	**2.09E-06**	0.200	0.0003	0.000	0.6194
rs598702	T	0.02	327899	-0.032	0.2397	0.402	**2.95E-06**	0.761	**3.08E-07**	0.001	0.6143
rs10791427	G	0.43	327255	0.004	0.6159	0.191	**8.14E-14**	0.275	**4.63E-10**	-0.001	0.0197
rs12417529	C	0.45	327162	0.002	0.8159	0.155	**1.01E-09**	0.214	**1.17E-06**	-0.001	0.0916
rs660338	T	0.02	327879	-0.032	0.2361	0.412	**1.74E-06**	0.770	**2.33E-07**	0.001	0.5952
11:100687252	A	0.05	326096	-0.029	0.1015	0.334	**3.89E-09**	0.563	**8.90E-09**	0.000	0.9572
rs680604	A	0.37	326694	0.001	0.8883	0.167	**1.87E-10**	0.247	**5.14E-08**	0.000	0.5966
rs4754701	G	0.45	326910	0.002	0.8406	0.155	**9.80E-10**	0.214	**1.16E-06**	-0.001	0.0981
rs11224467	A	0.06	328369	0.011	0.5177	0.143	0.0074	0.176	0.0560	-0.004	**4.44E-09**
rs616680	G	0.02	327880	-0.033	0.2185	0.412	**1.74E-06**	0.766	**2.75E-07**	0.001	0.6232
rs11224469	A	0.23	317783	-0.006	0.4998	0.206	**1.59E-11**	0.301	**1.18E-08**	0.000	0.3501
rs586495	T	0.20	325961	-0.004	0.7025	0.144	**5.21E-06**	0.190	0.0005	0.000	0.9814
rs17652307	G	0.06	328293	0.010	0.5641	0.141	0.0081	0.178	0.0540	-0.004	**2.74E-09**
rs678681	T	0.20	326460	-0.005	0.5824	0.146	**3.60E-06**	0.199	0.0003	0.000	0.9496
rs11224471	A	0.06	328352	0.011	0.5099	0.144	0.0070	0.179	0.0525	-0.004	**6.05E-09**
rs11224472	G	0.06	328236	0.012	0.4887	0.146	0.0060	0.181	0.0494	-0.004	**4.48E-09**
rs11224473	G	0.06	328329	0.010	0.5457	0.145	0.0063	0.179	0.0519	-0.004	**3.51E-09**
rs650867	G	0.20	326184	-0.006	0.5719	0.148	**2.39E-06**	0.204	0.0002	0.000	0.9590
rs556146	T	0.20	326313	-0.006	0.5691	0.149	**2.00E-06**	0.207	0.0001	0.000	0.9521
rs117387764	A	0.02	327579	0.036	0.1571	0.113	0.1590	0.245	0.0781	-0.006	**1.16E-07**
rs11224474	G	0.44	323369	0.003	0.7028	0.189	**1.98E-13**	0.275	**6.15E-10**	-0.001	0.0088
rs2897755	G	0.06	326527	0.013	0.4467	0.139	0.0102	0.172	0.0654	-0.004	**1.02E-08**
rs78169527	C	0.03	327020	0.008	0.7448	0.090	0.2288	0.018	0.8883	-0.005	**1.42E-07**

rs629864	C	0.34	317555	0.009	0.3069	0.126	**2.62E-06**	0.247	**1.07E-07**	0.000	0.8230
rs561267	G	0.11	325395	-0.005	0.6829	0.131	0.0016	0.161	0.0247	-0.002	**5.86E-06**
rs12785918	G	0.31	321709	-0.004	0.6655	-0.125	**6.10E-06**	-0.212	8.73E-06	0.001	0.0002
rs12809048	T	0.04	324163	-0.022	0.2611	0.285	**3.32E-06**	0.435	4.17E-05	0.000	0.8799
11:100722432	T	0.05	325792	-0.018	0.3103	0.323	**1.96E-08**	0.507	**3.37E-07**	0.000	0.9708
rs117840491	C	0.02	328532	0.031	0.2286	0.091	0.2589	0.307	0.0287	-0.006	**3.34E-08**
rs12295392	A	0.26	327970	-0.004	0.6980	-0.163	**1.81E-08**	-0.299	**2.00E-09**	0.002	6.61E-05
rs73009603	T	0.03	328532	0.011	0.6293	-0.340	**4.14E-06**	-0.663	**2.06E-07**	0.001	0.2100
rs4754705	A	0.26	328280	-0.004	0.6354	-0.162	**1.96E-08**	-0.298	**2.28E-09**	0.001	0.0001
rs55868772	A	0.26	328289	-0.004	0.6301	-0.162	**1.87E-08**	-0.298	**2.32E-09**	0.001	0.0001
rs142071106	C	0.02	328011	-0.038	0.2237	-0.483	**1.06E-06**	-0.881	**2.65E-07**	0.003	0.0375
rs12361074	C	0.06	326867	-0.015	0.3734	0.288	**9.55E-08**	0.465	**6.59E-07**	0.001	0.1521
rs147014235	GGT	0.06	321750	0.031	0.0721	-0.343	**3.12E-10**	-0.506	**7.78E-08**	-0.001	0.4689
rs527476891	G	0.06	321750	0.031	0.0721	-0.343	**3.12E-10**	-0.506	**7.78E-08**	-0.001	0.4689

STable 5: Comparisons with previously reported findings for contactins

Previously published								in UK Biobank										
PMID	Trait	*	Gene	SNP	RA	RAF	OR or BETA	A1	MAF	BETA neuroticism	OR risks	OR smoking	BETA WHR adj BMI	BETA BMI	BETA DBP	BETA SBP	BETA HbA1c	OR T2D
31206164	Cognitive ability in schizophrenia		CNTN1	rs11177934			0.27											
23382691	IgG glycosylation			rs13328933	C	0.95	0.31											
24842889	Parkinson's disease	c		rs1442190		0.03	3.72											
25188341	Dementia and core Alzheimer's disease neuropathologic changes			rs17539289	A	0.05	0.65	A	0.05	0.011	0.99	1.01	0.000	-0.012	0.008	0.009	-0.001	1.00
22925353	Bipolar disorder			rs312273			1.57											
23934736	Metabolite levels (X-11787)			rs312274	A		0.05	A	0.29	0.015	1.00	1.01	0.000	0.010	0.041	-0.034	0.000	1.00
26252872	Cerebral amyloid deposition in APOEe4 non-carriers (PET imaging)			rs4594074	A	0.90	0.46											
29844566	Reaction time	b		rs139386226			0.01											
30072576	Blood protein levels			rs11177623	T	0.67	0.18											
30700811	Antidepressant treatment resistance (> 2 drugs prescribed)			rs138583130			2.50	A	0.01	0.090	1.02	1.03	-0.001	-0.051	-0.058	-0.260	-0.002	0.93
30285260	Schizophrenia	b	CNTN2	rs11240341	T		1.05	T	0.32	-0.003	1.00	1.00	0.000	0.025	0.026	0.025	0.000	1.02
30285260	Schizophrenia	b		rs11240341	T		1.06	T	0.32	-0.003	1.00	1.00	0.000	0.025	0.026	0.025	0.000	1.02
28991256	Schizophrenia	bc		rs11240341	T		1.05	T	0.32	-0.003	1.00	1.00	0.000	0.025	0.026	0.025	0.000	1.02
32541925	Type 2 diabetes			rs11240351	G	0.47	0.03	G	0.4	0.000	1.02	1.00	**0.001**	-0.002	0.010	0.073	0.001	1.03

30072576	Blood protein levels			rs1572995	T	0.90	-0.714	C	0.13	-0.031	0.99	1.00	**-0.001**	0.013	0.043	0.012	-0.002	0.99
26198764	Schizophrenia	b		rs16937	A		1.06	A	0.32	-0.002	0.99	1.01	0.000	0.032	0.036	0.056	0.000	1.02
29875488	Blood protein levels			rs2071533	G	0.87	0.77	T	0.12	-0.026	0.99	1.00	**-0.001**	0.010	0.045	0.020	-0.002	0.98
30643258	Adventurousness	b		rs2229868	T	0.21	0.01											1.02
28031287	Cerebrospinal fluid biomarker levels	a		rs2242000				A	0.18	0.021	1.00	1.01	0.001	0.029	0.017	0.060	0.001	1.04
30038396	Self-reported math ability (MTAG)	b		rs3767297	A	0.15	0.01											
30038396	Highest math class taken	b		rs3820337	T	0.15	0.02	T	0.14	-0.026	0.99	1.00	-0.001	0.018	0.037	-0.027	-0.001	0.98
30239722	Waist-hip ratio	b		rs3903399	T	0.78	0.01	C	0.21	0.016	1.01	1.01	**0.001**	0.033	0.036	0.091	0.001	1.04
32203549	HDL cholesterol levels	b		rs3903399	T	0.79	0.01											
28240269	Blood protein levels	ac		rs41264869	T	0.17	0.39	T	0.18	0.020	1.00	1.01	0.001	0.029	0.021	0.067	0.001	1.04
30038396	Self-reported math ability	b		rs6593920	T	0.55	0.01	G	0.45	-0.004	1.00	1.00	0.000	-0.014	-0.030	-0.043	0.000	0.99
32888493	Red blood cell count	b		rs6662930	C	0.37		C	0.5	0.002	0.99	1.00	0.000	-0.009	-0.047	-0.090	0.000	0.97
27863252	Red blood cell count	b		rs6696846	T	0.49	0.02											
32888493	Red blood cell count	b		rs6696846	T	0.48	-0.02	T	0.5	0.003	0.99	1.00	0.000	-0.008	-0.046	-0.085	0.000	0.98
29875488	Blood protein levels			rs9787409	T	0.15	0.54											
27082954	Peripheral arterial disease (traffic-related air pollution interaction)	b	CNTN3	rs10428206			29.80											
30850646	Frontal fibrosing alopecia	b		rs114108912	C	0.01	2.49	C	0.01	0.003	1.00	0.97	0.000	0.026	0.103	0.013	0.002	1.08
28181694	Pediatric bone mineral content (hip)	ac		rs677171	C	0.07	0.43	C	0.06	-0.019	0.99	1.01	0.000	-0.006	0.012	-0.061	0.001	1.02

30643251	Smoking initiation (ever regular vs never regular) (MTAG)	b		rs11712680	C	0.19	0.01											
22566634	Economic and political preferences			rs13073838	T		0.12											
24665060	Smoking quantity	b		rs1374879				C	0.13	0.009	0.99	1.00	0.000	0.000	0.077	0.157	0.000	1.00
30038396	Educational attainment (years of education)	b		rs1918394	T	0.17	0.01	T	0.17	0.005	1.00	0.98	0.000	-0.042	0.029	0.031	-0.001	0.98
30038396	Educational attainment (MTAG)	b		rs1918394	T	0.17	0.01	T	0.17	0.005	1.00	0.98	0.000	-0.042	0.029	0.031	-0.001	0.98
29497042	Heart rate response to recovery post exercise (30 sec)	b		rs34310778	T	0.57	0.04											
30643251	Smoking initiation (ever regular vs never regular)	b		rs4543050	T	0.82	0.02											
29769521	Heart rate response to recovery post exercise	b		rs6549649	G	0.56	0.33											
29497042	Heart rate response to recovery post exercise (20 sec)	b		rs6549653	C	0.52	0.03	T	0.48	0.009	0.99	1.01	0.000	0.009	-0.059	-0.131	0.000	1.01
27089180	Age at first sexual intercourse	b		rs6549665	G	0.18	0.03	G	0.18	0.005	1.01	0.98	0.000	-0.040	0.040	0.058	-0.001	0.98
30224653	Systolic blood pressure	bc		rs9857362	A	0.53	0.15											
2180562	Stimulated adipocyte lipolysis	a	CNTN4	rs1032784	G	0.75	0.07	A	0.23	0.002	1.00	1.01	0.000	0.002	0.022	-0.044	0.000	0.99
31596458	Sensorimotor dexterity			rs11129200	CTT		0.89											
30285260	Schizophrenia	b		rs11708578	G		1.08											

28991256	Schizophrenia	bc	rs11708578	G		1.08											
22449649	Intelligence	a	rs11713158			5.04	T	0.20	0.000	1.00	1.00	0.000	0.000	-0.004	-0.038	0.000	0.98
30643251	Smoking initiation (ever regular vs never regular)	b	rs11713899	C	0.17	0.02	C	0.17	-0.024	1.00	1.01	0.000	0.032	0.029	0.070	0.000	0.99
30643251	Smoking initiation (ever regular vs never regular) (MTAG)	b	rs11713899	C	0.17	0.01	C	0.17	-0.024	1.00	1.01	0.000	0.032	0.029	0.070	0.000	0.99
23823483	Metabolite levels		rs13078307			0.23	A	0.24	-0.015	1.00	1.01	0.000	0.006	-0.047	-0.118	0.000	0.99
30019117	Adolescent idiopathic scoliosis	a	rs1479537														
26634245	Post bronchodilator FEV1/FVC ratio	b	rs150343078	T	1.00	0.44											
24556642	Pit-and-Fissure caries	a	rs17013524	T	0.06	1.83											
29503163	Response to aripiprazole in schizophrenia	ac	rs17022006	G		7.16											
23471985	Brain connectivity	a	rs17024684				T	0.08	0.030	1.02	1.00	0.000	0.003	-0.011	-0.019	0.000	1.00
31268507	Schizophrenia	b	rs17194490				T	0.16	-0.013	1.00	1.01	0.000	-0.009	-0.085	-0.097	0.000	0.97
31740837	Schizophrenia	b	rs17194490			1.10	T	0.16	-0.013	1.00	1.01	0.000	-0.009	-0.085	-0.097	0.000	0.97
26198764	Schizophrenia	b	rs17194490	T		1.10	T	0.16	-0.013	1.00	1.01	0.000	-0.009	-0.085	-0.097	0.000	0.97
25056061	Schizophrenia	bc	rs17194490	T	0.16	1.10	T	0.16	-0.013	1.00	1.01	0.000	-0.009	-0.085	-0.097	0.000	0.97
28540026	Autism spectrum disorder or schizophrenia	b	rs17194490	T		1.09	T	0.16	-0.013	1.00	1.01	0.000	-0.009	-0.085	-0.097	0.000	0.97

30804565	Morningness	b	rs17786957	C	0.16	0.02	C	0.16	-0.012	1.00	1.01	0.000	-0.011	-0.089	-0.108	0.000	0.97
32066700	Serum immune biomarker levels	a	rs182265848	T	0.01	58.50											
27989323	RANTES levels		rs186018483	C		0.27											
23823483	Metabolite levels		rs2063896			0.20											
19451621	Amyotrophic lateral sclerosis	c	rs2619566	G		3.03											
30595370	Morning person	b	rs34581681				A	0.15	-0.024	1.00	1.01	0.000	-0.001	-0.081	-0.136	0.000	0.98
30038396	Self-reported math ability (MTAG)	b	rs34877519	T	0.16	0.02	T	0.16	-0.015	1.00	1.02	0.000	-0.007	-0.085	-0.099	0.000	0.97
31005972	Intake of total sugars	b	rs35058156	A	0.51	0.74											
30696823	Chronotype	bc	rs35346733	G	0.81	1.03											
29483656	Schizophrenia	bc	rs35346733	?		1.08											
31835028	anorexia nervosa, attention-deficit/hyperactivity disorder, autism spectrum disorder, bipolar disorder, major depression, obsessive-compulsive disorder, schizophrenia, or Tourette syndrome (pleiotropy)	b	rs35346733	?			A	0.2	-0.014	1.01	1.01	0.000	-0.001	-0.064	-0.112	0.000	0.98
28044437	Body mass index (change over time) in chronic obstructive pulmonary disease	a	rs41526344			0.13	A	0.09	0.014	1.03	0.99	0.000	0.004	-0.069	-0.195	-0.001	0.99

17903302	Blood pressure		rs4370013				T	0.17	0.003	1.01	1.01	0.000	-0.036	-0.037	-0.080	-0.001	0.99
31374203	Cognitive ability, years of educational attainment or schizophrenia (pleiotropy)	b	rs4561819			0.02											
26198764	Schizophrenia		rs4685495	A		1.29											
26087016	Middle childhood and early adolescence aggressive behavior	b	rs4685500	T	0.19												
23823483	Metabolite levels		rs4685577			0.18	G	0.44	-0.008	1.00	0.99	0.000	-0.003	-0.016	-0.032	0.000	1.01
23823483	Metabolite levels		rs4685577			0.20	G	0.44	-0.008	1.00	0.99	0.000	-0.003	-0.016	-0.032	0.000	1.01
23823483	Metabolite levels		rs4685577			0.19	G	0.44	-0.008	1.00	0.99	0.000	-0.003	-0.016	-0.032	0.000	1.01
27126917	Daytime sleep phenotypes	a	rs62246964	C		0.09											
30285260	Schizophrenia	b	rs66492629	A		1.10	A	0.15	-0.014	1.00	1.02	0.000	-0.005	-0.087	-0.115	0.000	0.98
31006051	Atypical femoral fracture in phosphonate treatment		rs6768500	C	0.02	7.63											
30348214	DNA methylation variation (age effect)		rs7636944														
24379826	Serum uric acid levels	a	rs7652782	A	0.06		A	0.12	0.033	1.01	1.02	0.000	-0.004	0.078	0.136	0.000	0.99
31006051	Bisphosphonate-associated atypical femoral fracture		rs76646538	C	0.04	3.93											

28928442	Tuberculosis	b		rs79350564			0.27	G	0.05	-0.021	1.00	1.02	0.000	-0.033	0.158	0.431	0.000	0.98
32572223	Gut microbiota (bacterial taxa, hurdle binary method)			rs79900123	T	0.02	0.94	T	0.02	-0.017	1.01	1.01	0.000	0.020	-0.005	0.113	0.000	1.01
22318345	Gallbladder cancer	ac		rs975334	C	0.16	8.30											
30038396	Highest math class taken (MTAG)	b		rs9845120	T	0.71	0.01											
30038396	Self-reported math ability	b		rs9858503	G	0.72	0.01											
25189868	Blood pressure (smoking interaction)			rs9878978														
29875488	Blood protein levels			rs13071423	A	0.20	0.26											
30072576	Blood protein levels			rs2729284	T	0.53	0.16	C	0.47	-0.010	1.00	1.00	0.000	0.007	-0.013	-0.003	0.000	0.99
29875488	Blood protein levels			rs163352	C	0.81	0.32											
27863252	Basophil percentage of white cells	b		rs163563	T	0.91	0.04											
29403010	White blood cell count (basophil)	b		rs163574			0.05											
27863252	Basophil percentage of granulocytes	b		rs163574	G	0.91	0.04											
27863252	White blood cell count (basophil)	b		rs163574	G	0.91	0.04											
17903304	Atrial fibrillation		CNTN5	rs10501920														
30595370	Neuroticism	b		rs10790767				T	0.40	**0.042**	0.99	1.00	0.000	-0.026	-0.012	-0.058	0.000	1.01
29942085	Neuroticism	b		rs10790767	T		5.48	T	0.40	**0.042**	0.99	1.00	0.000	-0.026	-0.012	-0.058	0.000	1.01
30038396	Highest math class taken	b		rs10893002	T	0.66	0.01	A	0.32	0.027	1.01	1.01	0.000	0.008	-0.028	-0.089	0.000	0.99

30038396	Self-reported math ability (MTAG)	b	rs10893002	A	0.34	0.01	A	0.32	0.027	1.01	1.01	0.000	0.008	-0.028	-0.089	0.000	0.99
26911590	Bone mineral density (femoral neck)	c	rs10893396	C	0.17	0.15	C	0.18	-0.021	1.01	1.00	0.000	0.019	0.010	0.010	0.000	1.01
27629369	Loneliness (multivariate analysis)	b	rs10893420				T	0.26	0.003	0.99	1.00	0.000	-0.021	-0.073	-0.131	-0.001	1.00
30038396	Highest math class taken (MTAG)	b	rs1119257	A	0.32	0.01	A	0.33	0.000	0.99	0.99	0.000	0.017	0.042	0.071	0.000	1.00
29844566	Reaction time	b	rs11218881			0.01	C	0.17	0.015	1.01	1.00	0.000	0.006	-0.023	-0.024	0.000	1.00
22610502	Immune reponse to smallpox (secreted IL-2)	a	rs11223581	G			G	0.04	-0.002	0.98	1.00	0.001	0.059	0.137	0.243	0.000	0.99
26503763	Objective response to lithium treatment in bipolar disorder		rs113262272	A	0.71	1.93											
26365420	Alcohol dependence	b	rs117557854	A	0.02	2.08	A	0.02	0.013	1.01	0.99	0.000	-0.048	-0.070	-0.104	0.002	1.02
30643251	Smoking initiation (ever regular vs never regular) (MTAG)	b	rs12283068	A	0.40	0.01	A	0.40	0.021	1.01	1.01	0.000	0.021	-0.018	-0.060	0.000	0.98
23049088	Myopia (pathological)		rs12803066		0.52		G	0.16	-0.012	1.01	1.00	0.000	-0.036	0.049	0.027	0.000	1.00
30038396	Educational attainment (years of education)	b	rs1301838	T	0.32	0.01	T	0.32	-0.001	0.99	0.99	0.000	0.016	0.039	0.067	0.000	1.00
30038396	Educational attainment (MTAG)	b	rs1301838	T	0.32	0.01	T	0.32	-0.001	0.99	0.99	0.000	0.016	0.039	0.067	0.000	1.00
25673412	Waist-hip ratio	bc	rs1394461	C	0.25	0.03	C	0.22	0.025	1.01	1.01	0.000	-0.007	-0.005	-0.007	0.000	1.00
28240269	Blood protein levels	ac	rs1461672	T	0.06	0.73	T	0.07	-0.011	1.00	1.00	0.000	0.008	0.180	0.251	0.001	1.03

29234017	Protein levels in obesity	a	rs1461674	?		0.23	T	0.07	-0.012	1.00	1.00	0.000	0.010	0.181	0.251	0.001	1.03
31320639	Neurological blood protein biomarker levels	a	rs1461677	T	0.08	0.68	T	0.07	-0.012	1.00	1.00	0.000	0.009	0.178	0.248	0.001	1.03
30038396	Educational attainment (MTAG)	b	rs1461680	A	0.46	0.01	A	0.46	0.021	1.01	1.01	0.000	0.014	-0.004	-0.042	0.000	0.99
31926635	Bipolar disorder or major depressive disorder	b	rs1504721	C		1.02	C	0.39	0.019	1.01	1.00	0.000	-0.007	-0.021	-0.062	0.000	1.00
31969693	Major depressive disorder	b	rs1504721	C	0.40	1.07											
31969693	Major depressive disorder in trauma-unexposed individuals	b	rs1504722	G	0.40	1.14	G	0.39	0.019	1.01	1.00	0.000	-0.007	-0.021	-0.063	0.000	1.00
30642921	Factor VII activity	b	rs1553515	C	0.94	0.02	T	0.06	0.017	0.99	0.99	0.000	0.002	-0.044	-0.086	0.000	1.00
29292387	Neuroticism (MTAG)	b	rs1690816	C	0.31	0.02											
29292387	Neuroticism	b	rs1690816	C	0.31	0.02											
27089181	Depression	bc	rs1690818	T		5.41											
32438682	Childhood ALL/LBL (acute lymphoblastic leukemia/lymphoblastic lymphoma) treatment-related venous thromboembolism		rs17134658	?		5.55	G	0	-0.023	1.03	1.01	-0.001	-0.005	-0.050	-0.177	-0.002	0.98
30038396	Highest math class taken (MTAG)	b	rs17576589	T	0.34	0.01	T	0.32	0.027	1.01	1.01	0.000	0.008	-0.028	-0.086	0.000	0.99
30696823	Chronotype	c	rs17577073	A	0.56	1.02											
30595370	Morning person	b	rs17577073														

30804565	Insomnia	b	rs2406916	G	0.34	1.05	G	0.34	0.020	1.01	1.01	0.000	0.011	-0.029	-0.087	0.000	0.99
30643256	Well-being spectrum (multivariate analysis)	bc	rs2458167	C	0.69	0.01											
30643256	Neuroticism	bc	rs2458167	C	0.69	0.01											
30643256	Depressive symptoms	bc	rs2458167	C	0.69	0.01											
30643256	Life satisfaction	b	rs2458167	C	0.69	0.01											
30643256	Positive affect	b	rs2458167	C	0.69	0.01											
27089181	Neuroticism	b	rs2458167	A	0.30	0.02											
30072576	Blood protein levels		rs2614546	T	0.93	0.30	C	0.07	-0.011	1.00	1.00	0.000	0.006	0.182	0.248	0.001	1.03
29875488	Blood protein levels		rs4316475	A	0.92	0.39	G	0.07	-0.015	1.00	1.00	0.000	0.012	0.181	0.242	0.001	1.03
29942085	Depression	b	rs586533	T		6.03											
29292387	Depressive symptoms (MTAG)	b	rs586533	G	0.32	0.01											
30745170	Suicidality	b	rs598046	T	0.32	0.05											
29317604	Plasma kynurenine levels in major depressive disorder	a	rs61475845	A	0.02	2.20											
29500382	Feeling miserable	b	rs628246	T	0.17	5.49	T	0.17	0.033	0.99	0.99	0.000	0.001	0.014	-0.063	0.000	1.00
30804565	Insomnia	b	rs6589988	G	0.32	1.04	G	0.32	0.027	1.01	1.01	0.000	0.008	-0.029	-0.089	0.000	0.99
30595370	Systolic blood pressure	b	rs67885470														
32152699	Gastrointestinal infection	b	rs7112253	A		1.12	G	0.17	-0.013	1.00	1.01	0.000	0.026	-0.030	-0.116	0.000	1.03
32349335	Adipose morphology	a	rs72991567	G	0.08	67.00	G	0.10	-0.030	0.99	1.00	0.000	0.014	-0.132	-0.193	0.000	1.01
31289104	Gout vs. Hyperuricemia	c	rs7927466	A	0.95	1.85											

31596458	Spatial memory		rs79705697	A		0.36	A	0.03	-0.015	1.01	1.00	0.000	-0.039	0.045	-0.009	0.000	1.04
17903297	Volumetric brain MRI	a	rs952700				A	0.27	0.001	1.00	1.01	0.000	0.000	-0.068	-0.127	0.000	1.01
27863252	Plateletcrit	b	rs1111890	G	0.37	0.03											
28898252	Glycated hemoglobin levels	b	rs11224302	C	0.87	0.01	T	0.10	0.016	1.00	1.00	0.000	0.019	0.089	0.147	**-0.007**	1.03
23667675	Menarche (age at onset)	b	rs12800752	T	0.80	0.09											1.05
30019117	Adolescent idiopathic scoliosis	a	rs17095079														
26634245	Post bronchodilator FEV1/FVC ratio in COPD		rs1942108	A	0.49	0.01											
30738427	Metastasis in stage I-III microsatellite instability low/stable colorectal cancer (time to event)	a	rs4754687	A		13.33											
30595370	Lung function (FEV1/FVC)	b	rs594476				G	0.29	-0.019	1.00	1.01	0.000	-0.002	**0.135**	**0.227**	**0.002**	1.01
27863252	Red cell distribution width	b	rs717662	T	0.11	0.04	T	0.11	0.014	1.00	1.00	0.000	0.026	0.083	0.145	**-0.006**	1.02
28957414	Red cell distribution width	b	rs717662	T		0.05	T	0.11	0.014	1.00	1.00	0.000	0.026	0.083	0.145	**-0.006**	1.02
27863252	Reticulocyte count	b	rs72996113	T	0.10	0.05	T	0.10	0.017	1.00	1.00	0.000	0.018	0.084	0.150	**-0.007**	1.03
27863252	Reticulocyte fraction of red cells	b	rs72996113	T	0.10	0.05	T	0.10	0.017	1.00	1.00	0.000	0.018	0.084	0.150	**-0.007**	1.03
30595370	Red cell distribution width	b	rs72996119				G	0.10	0.017	1.00	1.00	0.000	0.018	0.089	0.144	**-0.007**	1.03
27989323	Interleukin-2 receptor antagonist levels		rs73001149	C		0.55	C	0.03	0.016	0.99	1.00	0.001	0.068	-0.008	-0.002	**-0.004**	1.02

30595370	White blood cell count	b		rs7934276				C	0.25	-0.028	1.00	1.01	0.000	-0.003	**0.189**	**0.301**	**0.002**	1.01
27863252	Lymphocyte counts	b		rs7939778	A	0.26	0.03											
25646338	Trans fatty acid levels	c		rs7952067	C	0.07	0.01	C	0.06	0.003	1.01	1.01	0.000	-0.008	-0.028	0.115	0.001	1.02
32888493	Platelet count	b		rs11224291	C	0.09	-0.03	C	0.08	0.017	1.00	1.00	0.000	0.039	0.031	0.062	**-0.007**	1.03
32888493	Platelet count	b		rs11224292	A	0.11		A	0.08	0.017	1.00	1.00	0.000	0.040	0.030	0.065	**-0.007**	1.04
32888493	Lymphocyte counts	b		rs11224302	T	0.11		T	0.10	0.016	1.00	1.00	0.000	0.019	0.089	0.147	**-0.007**	1.03
32888493	Lymphocyte counts	b		rs11224302	T	0.10	-0.04										**-0.007**	
32888493	Red cell distribution width	b		rs11224309	C	0.90		G	0.10	0.018	1.00	1.00	0.000	0.018	0.089	0.140	**-0.007**	1.03
32888493	Mean corpuscular volume	b		rs11224314	A	0.10	-0.03	A	0.10	0.018	1.00	1.00	0.000	0.021	0.079	0.141	**-0.007**	1.02
32888493	Mean corpuscular volume	b		rs11600132	C	0.11		C	0.10	0.019	1.00	1.00	0.000	0.020	0.083	0.143	**-0.007**	1.03
32117412	Carotid intima media thickness x smoking interaction			rs1939309	C		4.681 z score	C	0.40	0.002	0.99	1.00	0.000	-0.014	**0.216**	**0.362**	0.001	0.99
32888493	White blood cell count	b		rs7127313	T	0.33		T	0.4	-0.015	1.00	1.00	0.000	-0.005	**0.167**	**0.291**	**0.002**	1.01
32888493	White blood cell count	b		rs7127313	T	0.35	0.02											
32888493	Red cell distribution width	b		rs72996108	C	0.90	0.05											
32888493	Mean corpuscular hemoglobin concentration	b		rs72996113	T	0.10	0.04	T	0.10	0.017	1.00	1.00	0.000	0.018	0.084	0.150	**-0.007**	1.03
32888493	Mean corpuscular hemoglobin concentration	b		rs72996113	T	0.11												
27629369	Loneliness		CNTN6	rs116256240			0.17	C	0.02	0.011	1.03	1.00	0.000	-0.049	-0.158	-0.175	-0.001	1.01

30895295	Iris color (L* coordinate)	a	rs116546495	G	0.03	1.56	G	0.02	-0.008	0.99	0.98	0.000	-0.074	-0.025	-0.018	-0.001	0.99
28927378	Caudate activity during reward	a	rs139701434	A	0.02	2.54	A	0.02	0.000	1.01	1.00	0.000	0.016	-0.049	-0.099	0.000	1.02
28270201	Diastolic blood pressure	b	rs142892876	T	0.00	10.69											
28447399	Neurocognitive impairment in HIV-1 infection (continuous)		rs17038463	G		0.17	G	0.05	0.050	0.99	1.00	0.001	-0.040	-0.006	0.015	-0.001	0.94
27529621	Depressive symptoms (stressful life events interaction)	ac	rs17193334	A	0.07	0.11	A	0.05	-0.015	0.99	1.00	0.000	-0.022	-0.024	0.034	0.000	0.97
32572223	Gut microbiota (bacterial taxa, hurdle binary method)		rs3841919	C	0.01	-1.58	C	0.01	-0.047	0.98	0.97	0.000	-0.055	0.118	0.055	0.000	0.97
23823483	Metabolite levels		rs3852025			0.27	G	0.13	0.033	1.01	1.02	0.000	0.028	0.006	0.009	-0.001	0.99
23823483	Metabolite levels		rs7625523			0.19	A	0.25	0.020	1.02	1.01	0.000	0.010	0.002	-0.032	0.000	0.99
24324551	PR interval in Tripanosoma cruzi seropositivity	a	rs9815195														
22589738	Visceral adipose tissue/subcutaneous adipose tissue ratio	b	rs9861887	T	0.76												
31557306	Subjective response to placebo treatment in childhood asthma (change in cough/wheeze)	a	rs2729117	A	0.28	0.84											

24324551	PR interval in Tripanosoma cruzi seropositivity	a	rs76090503														
27723756	Gut microbiota (bacterial taxa)		rs11915634	T		1.30	C	0.05	-0.021	1.03	1.01	0.000	-0.005	-0.069	-0.013	0.000	1.01
27723756	Gut microbiota (bacterial taxa)		rs11915634	T		1.30	C	0.05	-0.021	1.03	1.01	0.000	-0.005	-0.069	-0.013	0.000	1.01
31519223	Gut microbiota (alpha diversity)	a	rs13082351			0.84											
31519223	Gut microbiota (alpha diversity)	a	rs13082351			0.49											
31519223	Gut microbiota (alpha diversity)	a	rs13082351			12.42											
23823483	Metabolite levels		rs17038979			0.42	C	0.08	-0.012	1.00	1.00	0.000	-0.009	-0.001	-0.036	0.000	0.98
30348214	DNA methylation variation (age effect)	a	rs192339095				A	0.03	-0.012	1.00	0.99	0.000	-0.018	-0.092	-0.222	-0.001	0.98
22675492	Estradiol levels		rs402675	A	0.50	0.10											
24871463	Systemic lupus erythematosus		rs4684256	T	0.28	1.48											

where: *, additional study information: a, discovery sample size <1000; b, discovery sample size >10,000; c, replication attempted; RAF, risk allele frequency; MAF minor allele frequency.

STable 6: Predicted effects of SNPs significantly associated with one or more trait.

SNP	Allele	Consequence	IMPACT	SYMBOL	BIOTYPE
rs3892248	A	intron_variant	MODIFIER	*NFASC*	protein_coding
rs4017874	G	intron_variant	MODIFIER	*NFASC*	protein_coding
rs7513016	G	upstream_gene_variant	MODIFIER	*CNTN2*	protein_coding
rs4951159	A	5_prime_UTR_variant	MODIFIER	*CNTN2*	protein_coding
rs3753847	C	intron_variant	MODIFIER	*CNTN2*	protein_coding
rs4951163	C	intron_variant	MODIFIER	*CNTN2*	protein_coding
rs1572995	T	intron_variant	MODIFIER	*CNTN2*	protein_coding
rs4951166	G	intron_variant	MODIFIER	*CNTN2*	protein_coding
rs3903399	C	intron_variant	MODIFIER	*CNTN2*	protein_coding
rs10900444	A	3_prime_UTR_variant	MODIFIER	*CNTN2*	protein_coding
rs9651227	G	downstream_gene_variant	MODIFIER	*RBBP5*	protein_coding
rs74787661	A	intron_variant	MODIFIER	*RBBP5*	protein_coding
rs12033493	T	intron_variant	MODIFIER	*RBBP5*	protein_coding
rs12022384	C	intron_variant	MODIFIER	*RBBP5*	protein_coding
rs7514499	C	intron_variant	MODIFIER	*RBBP5*	protein_coding
rs12027259	C	intron_variant	MODIFIER	*RBBP5*	protein_coding
rs754413251	A	intron_variant	MODIFIER	*RBBP5*	protein_coding
rs12025836	C	intron_variant	MODIFIER	*RBBP5*	protein_coding
rs61822618	G	intron_variant	MODIFIER	*RBBP5*	protein_coding
rs1339321	A	intron_variant	MODIFIER	*RBBP5*	protein_coding
rs3862948	A	upstream_gene_variant	MODIFIER	*AC093422.3*	processed_pseudogene
rs6593925	C	upstream_gene_variant	MODIFIER	*AC093422.3*	processed_pseudogene
rs61822621	A	upstream_gene_variant	MODIFIER	*AC093422.3*	processed_pseudogene
rs12039805	A	downstream_gene_variant	MODIFIER	*DSTYK*	protein_coding
rs7530217	A	downstream_gene_variant	MODIFIER	*DSTYK*	protein_coding
rs16855154	C	downstream_gene_variant	MODIFIER	*DSTYK*	protein_coding
rs3795556	C	3_prime_UTR_variant	MODIFIER	*DSTYK*	protein_coding
rs3183297	G	3_prime_UTR_variant	MODIFIER	*DSTYK*	protein_coding
rs1061132	A	3_prime_UTR_variant	MODIFIER	*DSTYK*	protein_coding

rs11808879	A	3_prime_UTR_variant	MODIFIER	*DSTYK*	protein_coding
rs16855186	C	intron_variant	MODIFIER	*DSTYK*	protein_coding
rs6664360	T	intron_variant	MODIFIER	*DSTYK*	protein_coding
rs34797971	CTTTCTTT	intron_variant	MODIFIER	*DSTYK*	protein_coding
rs61822627	A	intron_variant	MODIFIER	*DSTYK*	protein_coding
rs61822628	C	intron_variant	MODIFIER	*DSTYK*	protein_coding
rs12728202	A	intron_variant	MODIFIER	*DSTYK*	protein_coding
rs7541409	A	intron_variant	MODIFIER	*DSTYK*	protein_coding
rs7529665	A	intron_variant	MODIFIER	*DSTYK*	protein_coding
rs7538328	C	intron_variant	MODIFIER	*DSTYK*	protein_coding
rs1062715	A	synonymous_variant	LOW	*DSTYK*	protein_coding
rs3851294	G	missense_variant	MODERATE	*DSTYK*	protein_coding
rs386638726	GAC	intron_variant	MODIFIER	*DSTYK*	protein_coding
rs6684220	G	intron_variant	MODIFIER	*DSTYK*	protein_coding
rs11804620	A	intron_variant	MODIFIER	*DSTYK*	protein_coding
rs11811115	C	intron_variant	MODIFIER	*DSTYK*	protein_coding
rs12048416	C	intron_variant	MODIFIER	*DSTYK*	protein_coding
rs12048453	C	intron_variant	MODIFIER	*DSTYK*	protein_coding
rs4950984	G	intron_variant	MODIFIER	*DSTYK*	protein_coding
rs12023022	C	intron_variant	MODIFIER	*DSTYK*	protein_coding
rs10900458	G	intron_variant	MODIFIER	*DSTYK*	protein_coding
rs3851287	T	intron_variant	MODIFIER	*DSTYK*	protein_coding
rs3851286	G	intron_variant	MODIFIER	*DSTYK*	protein_coding
rs2369634	A	intron_variant	MODIFIER	*DSTYK*	protein_coding
rs6593930	T	intron_variant	MODIFIER	*DSTYK*	protein_coding
rs12078075	A	intron_variant	MODIFIER	*DSTYK*	protein_coding
rs6687271	C	intron_variant	MODIFIER	*DSTYK*	protein_coding
rs10494860	G	intron_variant	MODIFIER	*DSTYK*	protein_coding
rs10793726	T	intron_variant	MODIFIER	*DSTYK*	protein_coding
rs2369633	A	upstream_gene_variant	MODIFIER	*DSTYK*	protein_coding
rs10793727	G	intron_variant	MODIFIER	*TMCC2*	protein_coding

rs896319	T	intron_variant	MODIFIER	*TMCC2*	protein_coding
rs896322	G	intron_variant	MODIFIER	*TMCC2*	protein_coding
rs1668874	G	intron_variant	MODIFIER	*TMCC2*	protein_coding
rs9659978	C	intron_variant	MODIFIER	*TMCC2*	protein_coding
rs17728	C	3_prime_UTR_variant	MODIFIER	*TMCC2*	protein_coding
rs1172128	G	intron_variant,non_coding_transcript_variant	MODIFIER	*TMCC2*	processed_transcript
rs1172134	G	intron_variant,non_coding_transcript_variant	MODIFIER	*TMCC2*	processed_transcript
rs896321	G	downstream_gene_variant	MODIFIER	*TMCC2*	processed_transcript
rs1172122	C	intergenic_variant	MODIFIER	-	-
rs1172123	A	intergenic_variant	MODIFIER	-	-
rs373079331	AAAAAAAAAAA	intergenic_variant	MODIFIER	-	-
rs56323810	A	upstream_gene_variant	MODIFIER	*NUAK2*	protein_coding
rs55966786	A	regulatory_region_variant	MODIFIER	-	enhancer
rs149787271	T	intron_variant	MODIFIER	*CNTN5*	protein_coding
rs112365137	G	intron_variant	MODIFIER	*CNTN5*	protein_coding
rs113555997	G	intron_variant	MODIFIER	*CNTN5*	protein_coding
rs1452574	A	intron_variant	MODIFIER	*CNTN5*	protein_coding
rs1452575	A	intron_variant	MODIFIER	*CNTN5*	protein_coding
rs73003149	C	intergenic_variant	MODIFIER	-	-
rs117831801	C	intergenic_variant	MODIFIER	-	-
rs72992346	A	intergenic_variant	MODIFIER	-	-
rs72992349	G	intergenic_variant	MODIFIER	-	-
rs1939309	T	intergenic_variant	MODIFIER	-	-
rs1939308	A	intergenic_variant	MODIFIER	-	-
rs12280410	A	regulatory_region_variant	MODIFIER	-	enhancer
rs866903	A	intergenic_variant	MODIFIER	-	-
rs6590793	A	intergenic_variant	MODIFIER	-	-
rs199903594	TTTTTTTTTTT	intergenic_variant	MODIFIER	-	-
rs7102640	A	intergenic_variant	MODIFIER	-	-
rs147861539	A	intergenic_variant	MODIFIER	-	-
rs201835771	-	regulatory_region_variant	MODIFIER	-	promoter_flanking_region

rs72992361	A	regulatory_region_variant	MODIFIER	-	promoter_flanking_region
rs1502277	T	intergenic_variant	MODIFIER	-	-
rs6590794	G	intergenic_variant	MODIFIER	-	-
rs6590795	T	regulatory_region_variant	MODIFIER	-	enhancer
rs6590796	A	regulatory_region_variant	MODIFIER	-	enhancer
rs10894981	A	intergenic_variant	MODIFIER	-	-
rs72992370	T	intergenic_variant	MODIFIER	-	-
rs10791419	T	intergenic_variant	MODIFIER	-	-
rs1393354	A	intergenic_variant	MODIFIER	-	-
rs1393353	G	intergenic_variant	MODIFIER	-	-
rs11224277	T	intergenic_variant	MODIFIER	-	-
rs11224278	A	intergenic_variant	MODIFIER	-	-
rs962259	A	intergenic_variant	MODIFIER	-	-
rs962258	T	regulatory_region_variant	MODIFIER	-	promoter_flanking_region
rs7947335	A	regulatory_region_variant	MODIFIER	-	promoter_flanking_region
rs1502275	C	regulatory_region_variant	MODIFIER	-	promoter_flanking_region
rs10791420	G	intergenic_variant	MODIFIER	-	-
rs10791420	G	regulatory_region_variant	MODIFIER	-	promoter_flanking_region
rs4754688	C	regulatory_region_variant	MODIFIER	-	promoter_flanking_region
rs4754689	A	intergenic_variant	MODIFIER	-	-
rs4754690	C	intergenic_variant	MODIFIER	-	-
rs35165060	C	intergenic_variant	MODIFIER	-	-
rs10894983	G	intergenic_variant	MODIFIER	-	-
rs12365580	A	intergenic_variant	MODIFIER	-	-
rs35091610	T	intergenic_variant	MODIFIER	-	-
rs11224282	A	intergenic_variant	MODIFIER	-	-
rs146214587	TTTTTTT	intergenic_variant	MODIFIER	-	-
rs11224283	C	intergenic_variant	MODIFIER	-	-
rs11224284	A	intergenic_variant	MODIFIER	-	-
rs11224285	G	intergenic_variant	MODIFIER	-	-
rs66518530	T	intergenic_variant	MODIFIER	-	-

rs1216575	G	intergenic_variant	MODIFIER	-	-
rs1216576	A	intergenic_variant	MODIFIER	-	-
rs1216577	G	intergenic_variant	MODIFIER	-	-
rs11224286	G	intergenic_variant	MODIFIER	-	-
rs1216578	A	intergenic_variant	MODIFIER	-	-
rs2845930	G	intergenic_variant	MODIFIER	-	-
rs67786711	C	intergenic_variant	MODIFIER	-	-
rs10894984	T	intergenic_variant	MODIFIER	-	-
rs11224287	G	intergenic_variant	MODIFIER	-	-
rs1216579	T	intergenic_variant	MODIFIER	-	-
rs1216580	C	intergenic_variant	MODIFIER	-	-
rs1234614	C	intergenic_variant	MODIFIER	-	-
rs78355106	TTTTT	intergenic_variant	MODIFIER	-	-
rs11224289	A	intergenic_variant	MODIFIER	-	-
rs11224290	C	intergenic_variant	MODIFIER	-	-
rs744036	G	intergenic_variant	MODIFIER	-	-
rs11224291	C	regulatory_region_variant	MODIFIER	-	open_chromatin_region
rs7931511	A	regulatory_region_variant	MODIFIER	-	CTCF_binding_site
rs11224292	A	regulatory_region_variant	MODIFIER	-	CTCF_binding_site
rs1216528	A	intergenic_variant	MODIFIER	-	-
rs595239	G	intergenic_variant	MODIFIER	-	-
rs11224293	A	intergenic_variant	MODIFIER	-	-
rs650297	A	regulatory_region_variant	MODIFIER	-	enhancer
rs11224294	C	regulatory_region_variant	MODIFIER	-	enhancer
rs11224295	A	regulatory_region_variant	MODIFIER	-	enhancer
rs635541	A	regulatory_region_variant	MODIFIER	-	enhancer
rs146097009	AGAGAGAGAGA	intergenic_variant	MODIFIER	-	-
rs1939310	A	intergenic_variant	MODIFIER	-	-
rs66489236	C	intergenic_variant	MODIFIER	-	-
rs11224296	T	intergenic_variant	MODIFIER	-	-
rs11224297	A	intergenic_variant	MODIFIER	-	-

rs11600772	C	regulatory_region_variant	MODIFIER	-	promoter_flanking_region
rs11606531	T	regulatory_region_variant	MODIFIER	-	promoter_flanking_region
rs11224298	A	intergenic_variant	MODIFIER	-	-
rs72996113	T	intergenic_variant	MODIFIER	-	-
rs1783683	G	intergenic_variant	MODIFIER	-	-
rs11224302	G	intergenic_variant	MODIFIER	-	-
rs11224303	A	intergenic_variant	MODIFIER	-	-
rs11224304	T	intergenic_variant	MODIFIER	-	-
rs72996119	G	intergenic_variant	MODIFIER	-	-
rs3016199	A	intergenic_variant	MODIFIER	-	-
rs661134	G	intergenic_variant	MODIFIER	-	-
rs661210	C	intergenic_variant	MODIFIER	-	-
rs10894986	A	intergenic_variant	MODIFIER	-	-
rs11224306	A	intergenic_variant	MODIFIER	-	-
rs625505	G	intergenic_variant	MODIFIER	-	-
rs11224309	G	intergenic_variant	MODIFIER	-	-
rs72996129	C	intergenic_variant	MODIFIER	-	-
rs1216680	T	regulatory_region_variant	MODIFIER	-	open_chromatin_region
rs12223916	G	regulatory_region_variant	MODIFIER	-	open_chromatin_region
rs1216681	A	regulatory_region_variant	MODIFIER	-	open_chromatin_region
rs56821743	C	intergenic_variant	MODIFIER	-	-
rs61324436	C	intergenic_variant	MODIFIER	-	-
rs658072	C	intergenic_variant	MODIFIER	-	-
rs639494	C	intergenic_variant	MODIFIER	-	-
rs679014	T	intergenic_variant	MODIFIER	-	-
rs7105522	C	intergenic_variant	MODIFIER	-	-
rs7125960	A	intergenic_variant	MODIFIER	-	-
rs118190236	T	intergenic_variant	MODIFIER	-	-
rs1604519	G	intergenic_variant	MODIFIER	-	-
rs1502285	A	intergenic_variant	MODIFIER	-	-
rs10791421	G	intergenic_variant	MODIFIER	-	-

rs1145415	A	intergenic_variant	MODIFIER	-	-
rs11606890	C	intergenic_variant	MODIFIER	-	-
rs1216693	G	intergenic_variant	MODIFIER	-	-
rs11600132	A	intergenic_variant	MODIFIER	-	-
rs11601576	A	intergenic_variant	MODIFIER	-	-
rs143707862	CCCACCCACC	intergenic_variant	MODIFIER	-	-
rs756218807	C	intergenic_variant	MODIFIER	-	-
rs11224314	A	intergenic_variant	MODIFIER	-	-
rs80280902	G	intergenic_variant	MODIFIER	-	-
rs11530658	G	intergenic_variant	MODIFIER	-	-
rs76871092	A	intergenic_variant	MODIFIER	-	-
rs118100922	C	intergenic_variant	MODIFIER	-	-
rs11224317	A	intergenic_variant	MODIFIER	-	-
rs201152353	AAAAAAAAA	intergenic_variant	MODIFIER	-	-
rs112845240	A	intergenic_variant	MODIFIER	-	-
rs72998145	G	intergenic_variant	MODIFIER	-	-
rs117995162	G	intergenic_variant	MODIFIER	-	-
rs1145421	C	intergenic_variant	MODIFIER	-	-
rs12224813	T	intergenic_variant	MODIFIER	-	-
rs717662	T	intergenic_variant	MODIFIER	-	-
rs80135755	T	regulatory_region_variant	MODIFIER	-	enhancer
rs17095235	T	regulatory_region_variant	MODIFIER	-	enhancer
rs78194778	C	intergenic_variant	MODIFIER	-	-
rs74743669	T	intergenic_variant	MODIFIER	-	-
rs947867	G	intergenic_variant	MODIFIER	-	-
rs594476	C	intergenic_variant	MODIFIER	-	-
rs749433	T	intergenic_variant	MODIFIER	-	-
rs11224331	T	intergenic_variant	MODIFIER	-	-
rs675515	A	intergenic_variant	MODIFIER	-	-
rs76021287	C	regulatory_region_variant	MODIFIER	-	promoter_flanking_region
rs7934276	C	regulatory_region_variant	MODIFIER	-	promoter_flanking_region

rs11224334	G	intergenic_variant	MODIFIER	-	-
rs11224335	T	regulatory_region_variant	MODIFIER	-	promoter_flanking_region
rs11224336	A	regulatory_region_variant	MODIFIER	-	promoter_flanking_region
rs1502282	C	regulatory_region_variant	MODIFIER	-	promoter_flanking_region
rs11224337	A	regulatory_region_variant	MODIFIER	-	promoter_flanking_region
rs7944000	G	TF_binding_site_variant	MODIFIER	-	-
rs76578189	T	intergenic_variant	MODIFIER	-	-
rs7942811	A	intergenic_variant	MODIFIER	-	-
rs10894995	A	intergenic_variant	MODIFIER	-	-
rs4753978	C	downstream_gene_variant	MODIFIER	*RN7SL222P*	misc_RNA
rs11224340	A	downstream_gene_variant	MODIFIER	*RN7SL222P*	misc_RNA
rs4754691	T	downstream_gene_variant	MODIFIER	*RN7SL222P*	misc_RNA
rs11224342	C	downstream_gene_variant	MODIFIER	*RN7SL222P*	misc_RNA
rs10750591	G	downstream_gene_variant	MODIFIER	*RN7SL222P*	misc_RNA
rs35469123	TAT	downstream_gene_variant	MODIFIER	*RN7SL222P*	misc_RNA
rs916482	C	downstream_gene_variant	MODIFIER	*RN7SL222P*	misc_RNA
rs7932493	A	downstream_gene_variant	MODIFIER	*RN7SL222P*	misc_RNA
rs1032778	C	downstream_gene_variant	MODIFIER	*RN7SL222P*	misc_RNA
rs11224347	A	non_coding_transcript_exon_variant	MODIFIER	*RN7SL222P*	misc_RNA
rs11224348	T	upstream_gene_variant	MODIFIER	*RN7SL222P*	misc_RNA
rs75986749	C	upstream_gene_variant	MODIFIER	*RN7SL222P*	misc_RNA
rs74788811	A	upstream_gene_variant	MODIFIER	*RN7SL222P*	misc_RNA
rs9888185	G	upstream_gene_variant	MODIFIER	*RN7SL222P*	misc_RNA
rs11224350	A	upstream_gene_variant	MODIFIER	*RN7SL222P*	misc_RNA
rs11224350	C	upstream_gene_variant	MODIFIER	*RN7SL222P*	misc_RNA
rs1032779	C	upstream_gene_variant	MODIFIER	*RN7SL222P*	misc_RNA
rs11224351	C	upstream_gene_variant	MODIFIER	*RN7SL222P*	misc_RNA
rs2000520	A	upstream_gene_variant	MODIFIER	*RN7SL222P*	misc_RNA
rs10791423	C	upstream_gene_variant	MODIFIER	*RN7SL222P*	misc_RNA
rs7480623	G	upstream_gene_variant	MODIFIER	*RN7SL222P*	misc_RNA
rs1587647	A	upstream_gene_variant	MODIFIER	*RN7SL222P*	misc_RNA

rs11607909	T	upstream_gene_variant	MODIFIER	*RN7SL222P*	misc_RNA
rs7934743	T	upstream_gene_variant	MODIFIER	*RN7SL222P*	misc_RNA
rs11224353	A	upstream_gene_variant	MODIFIER	*RN7SL222P*	misc_RNA
rs7924327	A	upstream_gene_variant	MODIFIER	*RN7SL222P*	misc_RNA
rs7934207	A	upstream_gene_variant	MODIFIER	*RN7SL222P*	misc_RNA
rs7935202	G	intergenic_variant	MODIFIER	-	-
rs10894997	A	intergenic_variant	MODIFIER	-	-
rs1938907	C	intergenic_variant	MODIFIER	-	-
rs55657437	A	regulatory_region_variant	MODIFIER	-	enhancer
rs71476638	A	intergenic_variant	MODIFIER	-	-
rs79174253	C	intergenic_variant	MODIFIER	-	-
rs75760535	A	intergenic_variant	MODIFIER	-	-
rs12221896	T	intergenic_variant	MODIFIER	-	-
rs11224354	A	intergenic_variant	MODIFIER	-	-
rs11224356	A	intergenic_variant	MODIFIER	-	-
rs12363256	A	intergenic_variant	MODIFIER	-	-
rs11224357	A	intergenic_variant	MODIFIER	-	-
rs11224358	G	intergenic_variant	MODIFIER	-	-
rs12361534	A	intergenic_variant	MODIFIER	-	-
rs77132106	TTT	intergenic_variant	MODIFIER	-	-
rs12362972	C	intergenic_variant	MODIFIER	-	-
rs11224359	C	intergenic_variant	MODIFIER	-	-
rs11224359	G	intergenic_variant	MODIFIER	-	-
rs11535737	A	intergenic_variant	MODIFIER	-	-
rs35693891	A	intergenic_variant	MODIFIER	-	-
rs1938906	A	intergenic_variant	MODIFIER	-	-
rs2088952	G	intergenic_variant	MODIFIER	-	-
rs61908725	A	intergenic_variant	MODIFIER	-	-
rs11224360	C	intergenic_variant	MODIFIER	-	-
rs2407694	C	intergenic_variant	MODIFIER	-	-
rs7941473	G	intergenic_variant	MODIFIER	-	-

rs2105582	G	intergenic_variant	MODIFIER	-	-
rs4272757	A	intergenic_variant	MODIFIER	-	-
rs11530779	T	intergenic_variant	MODIFIER	-	-
rs7480411	A	intergenic_variant	MODIFIER	-	-
rs7480600	T	intergenic_variant	MODIFIER	-	-
rs568696696	C	intergenic_variant	MODIFIER	-	-
rs375200975	C	intergenic_variant	MODIFIER	-	-
rs7484139	G	intergenic_variant	MODIFIER	-	-
rs2096710	G	intergenic_variant	MODIFIER	-	-
rs4285842	A	intergenic_variant	MODIFIER	-	-
rs9711476	A	intergenic_variant	MODIFIER	-	-
rs11224361	C	intergenic_variant	MODIFIER	-	-
rs11224362	A	intergenic_variant	MODIFIER	-	-
rs11224363	T	intergenic_variant	MODIFIER	-	-
rs4347357	C	intergenic_variant	MODIFIER	-	-
rs4340009	A	intergenic_variant	MODIFIER	-	-
rs79853957	G	intergenic_variant	MODIFIER	-	-
rs75633940	A	intergenic_variant	MODIFIER	-	-
rs36195752	G	intergenic_variant	MODIFIER	-	-
rs11500929	C	intergenic_variant	MODIFIER	-	-
rs2134303	G	intergenic_variant	MODIFIER	-	-
rs11224366	G	intergenic_variant	MODIFIER	-	-
rs78192924	A	intergenic_variant	MODIFIER	-	-
rs7111819	G	upstream_gene_variant	MODIFIER	*PPIAP43*	processed_pseudogene
rs10750593	G	upstream_gene_variant	MODIFIER	*PPIAP43*	processed_pseudogene
rs7127830	C	upstream_gene_variant	MODIFIER	*PPIAP43*	processed_pseudogene
rs4753979	A	upstream_gene_variant	MODIFIER	*PPIAP43*	processed_pseudogene
rs7117419	C	upstream_gene_variant	MODIFIER	*PPIAP43*	processed_pseudogene
rs7942556	C	upstream_gene_variant	MODIFIER	*PPIAP43*	processed_pseudogene
rs11224367	G	upstream_gene_variant	MODIFIER	*PPIAP43*	processed_pseudogene
rs11224368	A	upstream_gene_variant	MODIFIER	*PPIAP43*	processed_pseudogene

rs79152974	G	non_coding_transcript_exon_variant	MODIFIER	*PPIAP43*	processed_pseudogene
rs11224369	A	downstream_gene_variant	MODIFIER	*PPIAP43*	processed_pseudogene
rs12221817	A	downstream_gene_variant	MODIFIER	*PPIAP43*	processed_pseudogene
rs11224372	A	downstream_gene_variant	MODIFIER	*PPIAP43*	processed_pseudogene
rs17095329	A	downstream_gene_variant	MODIFIER	*PPIAP43*	processed_pseudogene
rs3958711	G	downstream_gene_variant	MODIFIER	*PPIAP43*	processed_pseudogene
rs924647	G	downstream_gene_variant	MODIFIER	*PPIAP43*	processed_pseudogene
rs7350545	G	intergenic_variant	MODIFIER	-	-
rs6590804	A	regulatory_region_variant	MODIFIER	-	open_chromatin_region
rs10431107	C	intergenic_variant	MODIFIER	-	-
rs10160451	T	regulatory_region_variant	MODIFIER	-	TF_binding_site
rs6590805	T	intergenic_variant	MODIFIER	-	-
rs61908735	G	intergenic_variant	MODIFIER	-	-
rs34324928	T	intergenic_variant	MODIFIER	-	-
rs4753980	T	regulatory_region_variant	MODIFIER	-	promoter_flanking_region
rs756756606	GACGAC	regulatory_region_variant	MODIFIER	-	promoter_flanking_region
rs11224376	A	TF_binding_site_variant	MODIFIER	-	-
rs79398819	A	intergenic_variant	MODIFIER	-	-
rs201751360	T	intergenic_variant	MODIFIER	-	-
rs61908736	G	regulatory_region_variant	MODIFIER	-	CTCF_binding_site
rs11224377	A	regulatory_region_variant	MODIFIER	-	CTCF_binding_site
rs201901540	TTTTTTTTTTT	intergenic_variant	MODIFIER	-	-
rs10895006	T	intergenic_variant	MODIFIER	-	-
rs4754692	G	intergenic_variant	MODIFIER	-	-
rs145548665	A	intergenic_variant	MODIFIER	-	-
rs6590806	A	downstream_gene_variant	MODIFIER	*AP001351.1*	lncRNA
rs10895007	C	downstream_gene_variant	MODIFIER	*AP001351.1*	lncRNA
rs11224378	C	downstream_gene_variant	MODIFIER	*AP001351.1*	lncRNA
rs10160533	A	downstream_gene_variant	MODIFIER	*AP001351.1*	lncRNA
rs10160326	T	downstream_gene_variant	MODIFIER	*AP001351.1*	lncRNA
rs151206225	G	downstream_gene_variant	MODIFIER	*AP001351.1*	lncRNA

rs11224379	A	downstream_gene_variant	MODIFIER	*AP001351.1*	lncRNA
rs4754693	A	downstream_gene_variant	MODIFIER	*AP001351.1*	lncRNA
rs4754694	A	downstream_gene_variant	MODIFIER	*AP001351.1*	lncRNA
rs11224380	A	downstream_gene_variant	MODIFIER	*AP001351.1*	lncRNA
rs11224381	A	downstream_gene_variant	MODIFIER	*AP001351.1*	lncRNA
rs79124516	T	downstream_gene_variant	MODIFIER	*AP001351.1*	lncRNA
rs11224382	G	downstream_gene_variant	MODIFIER	*AP001351.1*	lncRNA
rs10160586	C	downstream_gene_variant	MODIFIER	*AP001351.1*	lncRNA
rs10160408	T	downstream_gene_variant	MODIFIER	*AP001351.1*	lncRNA
rs10160729	A	upstream_gene_variant	MODIFIER	*ARHGAP42*	protein_coding
rs6590807	A	upstream_gene_variant	MODIFIER	*ARHGAP42*	protein_coding
rs11224383	G	upstream_gene_variant	MODIFIER	*ARHGAP42*	protein_coding
rs7107791	A	upstream_gene_variant	MODIFIER	*ARHGAP42*	protein_coding
rs11224384	T	upstream_gene_variant	MODIFIER	*ARHGAP42*	protein_coding
rs1021794	A	upstream_gene_variant	MODIFIER	*ARHGAP42*	protein_coding
rs61908737	A	non_coding_transcript_exon_variant	MODIFIER	*AP001351.1*	lncRNA
rs10895008	C	non_coding_transcript_exon_variant	MODIFIER	*AP001351.1*	lncRNA
rs11224387	C	non_coding_transcript_exon_variant	MODIFIER	*AP001351.1*	lncRNA
rs61908738	C	non_coding_transcript_exon_variant	MODIFIER	*AP001351.1*	lncRNA
rs7938601	T	non_coding_transcript_exon_variant	MODIFIER	*AP001351.1*	lncRNA
rs11373240	TTTTTTT	non_coding_transcript_exon_variant	MODIFIER	*AP001351.1*	lncRNA
rs76715013	C	intron_variant,non_coding_transcript_variant	MODIFIER	*AP001351.1*	lncRNA
rs7928871	T	intron_variant,non_coding_transcript_variant	MODIFIER	*AP001351.1*	lncRNA
rs7929942	G	intron_variant,non_coding_transcript_variant	MODIFIER	*AP001351.1*	lncRNA
rs12280935	C	intron_variant,non_coding_transcript_variant	MODIFIER	*AP001351.1*	lncRNA
rs61908739	C	intron_variant,non_coding_transcript_variant	MODIFIER	*AP001351.1*	lncRNA
rs77786711	G	intron_variant	MODIFIER	*ARHGAP42*	protein_coding
rs201445134	TTTTTTTTTT	intron_variant	MODIFIER	*ARHGAP42*	protein_coding
rs11224389	A	intron_variant	MODIFIER	*ARHGAP42*	protein_coding
rs77802037	C	intron_variant	MODIFIER	*ARHGAP42*	protein_coding
rs6590808	T	intron_variant	MODIFIER	*ARHGAP42*	protein_coding

rs61908740	A	intron_variant	MODIFIER	*ARHGAP42*	protein_coding
rs61908741	T	intron_variant	MODIFIER	*ARHGAP42*	protein_coding
rs151184227	G	intron_variant	MODIFIER	*ARHGAP42*	protein_coding
rs61472500	A	intron_variant	MODIFIER	*ARHGAP42*	protein_coding
rs71476642	T	intron_variant	MODIFIER	*ARHGAP42*	protein_coding
rs61908744	G	intron_variant	MODIFIER	*ARHGAP42*	protein_coding
rs61908745	A	intron_variant	MODIFIER	*ARHGAP42*	protein_coding
rs7947643	G	intron_variant	MODIFIER	*ARHGAP42*	protein_coding
rs11224390	T	intron_variant	MODIFIER	*ARHGAP42*	protein_coding
rs17712139	G	intron_variant	MODIFIER	*ARHGAP42*	protein_coding
rs7952198	A	intron_variant	MODIFIER	*ARHGAP42*	protein_coding
rs78901113	A	intron_variant	MODIFIER	*ARHGAP42*	protein_coding
rs11224393	G	intron_variant	MODIFIER	*ARHGAP42*	protein_coding
rs73001993	G	intron_variant	MODIFIER	*ARHGAP42*	protein_coding
rs77474430	AA	intron_variant	MODIFIER	*ARHGAP42*	protein_coding
rs61621482	G	intron_variant	MODIFIER	*ARHGAP42*	protein_coding
rs57637131	A	intron_variant	MODIFIER	*ARHGAP42*	protein_coding
rs7129917	G	intron_variant	MODIFIER	*ARHGAP42*	protein_coding
rs73573308	G	intron_variant	MODIFIER	*ARHGAP42*	protein_coding
rs34225168	A	intron_variant	MODIFIER	*ARHGAP42*	protein_coding
rs1216744	T	intron_variant	MODIFIER	*ARHGAP42*	protein_coding
rs750534145	T	intron_variant	MODIFIER	*ARHGAP42*	protein_coding
rs76151625	C	intron_variant	MODIFIER	*ARHGAP42*	protein_coding
rs7938194	G	intron_variant	MODIFIER	*ARHGAP42*	protein_coding
rs12278242	A	intron_variant	MODIFIER	*ARHGAP42*	protein_coding
rs12291857	A	intron_variant	MODIFIER	*ARHGAP42*	protein_coding
rs201584625	AAAAAA	intron_variant	MODIFIER	*ARHGAP42*	protein_coding
rs754232464	GTGAGCCTG	intron_variant	MODIFIER	*ARHGAP42*	protein_coding
rs34296918	G	intron_variant	MODIFIER	*ARHGAP42*	protein_coding
rs10431109	A	intron_variant	MODIFIER	*ARHGAP42*	protein_coding
rs11224400	A	intron_variant	MODIFIER	*ARHGAP42*	protein_coding

rs6590810	G	intron_variant	MODIFIER	*ARHGAP42*	protein_coding
rs11224402	C	intron_variant	MODIFIER	*ARHGAP42*	protein_coding
rs150036452	A	intron_variant	MODIFIER	*ARHGAP42*	protein_coding
rs61910470	A	intron_variant	MODIFIER	*ARHGAP42*	protein_coding
rs6590812	A	intron_variant	MODIFIER	*ARHGAP42*	protein_coding
rs12295008	A	intron_variant	MODIFIER	*ARHGAP42*	protein_coding
rs2298609	G	intron_variant	MODIFIER	*ARHGAP42*	protein_coding
rs2298610	G	intron_variant	MODIFIER	*ARHGAP42*	protein_coding
rs2298611	A	intron_variant	MODIFIER	*ARHGAP42*	protein_coding
rs1502284	A	intron_variant	MODIFIER	*ARHGAP42*	protein_coding
rs73003836	T	intron_variant	MODIFIER	*ARHGAP42*	protein_coding
rs7945656	A	intron_variant	MODIFIER	*ARHGAP42*	protein_coding
rs7106603	A	intron_variant	MODIFIER	*ARHGAP42*	protein_coding
rs143012079	TTTTT	intron_variant	MODIFIER	*ARHGAP42*	protein_coding
rs61910471	T	intron_variant	MODIFIER	*ARHGAP42*	protein_coding
rs11224405	G	intron_variant	MODIFIER	*ARHGAP42*	protein_coding
rs61910472	T	intron_variant	MODIFIER	*ARHGAP42*	protein_coding
rs61910473	G	intron_variant	MODIFIER	*ARHGAP42*	protein_coding
rs7926526	A	intron_variant	MODIFIER	*ARHGAP42*	protein_coding
rs11224406	A	intron_variant	MODIFIER	*ARHGAP42*	protein_coding
rs74997273	G	intron_variant	MODIFIER	*ARHGAP42*	protein_coding
rs61910474	A	intron_variant	MODIFIER	*ARHGAP42*	protein_coding
rs61910475	G	intron_variant	MODIFIER	*ARHGAP42*	protein_coding
rs1502283	G	intron_variant	MODIFIER	*ARHGAP42*	protein_coding
rs6590814	C	intron_variant	MODIFIER	*ARHGAP42*	protein_coding
rs7944469	T	intron_variant	MODIFIER	*ARHGAP42*	protein_coding
rs7945336	T	intron_variant	MODIFIER	*ARHGAP42*	protein_coding
rs117594293	T	intron_variant	MODIFIER	*ARHGAP42*	protein_coding
rs117311034	T	intron_variant	MODIFIER	*ARHGAP42*	protein_coding
rs11224407	T	intron_variant	MODIFIER	*ARHGAP42*	protein_coding
rs11224408	A	intron_variant	MODIFIER	*ARHGAP42*	protein_coding

rs61910477	T	intron_variant	MODIFIER	*ARHGAP42*	protein_coding
rs12284151	C	intron_variant	MODIFIER	*ARHGAP42*	protein_coding
rs552114751	TTTTTTTTTTT	intron_variant	MODIFIER	*ARHGAP42*	protein_coding
rs61910478	T	intron_variant	MODIFIER	*ARHGAP42*	protein_coding
rs4753982	C	intron_variant	MODIFIER	*ARHGAP42*	protein_coding
rs4754696	C	intron_variant	MODIFIER	*ARHGAP42*	protein_coding
rs4753983	C	intron_variant	MODIFIER	*ARHGAP42*	protein_coding
rs4753984	A	intron_variant	MODIFIER	*ARHGAP42*	protein_coding
rs61910479	T	intron_variant	MODIFIER	*ARHGAP42*	protein_coding
rs7105451	A	intron_variant	MODIFIER	*ARHGAP42*	protein_coding
rs61910480	G	intron_variant	MODIFIER	*ARHGAP42*	protein_coding
rs633185	A	intron_variant	MODIFIER	*ARHGAP42*	protein_coding
rs74594440	T	intron_variant	MODIFIER	*ARHGAP42*	protein_coding
rs11224411	A	intron_variant	MODIFIER	*ARHGAP42*	protein_coding
rs10128687	G	intron_variant	MODIFIER	*ARHGAP42*	protein_coding
rs7130275	C	intron_variant	MODIFIER	*ARHGAP42*	protein_coding
rs7103397	C	intron_variant	MODIFIER	*ARHGAP42*	protein_coding
rs7114867	A	intron_variant	MODIFIER	*ARHGAP42*	protein_coding
rs7126604	G	intron_variant	MODIFIER	*ARHGAP42*	protein_coding
rs7112041	C	intron_variant	MODIFIER	*ARHGAP42*	protein_coding
rs76163145	T	intron_variant	MODIFIER	*ARHGAP42*	protein_coding
rs11224412	T	intron_variant	MODIFIER	*ARHGAP42*	protein_coding
rs11224413	A	intron_variant	MODIFIER	*ARHGAP42*	protein_coding
rs7952055	A	intron_variant	MODIFIER	*ARHGAP42*	protein_coding
rs7928576	T	intron_variant	MODIFIER	*ARHGAP42*	protein_coding
rs79571719	A	intron_variant	MODIFIER	*ARHGAP42*	protein_coding
rs139591012	CACTCACT	intron_variant	MODIFIER	*ARHGAP42*	protein_coding
rs7126015	G	intron_variant	MODIFIER	*ARHGAP42*	protein_coding
rs61910503	A	intron_variant	MODIFIER	*ARHGAP42*	protein_coding
rs117695557	G	intron_variant	MODIFIER	*ARHGAP42*	protein_coding
rs12291493	G	intron_variant	MODIFIER	*ARHGAP42*	protein_coding

rs12286062	T	intron_variant	MODIFIER	*ARHGAP42*	protein_coding
rs34025953	G	intron_variant	MODIFIER	*ARHGAP42*	protein_coding
rs10895011	A	intron_variant	MODIFIER	*ARHGAP42*	protein_coding
rs11224417	C	intron_variant	MODIFIER	*ARHGAP42*	protein_coding
rs6590817	A	intron_variant	MODIFIER	*ARHGAP42*	protein_coding
rs7946913	A	intron_variant	MODIFIER	*ARHGAP42*	protein_coding
rs78894707	A	intron_variant	MODIFIER	*ARHGAP42*	protein_coding
rs77431356	T	intron_variant	MODIFIER	*ARHGAP42*	protein_coding
rs117241374	C	intron_variant	MODIFIER	*ARHGAP42*	protein_coding
rs6590818	C	intron_variant	MODIFIER	*ARHGAP42*	protein_coding
rs150999923	CCCC	intron_variant	MODIFIER	*ARHGAP42*	protein_coding
rs141913306	TTTTT	intron_variant	MODIFIER	*ARHGAP42*	protein_coding
rs7930157	G	intron_variant	MODIFIER	*ARHGAP42*	protein_coding
rs61910504	G	intron_variant	MODIFIER	*ARHGAP42*	protein_coding
rs61910505	G	intron_variant	MODIFIER	*ARHGAP42*	protein_coding
rs10686612	TGT	intron_variant	MODIFIER	*ARHGAP42*	protein_coding
rs80276828	C	intron_variant	MODIFIER	*ARHGAP42*	protein_coding
rs7120147	C	intron_variant	MODIFIER	*ARHGAP42*	protein_coding
rs142374585	GTGCGTG	intron_variant	MODIFIER	*ARHGAP42*	protein_coding
rs11224426	A	intron_variant	MODIFIER	*ARHGAP42*	protein_coding
rs4754697	A	intron_variant	MODIFIER	*ARHGAP42*	protein_coding
rs115034314	C	intron_variant	MODIFIER	*ARHGAP42*	protein_coding
rs75695106	A	intron_variant	MODIFIER	*ARHGAP42*	protein_coding
rs11824393	T	intron_variant	MODIFIER	*ARHGAP42*	protein_coding
rs11824432	A	intron_variant	MODIFIER	*ARHGAP42*	protein_coding
rs73005719	C	intron_variant	MODIFIER	*ARHGAP42*	protein_coding
rs61910508	T	intron_variant	MODIFIER	*ARHGAP42*	protein_coding
rs80333418	A	intron_variant	MODIFIER	*ARHGAP42*	protein_coding
rs12223958	C	intron_variant	MODIFIER	*ARHGAP42*	protein_coding
rs4087602	G	intron_variant	MODIFIER	*ARHGAP42*	protein_coding
rs201443872	TTTTTTTTTT	intron_variant	MODIFIER	*ARHGAP42*	protein_coding

rs7117515	G	intron_variant	MODIFIER	*ARHGAP42*	protein_coding
rs4337001	G	intron_variant	MODIFIER	*ARHGAP42*	protein_coding
rs2088953	A	intron_variant	MODIFIER	*ARHGAP42*	protein_coding
rs2407750	A	intron_variant	MODIFIER	*ARHGAP42*	protein_coding
rs11224430	G	intron_variant	MODIFIER	*ARHGAP42*	protein_coding
rs73005729	C	intron_variant	MODIFIER	*ARHGAP42*	protein_coding
rs61892334	A	intron_variant	MODIFIER	*ARHGAP42*	protein_coding
rs7947761	G	intron_variant	MODIFIER	*ARHGAP42*	protein_coding
rs78737960	G	intron_variant	MODIFIER	*ARHGAP42*	protein_coding
rs7116153	A	intron_variant	MODIFIER	*ARHGAP42*	protein_coding
rs7130855	T	intron_variant	MODIFIER	*ARHGAP42*	protein_coding
rs4495864	C	intron_variant	MODIFIER	*ARHGAP42*	protein_coding
rs17095405	G	intron_variant	MODIFIER	*ARHGAP42*	protein_coding
rs61892352	A	intron_variant	MODIFIER	*ARHGAP42*	protein_coding
rs58727919	G	intron_variant	MODIFIER	*ARHGAP42*	protein_coding
rs12226782	A	intron_variant	MODIFIER	*ARHGAP42*	protein_coding
rs61892355	G	intron_variant	MODIFIER	*ARHGAP42*	protein_coding
rs9919648	G	intron_variant	MODIFIER	*ARHGAP42*	protein_coding
rs4754698	A	intron_variant	MODIFIER	*ARHGAP42*	protein_coding
rs12221887	T	intron_variant	MODIFIER	*ARHGAP42*	protein_coding
rs61890760	A	intron_variant	MODIFIER	*ARHGAP42*	protein_coding
rs17095409	C	intron_variant	MODIFIER	*ARHGAP42*	protein_coding
rs12225108	T	intron_variant	MODIFIER	*ARHGAP42*	protein_coding
rs7126324	T	intron_variant	MODIFIER	*ARHGAP42*	protein_coding
rs7125452	A	intron_variant	MODIFIER	*ARHGAP42*	protein_coding
rs4469852	C	intron_variant	MODIFIER	*ARHGAP42*	protein_coding
rs11224435	G	intron_variant	MODIFIER	*ARHGAP42*	protein_coding
rs60708905	G	intron_variant	MODIFIER	*ARHGAP42*	protein_coding
rs4517481	G	intron_variant	MODIFIER	*ARHGAP42*	protein_coding
rs11224436	T	intron_variant	MODIFIER	*ARHGAP42*	protein_coding
rs34636774	G	intron_variant	MODIFIER	*ARHGAP42*	protein_coding

rs11224437	C	intron_variant	MODIFIER	*ARHGAP42*	protein_coding
rs12792556	T	intron_variant	MODIFIER	*ARHGAP42*	protein_coding
rs7942588	G	intron_variant	MODIFIER	*ARHGAP42*	protein_coding
rs7927197	T	intron_variant	MODIFIER	*ARHGAP42*	protein_coding
rs492237	C	intron_variant	MODIFIER	*ARHGAP42*	protein_coding
rs7105842	C	intron_variant	MODIFIER	*ARHGAP42*	protein_coding
rs7120712	A	intron_variant	MODIFIER	*ARHGAP42*	protein_coding
rs17713163	G	intron_variant	MODIFIER	*ARHGAP42*	protein_coding
rs17095426	A	intron_variant	MODIFIER	*ARHGAP42*	protein_coding
rs17095428	C	intron_variant	MODIFIER	*ARHGAP42*	protein_coding
rs140748271	AA	intron_variant	MODIFIER	*ARHGAP42*	protein_coding
rs61890763	A	intron_variant	MODIFIER	*ARHGAP42*	protein_coding
rs61890764	T	intron_variant	MODIFIER	*ARHGAP42*	protein_coding
rs17095429	A	intron_variant	MODIFIER	*ARHGAP42*	protein_coding
rs144709517	G	intron_variant	MODIFIER	*ARHGAP42*	protein_coding
rs3901516	A	intron_variant	MODIFIER	*ARHGAP42*	protein_coding
rs7102634	A	intron_variant	MODIFIER	*ARHGAP42*	protein_coding
rs693080	C	intron_variant	MODIFIER	*ARHGAP42*	protein_coding
rs17095436	T	intron_variant	MODIFIER	*ARHGAP42*	protein_coding
rs7944598	T	intron_variant	MODIFIER	*ARHGAP42*	protein_coding
rs79336312	T	intron_variant	MODIFIER	*ARHGAP42*	protein_coding
rs35132040	TTTTTTT	intron_variant	MODIFIER	*ARHGAP42*	protein_coding
rs17095438	A	intron_variant	MODIFIER	*ARHGAP42*	protein_coding
rs17095439	C	intron_variant	MODIFIER	*ARHGAP42*	protein_coding
rs7110872	A	intron_variant	MODIFIER	*ARHGAP42*	protein_coding
rs598369	T	intron_variant	MODIFIER	*ARHGAP42*	protein_coding
rs7112228	T	intron_variant	MODIFIER	*ARHGAP42*	protein_coding
rs1144128	G	intron_variant	MODIFIER	*ARHGAP42*	protein_coding
rs11224442	T	intron_variant	MODIFIER	*ARHGAP42*	protein_coding
rs7112366	A	intron_variant	MODIFIER	*ARHGAP42*	protein_coding
rs61890765	G	intron_variant	MODIFIER	*ARHGAP42*	protein_coding

rs4121392	T	intron_variant	MODIFIER	*ARHGAP42*	protein_coding
rs73007637	T	intron_variant	MODIFIER	*ARHGAP42*	protein_coding
rs6590820	G	intron_variant	MODIFIER	*ARHGAP42*	protein_coding
rs625761	A	intron_variant	MODIFIER	*ARHGAP42*	protein_coding
rs11224443	C	intron_variant	MODIFIER	*ARHGAP42*	protein_coding
rs7123718	C	intron_variant	MODIFIER	*ARHGAP42*	protein_coding
rs499241	T	intron_variant	MODIFIER	*ARHGAP42*	protein_coding
rs60799929	A	intron_variant	MODIFIER	*ARHGAP42*	protein_coding
rs682847	C	intron_variant	MODIFIER	*ARHGAP42*	protein_coding
rs686056	A	intron_variant	MODIFIER	*ARHGAP42*	protein_coding
rs507695	T	intron_variant	MODIFIER	*ARHGAP42*	protein_coding
rs655922	C	intron_variant	MODIFIER	*ARHGAP42*	protein_coding
rs148274695	G	intron_variant	MODIFIER	*ARHGAP42*	protein_coding
rs75057863	G	intron_variant	MODIFIER	*ARHGAP42*	protein_coding
rs73007642	T	intron_variant	MODIFIER	*ARHGAP42*	protein_coding
rs1867063	A	intron_variant	MODIFIER	*ARHGAP42*	protein_coding
rs58051655	A	intron_variant	MODIFIER	*ARHGAP42*	protein_coding
rs588615	A	intron_variant	MODIFIER	*ARHGAP42*	protein_coding
rs183926634	A	intron_variant	MODIFIER	*ARHGAP42*	protein_coding
rs139790431	C	intron_variant	MODIFIER	*ARHGAP42*	protein_coding
rs612090	C	intron_variant	MODIFIER	*ARHGAP42*	protein_coding
rs595030	G	intron_variant	MODIFIER	*ARHGAP42*	protein_coding
rs73007646	T	intron_variant	MODIFIER	*ARHGAP42*	protein_coding
rs77797324	G	intron_variant	MODIFIER	*ARHGAP42*	protein_coding
rs79661065	AA	intron_variant	MODIFIER	*ARHGAP42*	protein_coding
rs660736	G	intron_variant	MODIFIER	*ARHGAP42*	protein_coding
rs626558	C	intron_variant	MODIFIER	*ARHGAP42*	protein_coding
rs536090	A	intron_variant	MODIFIER	*ARHGAP42*	protein_coding
rs509358	T	intron_variant	MODIFIER	*ARHGAP42*	protein_coding
rs71476649	C	intron_variant	MODIFIER	*ARHGAP42*	protein_coding
rs2407752	G	intron_variant	MODIFIER	*ARHGAP42*	protein_coding

rs685611	C	intron_variant	MODIFIER	*ARHGAP42*	protein_coding
rs600906	C	intron_variant	MODIFIER	*ARHGAP42*	protein_coding
rs600453	G	intron_variant	MODIFIER	*ARHGAP42*	protein_coding
rs600372	C	intron_variant	MODIFIER	*ARHGAP42*	protein_coding
rs76282170	CCC	intron_variant	MODIFIER	*ARHGAP42*	protein_coding
rs61890770	A	intron_variant	MODIFIER	*ARHGAP42*	protein_coding
rs11224446	C	intron_variant	MODIFIER	*ARHGAP42*	protein_coding
rs582409	C	intron_variant	MODIFIER	*ARHGAP42*	protein_coding
rs607890	A	intron_variant	MODIFIER	*ARHGAP42*	protein_coding
rs11224447	T	intron_variant	MODIFIER	*ARHGAP42*	protein_coding
rs622675	A	intron_variant	MODIFIER	*ARHGAP42*	protein_coding
rs77036212	A	intron_variant	MODIFIER	*ARHGAP42*	protein_coding
rs5794069	A	intron_variant	MODIFIER	*ARHGAP42*	protein_coding
rs662449	T	intron_variant	MODIFIER	*ARHGAP42*	protein_coding
rs11224450	A	intron_variant	MODIFIER	*ARHGAP42*	protein_coding
rs35472062	T	intron_variant	MODIFIER	*ARHGAP42*	protein_coding
rs3858412	A	intron_variant	MODIFIER	*ARHGAP42*	protein_coding
rs541931	G	intron_variant	MODIFIER	*ARHGAP42*	protein_coding
rs58232316	G	intron_variant	MODIFIER	*ARHGAP42*	protein_coding
rs546584	C	intron_variant	MODIFIER	*ARHGAP42*	protein_coding
rs12793858	A	intron_variant	MODIFIER	*ARHGAP42*	protein_coding
rs11823529	G	intron_variant	MODIFIER	*ARHGAP42*	protein_coding
rs61999341	A	synonymous_variant	LOW	*ARHGAP42*	protein_coding
rs61890783	A	intron_variant	MODIFIER	*ARHGAP42*	protein_coding
rs11224457	C	intron_variant	MODIFIER	*ARHGAP42*	protein_coding
rs537439	G	intron_variant	MODIFIER	*ARHGAP42*	protein_coding
rs2155140	A	intron_variant	MODIFIER	*ARHGAP42*	protein_coding
rs73007660	G	intron_variant	MODIFIER	*ARHGAP42*	protein_coding
rs614615	C	intron_variant	MODIFIER	*ARHGAP42*	protein_coding
rs17651772	G	intron_variant	MODIFIER	*ARHGAP42*	protein_coding
rs113672675	AGGCTAG	intron_variant	MODIFIER	*ARHGAP42*	protein_coding

rs61890784	A	intron_variant	MODIFIER	*ARHGAP42*	protein_coding
rs116659446	A	intron_variant	MODIFIER	*ARHGAP42*	protein_coding
rs79065387	T	intron_variant	MODIFIER	*ARHGAP42*	protein_coding
rs74694325	A	intron_variant	MODIFIER	*ARHGAP42*	protein_coding
rs528494	G	intron_variant	MODIFIER	*ARHGAP42*	protein_coding
rs55760506	A	intron_variant	MODIFIER	*ARHGAP42*	protein_coding
rs7941077	A	intron_variant	MODIFIER	*ARHGAP42*	protein_coding
rs76787621	C	intron_variant	MODIFIER	*ARHGAP42*	protein_coding
rs5794070	AAAAAA	intron_variant	MODIFIER	*ARHGAP42*	protein_coding
rs74565354	T	intron_variant	MODIFIER	*ARHGAP42*	protein_coding
rs618291	A	intron_variant	MODIFIER	*ARHGAP42*	protein_coding
rs79735088	G	intron_variant	MODIFIER	*ARHGAP42*	protein_coding
rs201578748	CC	intron_variant	MODIFIER	*ARHGAP42*	protein_coding
rs61890786	A	intron_variant	MODIFIER	*ARHGAP42*	protein_coding
rs113692841	A	intron_variant	MODIFIER	*ARHGAP42*	protein_coding
rs76169825	C	intron_variant	MODIFIER	*ARHGAP42*	protein_coding
rs11824259	T	intron_variant	MODIFIER	*ARHGAP42*	protein_coding
rs12419467	A	intron_variant	MODIFIER	*ARHGAP42*	protein_coding
rs509944	G	intron_variant	MODIFIER	*ARHGAP42*	protein_coding
rs598702	T	intron_variant	MODIFIER	*ARHGAP42*	protein_coding
rs10791427	T	intron_variant	MODIFIER	*ARHGAP42*	protein_coding
rs12417529	T	intron_variant	MODIFIER	*ARHGAP42*	protein_coding
rs660338	T	intron_variant	MODIFIER	*ARHGAP42*	protein_coding
rs680604	C	intron_variant	MODIFIER	*ARHGAP42*	protein_coding
rs4754701	A	intron_variant	MODIFIER	*ARHGAP42*	protein_coding
rs11224467	A	intron_variant	MODIFIER	*ARHGAP42*	protein_coding
rs616680	G	intron_variant	MODIFIER	*ARHGAP42*	protein_coding
rs11224469	T	intron_variant	MODIFIER	*ARHGAP42*	protein_coding
rs586495	C	intron_variant	MODIFIER	*ARHGAP42*	protein_coding
rs17652307	G	intron_variant	MODIFIER	*ARHGAP42*	protein_coding
rs678681	C	intron_variant	MODIFIER	*ARHGAP42*	protein_coding

rs11224471	A	intron_variant	MODIFIER	*ARHGAP42*	protein_coding
rs11224472	G	intron_variant	MODIFIER	*ARHGAP42*	protein_coding
rs11224473	G	intron_variant	MODIFIER	*ARHGAP42*	protein_coding
rs650867	A	intron_variant	MODIFIER	*ARHGAP42*	protein_coding
rs556146	G	intron_variant	MODIFIER	*ARHGAP42*	protein_coding
rs117387764	A	intron_variant	MODIFIER	*ARHGAP42*	protein_coding
rs11224474	T	intron_variant	MODIFIER	*ARHGAP42*	protein_coding
rs2897755	G	intron_variant	MODIFIER	*ARHGAP42*	protein_coding
rs78169527	C	intron_variant	MODIFIER	*ARHGAP42*	protein_coding
rs629864	T	intron_variant	MODIFIER	*ARHGAP42*	protein_coding
rs561267	G	intron_variant	MODIFIER	*ARHGAP42*	protein_coding
rs12785918	G	intron_variant	MODIFIER	*ARHGAP42*	protein_coding
rs12809048	T	intron_variant	MODIFIER	*ARHGAP42*	protein_coding
rs117840491	C	intron_variant	MODIFIER	*ARHGAP42*	protein_coding
rs12295392	A	intron_variant	MODIFIER	*ARHGAP42*	protein_coding
rs73009603	T	intron_variant	MODIFIER	*ARHGAP42*	protein_coding
rs4754705	A	intron_variant	MODIFIER	*ARHGAP42*	protein_coding
rs55868772	A	intron_variant	MODIFIER	*ARHGAP42*	protein_coding
rs142071106	C	intron_variant	MODIFIER	*ARHGAP42*	protein_coding
rs12361074	C	intron_variant	MODIFIER	*ARHGAP42*	protein_coding
rs147014235	GTG	intron_variant	MODIFIER	*ARHGAP42*	protein_coding
rs527476891	G	intron_variant	MODIFIER	*ARHGAP42*	protein_coding
rs76314809	G	intron_variant	MODIFIER	*CNTN5*	protein_coding
rs11220280	T	intron_variant	MODIFIER	*CNTN5*	protein_coding
rs10501915	T	intron_variant	MODIFIER	*CNTN5*	protein_coding
rs10790767	T	intron_variant	MODIFIER	*CNTN5*	protein_coding
rs10790768	T	intron_variant	MODIFIER	*CNTN5*	protein_coding
rs10750310	G	intron_variant	MODIFIER	*CNTN5*	protein_coding
rs10893455	G	intron_variant	MODIFIER	*CNTN5*	protein_coding
rs10893456	G	intron_variant	MODIFIER	*CNTN5*	protein_coding
rs149803865	TTTAATTTA	intron_variant	MODIFIER	*CNTN5*	protein_coding

rs10750313	A	intron_variant	MODIFIER	*CNTN5*	protein_coding
rs10893461	T	intron_variant	MODIFIER	*CNTN5*	protein_coding
rs10790772	C	intron_variant	MODIFIER	*CNTN5*	protein_coding
rs10790773	A	intron_variant	MODIFIER	*CNTN5*	protein_coding
rs71475569	GTCTAGGTCTAGG	intron_variant	MODIFIER	*CNTN5*	protein_coding
rs10893462	G	intron_variant	MODIFIER	*CNTN5*	protein_coding
rs10790774	C	intron_variant	MODIFIER	*CNTN5*	protein_coding
rs11220319	A	intron_variant	MODIFIER	*CNTN5*	protein_coding
rs11532028	G	intron_variant	MODIFIER	*CNTN5*	protein_coding
rs113238743	A	intron_variant	MODIFIER	*CNTN5*	protein_coding
rs71474525	G	intron_variant	MODIFIER	*CNTN5*	protein_coding
rs10893463	A	intron_variant	MODIFIER	*CNTN5*	protein_coding
rs10750314	G	intron_variant	MODIFIER	*CNTN5*	protein_coding
rs10750315	G	intron_variant	MODIFIER	*CNTN5*	protein_coding
rs11220327	G	intron_variant	MODIFIER	*CNTN5*	protein_coding
rs10790775	G	intron_variant	MODIFIER	*CNTN5*	protein_coding
rs10893467	C	intron_variant	MODIFIER	*CNTN5*	protein_coding
rs10893468	C	intron_variant	MODIFIER	*CNTN5*	protein_coding
rs10893469	A	intron_variant	MODIFIER	*CNTN5*	protein_coding
rs10893470	G	intron_variant	MODIFIER	*CNTN5*	protein_coding
rs11220331	A	intron_variant	MODIFIER	*CNTN5*	protein_coding
rs10893472	C	intron_variant	MODIFIER	*CNTN5*	protein_coding
rs10750316	T	intron_variant	MODIFIER	*CNTN5*	protein_coding
rs10790776	A	intron_variant	MODIFIER	*CNTN5*	protein_coding
rs10790777	T	intron_variant	MODIFIER	*CNTN5*	protein_coding
rs11220342	G	intron_variant	MODIFIER	*CNTN5*	protein_coding
rs10893477	G	intron_variant	MODIFIER	*CNTN5*	protein_coding
rs10790778	A	intron_variant	MODIFIER	*CNTN5*	protein_coding
rs10790779	T	intron_variant	MODIFIER	*CNTN5*	protein_coding
rs10750317	G	intron_variant	MODIFIER	*CNTN5*	protein_coding
rs10790780	C	intron_variant	MODIFIER	*CNTN5*	protein_coding

rs10893478	G	intron_variant	MODIFIER	*CNTN5*	protein_coding
rs10790781	A	intron_variant	MODIFIER	*CNTN5*	protein_coding
rs10790782	T	intron_variant	MODIFIER	*CNTN5*	protein_coding
rs10893479	G	intron_variant	MODIFIER	*CNTN5*	protein_coding
rs10893480	C	intron_variant	MODIFIER	*CNTN5*	protein_coding
rs10790785	A	intron_variant	MODIFIER	*CNTN5*	protein_coding
rs10736549	G	intron_variant	MODIFIER	*CNTN5*	protein_coding
rs10893481	C	intron_variant	MODIFIER	*CNTN5*	protein_coding
rs10790786	T	intron_variant	MODIFIER	*CNTN5*	protein_coding
rs10750320	C	intron_variant	MODIFIER	*CNTN5*	protein_coding
rs10893482	A	intron_variant	MODIFIER	*CNTN5*	protein_coding
rs10790788	C	intron_variant	MODIFIER	*CNTN5*	protein_coding
rs12279785	G	intron_variant	MODIFIER	*CNTN5*	protein_coding
rs10736550	A	intron_variant	MODIFIER	*CNTN5*	protein_coding
rs10790789	A	intron_variant	MODIFIER	*CNTN5*	protein_coding
rs61054860	CATGTAAACATGTAAA	intron_variant	MODIFIER	*CNTN5*	protein_coding
rs7925532	C	intron_variant	MODIFIER	*CNTN5*	protein_coding
rs7936053	G	intron_variant	MODIFIER	*CNTN5*	protein_coding
rs11220772	A	intron_variant	MODIFIER	*CNTN5*	protein_coding
rs7122777	A	intron_variant	MODIFIER	*CNTN5*	protein_coding
rs606249	A	intron_variant	MODIFIER	*CNTN5*	protein_coding
rs655610	C	intron_variant	MODIFIER	*CNTN5*	protein_coding
rs584931	A	intron_variant	MODIFIER	*CNTN5*	protein_coding
rs7970812	C	intron_variant	MODIFIER	*SLC2A13*	protein_coding
rs11174702	C	intron_variant	MODIFIER	*SLC2A13*	protein_coding
rs4625545	A	intron_variant	MODIFIER	*SLC2A13*	protein_coding
rs7313209	T	intron_variant	MODIFIER	*SLC2A13*	protein_coding
rs7959498	G	intron_variant	MODIFIER	*SLC2A13*	protein_coding
rs7313231	T	intron_variant	MODIFIER	*SLC2A13*	protein_coding
rs7139216	A	intron_variant	MODIFIER	*SLC2A13*	protein_coding
rs2128275	C	intron_variant	MODIFIER	*SLC2A13*	protein_coding

rs10784338	T	intron_variant	MODIFIER	*SLC2A13*	protein_coding
rs11174809	T	intron_variant	MODIFIER	*SLC2A13*	protein_coding
rs11174812	T	intron_variant	MODIFIER	*SLC2A13*	protein_coding
rs11174813	C	intron_variant	MODIFIER	*SLC2A13*	protein_coding
rs2896825	A	intron_variant	MODIFIER	*SLC2A13*	protein_coding
rs10784359	T	intron_variant	MODIFIER	*SLC2A13*	protein_coding
rs1873610	C	downstream_gene_variant	MODIFIER	*LINC02555*	lncRNA
rs2638222	A	upstream_gene_variant	MODIFIER	*LINC02555*	lncRNA
rs116313537	T	upstream_gene_variant	MODIFIER	*LINC02555*	lncRNA
rs79179868	A	intron_variant,non_coding_transcript_variant	MODIFIER	*LINC02471*	lncRNA
rs1145041	T	intron_variant	MODIFIER	*CNTN4*	protein_coding
rs4685508	A	intron_variant	MODIFIER	*CNTN4*	protein_coding
rs7639586	T	intron_variant	MODIFIER	*CNTN4*	protein_coding
rs1627079	A	intron_variant	MODIFIER	*CNTN4*	protein_coding
rs1666326	G	intron_variant	MODIFIER	*CNTN4*	protein_coding
rs1720179	A	intron_variant	MODIFIER	*CNTN4*	protein_coding
rs1685491	C	intron_variant	MODIFIER	*CNTN4*	protein_coding
rs4685515	A	intron_variant	MODIFIER	*CNTN4*	protein_coding
rs12639121	G	intron_variant	MODIFIER	*CNTN4*	protein_coding
rs1685492	G	intron_variant	MODIFIER	*CNTN4*	protein_coding
rs1554562	C	intron_variant	MODIFIER	*CNTN4*	protein_coding
rs1566382	G	intron_variant	MODIFIER	*CNTN4*	protein_coding
rs3856836	C	intron_variant	MODIFIER	*CNTN4*	protein_coding
rs1666329	T	intron_variant	MODIFIER	*CNTN4*	protein_coding
rs3856837	T	intron_variant	MODIFIER	*CNTN4*	protein_coding
rs3883937	G	intron_variant	MODIFIER	*CNTN4*	protein_coding
rs3856838	C	intron_variant	MODIFIER	*CNTN4*	protein_coding
rs1617892	T	intron_variant	MODIFIER	*CNTN4*	protein_coding
rs1666331	G	intron_variant	MODIFIER	*CNTN4*	protein_coding
rs2728517	A	intron_variant	MODIFIER	*CNTN4*	protein_coding
rs1685495	A	intron_variant	MODIFIER	*CNTN4*	protein_coding

rs4684343	A	intron_variant	MODIFIER	*CNTN4*	protein_coding
rs200861416	GG	intron_variant	MODIFIER	*CNTN4*	protein_coding
rs968057	A	intron_variant	MODIFIER	*CNTN4*	protein_coding
rs4685517	G	intron_variant	MODIFIER	*CNTN4*	protein_coding
rs4684345	G	intron_variant	MODIFIER	*CNTN4*	protein_coding
rs4685518	A	intron_variant	MODIFIER	*CNTN4*	protein_coding
rs58360846	C	intron_variant	MODIFIER	*CNTN4*	protein_coding
rs113521342	A	intron_variant	MODIFIER	*CNTN4*	protein_coding
rs13093013	T	intron_variant	MODIFIER	*CNTN4*	protein_coding
rs13093020	T	intron_variant	MODIFIER	*CNTN4*	protein_coding
rs13091719	A	intron_variant	MODIFIER	*CNTN4*	protein_coding
rs13091946	C	intron_variant	MODIFIER	*CNTN4*	protein_coding
rs3913590	C	intron_variant	MODIFIER	*CNTN4*	protein_coding
rs77917680	G	intron_variant	MODIFIER	*CNTN4*	protein_coding
rs58529380	G	intron_variant	MODIFIER	*CNTN4*	protein_coding
rs57756639	G	intron_variant	MODIFIER	*CNTN4*	protein_coding
rs12496950	G	intron_variant	MODIFIER	*CNTN4*	protein_coding
rs6792956	C	intron_variant	MODIFIER	*CNTN4*	protein_coding
rs6795825	A	intron_variant	MODIFIER	*CNTN4*	protein_coding
rs76345211	A	intron_variant	MODIFIER	*CNTN4*	protein_coding
rs12632872	G	intron_variant	MODIFIER	*CNTN4*	protein_coding
rs559108268	TGT	intron_variant	MODIFIER	*CNTN4*	protein_coding
rs4685542	C	intron_variant	MODIFIER	*CNTN4*	protein_coding
rs2320959	C	intron_variant	MODIFIER	*CNTN4*	protein_coding
rs373714481	TTATTTACTTATTTA	intron_variant	MODIFIER	*CNTN4*	protein_coding
rs6790416	G	intron_variant	MODIFIER	*CNTN4*	protein_coding
rs6793002	C	intron_variant	MODIFIER	*CNTN4*	protein_coding
rs908495	C	intron_variant	MODIFIER	*CNTN4*	protein_coding
rs2320958	A	intron_variant	MODIFIER	*CNTN4*	protein_coding
rs908494	A	intron_variant	MODIFIER	*CNTN4*	protein_coding
rs2320957	C	intron_variant	MODIFIER	*CNTN4*	protein_coding

rs144052920	TAAA	intron_variant	MODIFIER	*CNTN4*	protein_coding
rs58942797	C	intron_variant	MODIFIER	*CNTN4*	protein_coding
rs4685546	G	intron_variant	MODIFIER	*CNTN4*	protein_coding
rs12631108	A	intron_variant	MODIFIER	*CNTN4*	protein_coding
rs62232818	T	intron_variant	MODIFIER	*CNTN4*	protein_coding

STable 7: all eQTL SNPs in the CNTN family

Gene Symbol	Chr	A1	SNP Id	NES	P-Value	Tissue	A1	Beta WHR adj BMI
CNTN2	1	C	rs10494860	0.44	1.40E-10	Skin - Not Sun Exposed (Suprapubic)	G	0.001
CNTN2	1	C	rs10494860	0.34	4.00E-08	Skin - Sun Exposed (Lower leg)	G	0.001
CNTN2	1	C	rs1061132	0.41	2.00E-09	Skin - Not Sun Exposed (Suprapubic)	A	0.001
CNTN2	1	C	rs1061132	0.32	1.50E-07	Skin - Sun Exposed (Lower leg)	A	0.001
CNTN2	1	G	rs1062715	0.44	2.40E-10	Skin - Not Sun Exposed (Suprapubic)	A	0.001
CNTN2	1	G	rs1062715	0.33	6.70E-08	Skin - Sun Exposed (Lower leg)	A	0.001
CNTN2	1	A	rs10793727	-0.41	2.20E-08	Esophagus - Muscularis	A	-0.001
CNTN2	1	A	rs10793727	-0.27	6.00E-05	Nerve - Tibial	A	-0.001
CNTN2	1	A	rs10793727	-0.55	1.00E-10	Thyroid	A	-0.001
CNTN2	1	C	rs10900444	-0.41	6.10E-09	Esophagus - Muscularis	C	-0.001
CNTN2	1	C	rs10900444	-0.32	4.50E-07	Nerve - Tibial	C	-0.001
CNTN2	1	C	rs10900444	-0.82	2.10E-06	Spleen	C	-0.001
CNTN2	1	C	rs10900444	-0.56	4.90E-12	Thyroid	C	-0.001
CNTN2	1	C	rs10900458	-0.41	2.20E-08	Esophagus - Muscularis	C	-0.001
CNTN2	1	C	rs10900458	-0.27	6.00E-05	Nerve - Tibial	C	-0.001
CNTN2	1	C	rs10900458	-0.55	1.00E-10	Thyroid	C	-0.001
CNTN2	1	T	rs1172122	0.38	7.60E-08	Esophagus - Muscularis	C	-0.001
CNTN2	1	T	rs1172122	0.51	9.50E-10	Thyroid	C	-0.001
CNTN2	1	C	rs1172123	0.38	8.40E-08	Esophagus - Muscularis	G	-0.001
CNTN2	1	C	rs1172123	0.52	3.60E-10	Thyroid	G	-0.001
CNTN2	1	A	rs1172128	-0.52	2.70E-07	Thyroid	A	-0.001
CNTN2	1	A	rs1172134	-0.37	3.60E-06	Esophagus - Muscularis	A	-0.001
CNTN2	1	A	rs1172134	-0.54	1.80E-08	Thyroid	A	-0.001
CNTN2	1	C	rs11804620	0.43	6.40E-10	Skin - Not Sun Exposed (Suprapubic)	A	0.001
CNTN2	1	C	rs11804620	0.33	1.10E-07	Skin - Sun Exposed (Lower leg)	A	0.001
CNTN2	1	G	rs11808879	0.41	4.30E-09	Skin - Not Sun Exposed (Suprapubic)	A	0.001
CNTN2	1	G	rs11808879	0.31	7.70E-07	Skin - Sun Exposed (Lower leg)	A	0.001

CNTN2	1	T	rs11811115	0.43	2.80E-10	Skin - Not Sun Exposed (Suprapubic)	C	0.001
CNTN2	1	T	rs11811115	0.33	7.30E-08	Skin - Sun Exposed (Lower leg)	C	0.001
CNTN2	1	T	rs12022384	0.40	1.10E-06	Skin - Not Sun Exposed (Suprapubic)	C	0.001
CNTN2	1	T	rs12022384	0.33	6.90E-06	Skin - Sun Exposed (Lower leg)	C	0.001
CNTN2	1	A	rs12023022	-0.41	2.20E-08	Esophagus - Muscularis	A	-0.001
CNTN2	1	A	rs12023022	-0.27	6.00E-05	Nerve - Tibial	A	-0.001
CNTN2	1	A	rs12023022	-0.55	1.00E-10	Thyroid	A	-0.001
CNTN2	1	T	rs12025836	0.42	4.30E-08	Skin - Not Sun Exposed (Suprapubic)	C	0.001
CNTN2	1	T	rs12025836	0.30	2.10E-05	Skin - Sun Exposed (Lower leg)	C	0.001
CNTN2	1	T	rs12027259	0.41	9.00E-08	Skin - Not Sun Exposed (Suprapubic)	C	0.001
CNTN2	1	T	rs12027259	0.30	2.00E-05	Skin - Sun Exposed (Lower leg)	C	0.001
CNTN2	1	C	rs12033493	0.43	4.00E-08	Skin - Not Sun Exposed (Suprapubic)	T	0.001
CNTN2	1	C	rs12033493	0.30	1.80E-05	Skin - Sun Exposed (Lower leg)	T	0.001
CNTN2	1	G	rs12039805	0.39	8.60E-09	Skin - Not Sun Exposed (Suprapubic)	A	0.001
CNTN2	1	G	rs12039805	0.31	3.80E-07	Skin - Sun Exposed (Lower leg)	A	0.001
CNTN2	1	T	rs12048416	0.43	2.80E-10	Skin - Not Sun Exposed (Suprapubic)	C	0.001
CNTN2	1	T	rs12048416	0.33	7.30E-08	Skin - Sun Exposed (Lower leg)	C	0.001
CNTN2	1	T	rs12048453	0.44	1.60E-10	Skin - Not Sun Exposed (Suprapubic)	C	0.001
CNTN2	1	T	rs12048453	0.33	5.20E-08	Skin - Sun Exposed (Lower leg)	C	0.001
CNTN2	1	G	rs12078075	-0.37	4.30E-06	Esophagus - Muscularis	G	-0.001
CNTN2	1	G	rs12078075	-0.56	9.20E-09	Thyroid	G	-0.001
CNTN2	1	T	rs12728202	-0.39	6.30E-09	Esophagus - Muscularis	T	-0.001
CNTN2	1	T	rs12728202	-0.48	9.80E-10	Thyroid	T	-0.001
CNTN2	1	G	rs1339321	0.45	5.90E-09	Skin - Not Sun Exposed (Suprapubic)	A	0.001
CNTN2	1	G	rs1339321	0.31	6.90E-06	Skin - Sun Exposed (Lower leg)	A	0.001
CNTN2	1	C	rs1572995	-0.32	3.40E-06	Artery - Tibial	C	-0.001
CNTN2	1	C	rs1572995	-0.40	1.30E-08	Esophagus - Muscularis	C	-0.001
CNTN2	1	C	rs1572995	-0.27	1.40E-05	Nerve - Tibial	C	-0.001
CNTN2	1	C	rs1572995	-1.00	1.50E-08	Spleen	C	-0.001
CNTN2	1	C	rs1572995	-0.59	1.60E-12	Thyroid	C	-0.001
CNTN2	1	A	rs1668874	-0.40	6.40E-08	Esophagus - Muscularis	A	-0.001

CNTN2	1	A	rs1668874	-0.55	1.30E-10	Thyroid	A	-0.001
CNTN2	1	T	rs16855154	0.44	8.30E-11	Skin - Not Sun Exposed (Suprapubic)	C	0.001
CNTN2	1	T	rs16855154	0.34	2.70E-08	Skin - Sun Exposed (Lower leg)	C	0.001
CNTN2	1	T	rs16855186	0.45	3.90E-09	Skin - Not Sun Exposed (Suprapubic)	C	0.001
CNTN2	1	T	rs16855186	0.30	1.60E-05	Skin - Sun Exposed (Lower leg)	C	0.001
CNTN2	1	T	rs17728	-0.37	3.60E-06	Esophagus - Muscularis	T	-0.001
CNTN2	1	T	rs17728	-0.54	1.80E-08	Thyroid	T	-0.001
CNTN2	1	T	rs2369633	-0.38	1.90E-06	Esophagus - Muscularis	T	-0.001
CNTN2	1	T	rs2369633	-0.55	1.30E-08	Thyroid	T	-0.001
CNTN2	1	G	rs2369634	-0.41	8.50E-09	Esophagus - Muscularis	G	-0.001
CNTN2	1	G	rs2369634	-0.28	1.90E-05	Nerve - Tibial	G	-0.001
CNTN2	1	G	rs2369634	-0.51	5.50E-10	Thyroid	G	-0.001
CNTN2	1	A	rs3183297	0.43	4.80E-10	Skin - Not Sun Exposed (Suprapubic)	G	0.001
CNTN2	1	A	rs3183297	0.33	8.50E-08	Skin - Sun Exposed (Lower leg)	G	0.001
CNTN2	1	T	rs3753847	-0.26	2.60E-05	Artery - Tibial	T	-0.001
CNTN2	1	T	rs3753847	-0.33	9.20E-08	Esophagus - Muscularis	T	-0.001
CNTN2	1	T	rs3753847	-0.23	5.80E-05	Nerve - Tibial	T	-0.001
CNTN2	1	T	rs3753847	-0.32	1.60E-05	Skin - Sun Exposed (Lower leg)	T	-0.001
CNTN2	1	T	rs3753847	-0.52	8.00E-12	Thyroid	T	-0.001
CNTN2	1	T	rs3795556	0.44	1.60E-10	Skin - Not Sun Exposed (Suprapubic)	C	0.001
CNTN2	1	T	rs3795556	0.33	5.20E-08	Skin - Sun Exposed (Lower leg)	C	0.001
CNTN2	1	C	rs3851286	-0.41	2.20E-08	Esophagus - Muscularis	C	-0.001
CNTN2	1	C	rs3851286	-0.27	6.00E-05	Nerve - Tibial	C	-0.001
CNTN2	1	C	rs3851286	-0.55	1.00E-10	Thyroid	C	-0.001
CNTN2	1	C	rs3851287	0.45	3.80E-09	Skin - Not Sun Exposed (Suprapubic)	T	0.001
CNTN2	1	C	rs3851287	0.30	9.50E-06	Skin - Sun Exposed (Lower leg)	T	0.001
CNTN2	1	A	rs3851294	-0.39	1.30E-06	Esophagus - Muscularis	A	-0.001
CNTN2	1	A	rs3851294	-0.55	8.70E-09	Thyroid	A	-0.001
CNTN2	1	C	rs3862948	0.44	5.10E-10	Skin - Not Sun Exposed (Suprapubic)	A	0.001
CNTN2	1	C	rs3862948	0.33	9.90E-08	Skin - Sun Exposed (Lower leg)	A	0.001
CNTN2	1	T	rs3903399	0.38	3.30E-07	Skin - Not Sun Exposed (Suprapubic)	C	0.001

CNTN2	1	T	rs3903399	0.28	5.60E-05	Skin - Sun Exposed (Lower leg)	C	0.001
CNTN2	1	A	rs4950984	0.39	5.70E-08	Skin - Not Sun Exposed (Suprapubic)	G	0.001
CNTN2	1	A	rs4950984	0.30	3.40E-06	Skin - Sun Exposed (Lower leg)	G	0.001
CNTN2	1	G	rs4951159	-0.38	2.20E-05	Brain - Cerebellum	G	-0.001
CNTN2	1	G	rs4951159	-0.45	5.70E-08	Esophagus - Muscularis	G	-0.001
CNTN2	1	G	rs4951159	-1.10	3.30E-07	Spleen	G	-0.001
CNTN2	1	G	rs4951159	-0.70	2.10E-12	Thyroid	G	-0.001
CNTN2	1	T	rs4951163	-0.40	3.70E-06	Esophagus - Muscularis	T	-0.001
CNTN2	1	T	rs4951163	-0.94	4.50E-06	Spleen	T	-0.001
CNTN2	1	T	rs4951163	-0.64	2.70E-10	Thyroid	T	-0.001
CNTN2	1	A	rs4951166	-0.43	1.30E-09	Esophagus - Muscularis	A	-0.001
CNTN2	1	A	rs4951166	-0.31	9.60E-07	Nerve - Tibial	A	-0.001
CNTN2	1	A	rs4951166	-0.82	2.10E-06	Spleen	A	-0.001
CNTN2	1	A	rs4951166	-0.56	6.00E-12	Thyroid	A	-0.001
CNTN2	1	G	rs55966786	0.39	3.50E-05	Esophagus - Muscularis	A	-0.001
CNTN2	1	G	rs55966786	0.57	1.10E-07	Thyroid	A	-0.001
CNTN2	1	G	rs56323810	0.39	4.00E-05	Esophagus - Muscularis	A	-0.001
CNTN2	1	G	rs56323810	0.55	3.20E-07	Thyroid	A	-0.001
CNTN2	1	A	rs61822618	0.42	3.00E-08	Skin - Not Sun Exposed (Suprapubic)	T	0.001
CNTN2	1	A	rs61822618	0.30	1.50E-05	Skin - Sun Exposed (Lower leg)	T	0.001
CNTN2	1	G	rs61822621	0.41	1.90E-09	Skin - Not Sun Exposed (Suprapubic)	A	0.001
CNTN2	1	G	rs61822621	0.33	8.20E-08	Skin - Sun Exposed (Lower leg)	A	0.001
CNTN2	1	G	rs61822627	0.43	3.00E-10	Skin - Not Sun Exposed (Suprapubic)	A	0.001
CNTN2	1	G	rs61822627	0.33	5.80E-08	Skin - Sun Exposed (Lower leg)	A	0.001
CNTN2	1	G	rs61822628	0.44	1.50E-10	Skin - Not Sun Exposed (Suprapubic)	C	0.001
CNTN2	1	G	rs61822628	0.33	4.80E-08	Skin - Sun Exposed (Lower leg)	C	0.001
CNTN2	1	C	rs6593930	0.44	1.30E-10	Skin - Not Sun Exposed (Suprapubic)	T	0.001
CNTN2	1	C	rs6593930	0.35	2.00E-08	Skin - Sun Exposed (Lower leg)	T	0.001
CNTN2	1	C	rs6664360	0.41	1.60E-09	Skin - Not Sun Exposed (Suprapubic)	T	0.001
CNTN2	1	C	rs6664360	0.33	1.20E-07	Skin - Sun Exposed (Lower leg)	T	0.001
CNTN2	1	A	rs6684220	-0.40	7.40E-08	Esophagus - Muscularis	A	-0.001

CNTN2	1	A	rs6684220	-0.28	3.00E-05	Nerve - Tibial	A	-0.001
CNTN2	1	A	rs6684220	-0.55	8.40E-11	Thyroid	A	-0.001
CNTN2	1	A	rs6687271	0.43	1.80E-10	Skin - Not Sun Exposed (Suprapubic)	C	0.001
CNTN2	1	A	rs6687271	0.34	2.40E-08	Skin - Sun Exposed (Lower leg)	C	0.001
CNTN2	1	C	rs74787661	0.42	6.30E-08	Skin - Not Sun Exposed (Suprapubic)	A	0.001
CNTN2	1	C	rs74787661	0.30	1.50E-05	Skin - Sun Exposed (Lower leg)	A	0.001
CNTN2	1	A	rs7513016	-0.33	9.10E-06	Esophagus - Muscularis	A	-0.001
CNTN2	1	A	rs7513016	-0.58	3.90E-10	Thyroid	A	-0.001
CNTN2	1	T	rs7514499	0.42	4.30E-08	Skin - Not Sun Exposed (Suprapubic)	C	0.001
CNTN2	1	T	rs7514499	0.30	1.70E-05	Skin - Sun Exposed (Lower leg)	C	0.001
CNTN2	1	G	rs7529665	0.41	1.90E-09	Skin - Not Sun Exposed (Suprapubic)	A	0.001
CNTN2	1	G	rs7529665	0.33	1.30E-07	Skin - Sun Exposed (Lower leg)	A	0.001
CNTN2	1	T	rs7530217	0.41	1.90E-09	Skin - Not Sun Exposed (Suprapubic)	A	0.001
CNTN2	1	T	rs7530217	0.33	8.20E-08	Skin - Sun Exposed (Lower leg)	A	0.001
CNTN2	1	T	rs7538328	0.46	6.50E-11	Skin - Not Sun Exposed (Suprapubic)	C	0.001
CNTN2	1	T	rs7538328	0.33	1.00E-07	Skin - Sun Exposed (Lower leg)	C	0.001
CNTN2	1	G	rs896319	-0.37	4.40E-06	Esophagus - Muscularis	G	-0.001
CNTN2	1	G	rs896319	-0.56	8.40E-09	Thyroid	G	-0.001
CNTN2	1	C	rs896321	0.38	8.40E-08	Esophagus - Muscularis	G	-0.001
CNTN2	1	C	rs896321	0.51	9.50E-10	Thyroid	G	-0.001
CNTN2	1	A	rs896322	-0.41	2.20E-08	Esophagus - Muscularis	A	-0.001
CNTN2	1	A	rs896322	-0.27	6.00E-05	Nerve - Tibial	A	-0.001
CNTN2	1	A	rs896322	-0.55	1.00E-10	Thyroid	A	-0.001
CNTN2	1	A	rs9651227	-0.39	6.90E-09	Esophagus - Muscularis	A	-0.001
CNTN2	1	A	rs9651227	-0.30	4.10E-07	Nerve - Tibial	A	-0.001
CNTN2	1	A	rs9651227	-0.76	3.60E-06	Spleen	A	-0.001
CNTN2	1	A	rs9651227	-0.53	3.30E-11	Thyroid	A	-0.001
CNTN2	1	A	rs9659978	-0.37	3.60E-06	Esophagus - Muscularis	A	-0.001
CNTN2	1	A	rs9659978	-0.54	1.80E-08	Thyroid	A	-0.001

STable 8: Genotype-specific expression of *ARHGAP42* and *TMEM133*

SNP Id	Variant Id	Gene Symbol	Tissue	NES	P-Value
rs633185	chr11_100722807_G_C	*ARHGAP42*	Adipose - Subcutaneous	-0.14	2.00E-05
			Adipose - Visceral (Omentum)	-0.18	1.50E-05
			Artery - Aorta	-0.54	6.60E-26
			Artery - Coronary	-0.52	1.90E-13
			Artery - Tibial	-0.56	5.80E-47
			Heart - Atrial Appendage	-0.25	2.30E-07
			Heart - Left Ventricle	-0.16	8.40E-05
			Muscle - Skeletal	-0.18	2.90E-06
			Skin - Sun Exposed (Lower leg)	-0.17	5.20E-06
		TMEM133	Adipose - Subcutaneous	-0.19	1.60E-07
			Adipose - Visceral (Omentum)	-0.2	4.70E-06
			Artery - Aorta	-0.55	7.00E-23
			Artery - Coronary	-0.64	1.80E-15
			Artery - Tibial	-0.61	2.50E-49
			Heart - Atrial Appendage	-0.29	1.10E-06
			Muscle - Skeletal	-0.16	1.10E-04
			Nerve - Tibial	-0.096	1.50E-04
rs11606890	chr11_100600735_A_C	*ARHGAP42*	Adipose - Subcutaneous	-0.2	8.50E-05
			Adipose - Visceral (Omentum)	-0.25	4.30E-05
			Artery - Tibial	-0.32	3.20E-07
			Skin - Sun Exposed (Lower leg)	-0.27	1.90E-07
			Thyroid	-0.25	3.30E-06
		CTD-2383M3.1	Skin - Not Sun Exposed (Suprapubic)	-0.23	1.70E-04
			Skin - Sun Exposed (Lower leg)	-0.24	3.20E-05
		TMEM133	Adipose - Visceral (Omentum)	-0.28	2.40E-05
			Artery - Tibial	-0.37	4.20E-08
			Esophagus - Mucosa	-0.25	1.90E-04
			Skin - Sun Exposed (Lower leg)	-0.27	3.30E-06
